# Land snails and slugs of Bau limestone hills, Sarawak (Malaysia, Borneo), with the descriptions of 13 new species

**DOI:** 10.3897/zookeys.1035.60843

**Published:** 2021-04-27

**Authors:** Mohammad Effendi bin Marzuki, Thor-Seng Liew, Jayasilan Mohd-Azlan

**Affiliations:** 1 Institute of Biodiversity and Environmental Conservation, Universiti Malaysia Sarawak, 94300, Kota Samarahan, Sarawak, Malaysia Universiti Malaysia Sarawak Kota Samarahan Malaysia; 2 Institute for Tropical Biology and Conservation, Universiti Malaysia Sabah, Jalan UMS, 88450, Kota Kinabalu, Sabah, Malaysia Universiti Malaysia Sabah Kota Kinabalu Malaysia

**Keywords:** Endemism, Karst ecosystem, Peninsular Malaysia, Sabah, species diversity, tropical rain forest

## Abstract

This study presents a list of land snails and slugs found on limestone hills in the District of Bau, the state of Sarawak in Malaysian Borneo. Systematic and random sampling for land snails was conducted at eight limestone outcrops, namely, Gunung Stulang, Padang Pan, Gunung Kapor, Gunung Lobang Angin, Gunung Doya, Gunung Batu, Bukit Sekunyit and Gunung Sebayat. A total of 122 land snail species was documented with photographs of each species. Of the 122 species collected, 13 are new to science, namely, *Acmella bauensis***sp. nov.**, *Japonia bauensis***sp. nov.**, *Plectostoma margaretchanae***sp. nov.**, *Microcystina arabii***sp. nov.**, *Microcystina atoni***sp. nov.**, *Microcystina paripari***sp. nov.**, *Microcystina lirata***sp. nov.**, *Microcystina oswaldbrakeni***sp. nov.**, *Microcystina kilat***sp. nov.**, *Philalanka jambusanensis***sp. nov.**, *Everettia microrhytida***sp. nov.**, *Everettia minuta***sp. nov.**, and *Paralaoma sarawakensis***sp. nov.**

## Introduction

Limestone hills in Borneo are a hotspot for land snail diversity and thus have been the focus of land snail diversity studies (Vermeulen 1991a, b, 1993, 1994, 1996). Some of the earliest work on land snails in Borneo were initiated at Sarawak’s limestone hills. Recently, a few land snail inventory studies were conducted in a several limestone hills and clusters in Sarawak, for example, in Bukit Sarang (83 species), the Bau-Serian limestone range (148 species), Gunung Mulu (97 species), and Niah (108 species) (Vermeulen and Junau 2007). However, these reports lack a systematic checklist with proper illustrations for every species. There are more than 250 limestone hills in Sarawak, of which half are located in the Kuching and Serian Division.

Here, we report an inventory of land snail and slug species from systematic and random sampling surveys at eight limestone hills in Bau region, namely, Gunung Stulang, Padang Pan, Gunung Kapor, Gunung Lobang Angin, Gunung Doya, Gunung Batu, Bukit Sekunyit and Gunung Sebayat. A total of nine standard samplings were conducted in 400 m^2^ plots where the empty and living snails were searched for two person-hours, and five litres of loose topsoil were collected. At the same time, random samplings were done randomly outside of the standard plots. The collected soil samples were dried in the laboratory, and then the micro-shells were extracted from the soil samples. All specimens were identified to species level, and the materials were deposited at Universiti Malaysia Sarawak (**MZU.MOL**) and the private collection (**ME**) of the first author.

We cross-checked MolluscaBase (MolluscaBase, 2021) to confirm the nomenclature and the classification of the species in this checklist. We followed most of the nomenclature and classification suggested by MolluscaBase except for the year of publication of a few species and classification of a few species at genus and family level (Suppl. material 1).

## Results

A total of 1,085 collection lots obtained from the eight limestone hills at the Bau limestone hill cluster was examined. This checklist comprises a total of 122 land snail species belonging to 57 genera and 24 families. The family Diplommatinidae was the most species-rich family recorded in this limestone hill cluster, with 21 recorded species (17%). This was followed by the Cyclophoridae (16 species, 13%) and the Ariophantidae (14 species, 11%). In terms of genera, the most diverse genera were *Microcystina*, *Kaliella*, and *Diplommatina*, with eight species each. Micro-snails (size less than 5 mm) accounted for ca. 63% of the total number of species.

**Figure 1.**
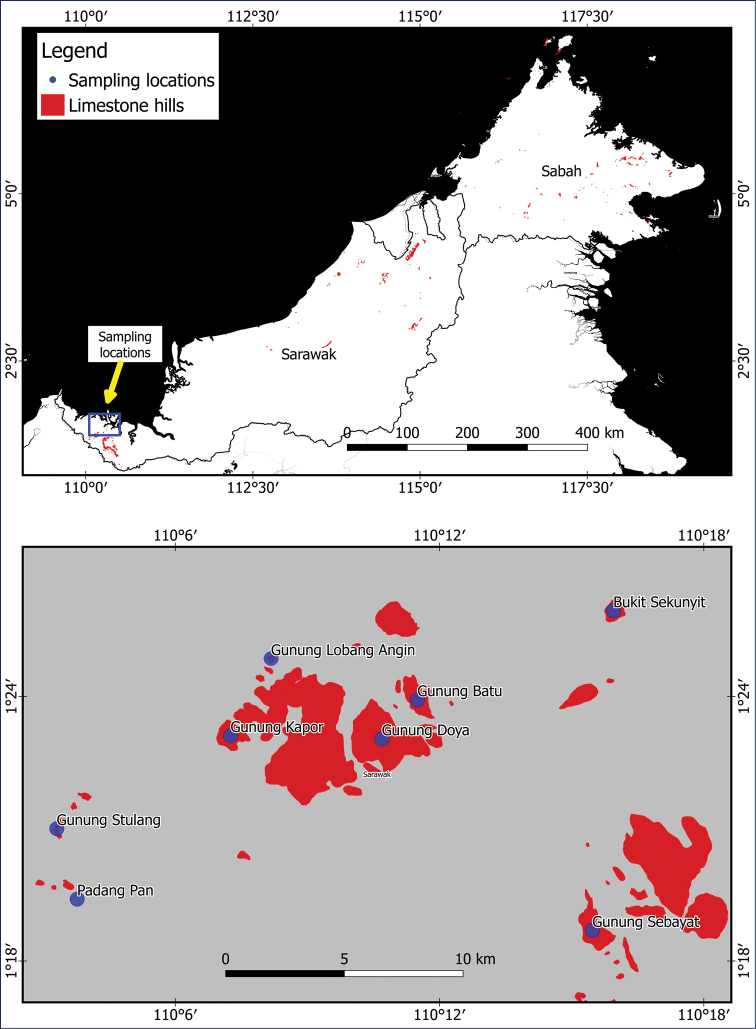
Locations of the eight limestone hills of the Bau area included in this study.

### Checklist

#### Class Gastropoda Cuvier, 1795

**Subclass Caenogastropoda Cox, 1960**

**Family Alycaeidae Blanford, 1864**

##### *Chamalycaeus* Möllendorff, 1897

###### Chamalycaeus specus

Taxon classificationAnimaliaArchitaenioglossaAlycaeidae

(Godwin-Austen, 1889)

6537606F-F6BA-55F6-83D0-6A278415010A

Figures 2A , 48A

Alycaeus specus Godwin-Austen, 1889: 347, pl. 37, figs 4, 4A.

####### Type locality.

“In limestone caves at Jambusan, Borneo” [= Jambusan Hills, Bau, Sarawak].

####### Material examined.

Gunung Sebayat: ME 8005. Gunung Doya: ME 9699, ME 9148, ME 9179. Gunung Kapor: ME 3357, ME 3359, ME 3362, ME 8085, ME 8488, ME 9046, ME 9078, ME 9471. Lobang Angin: ME 9038, ME 9432. Gunung Batu: ME 3351, ME 3352, ME 3353, ME 8802.

####### Distribution in Borneo.

Sarawak: Kuching, Serian, and Kapit divisions. Sabah: Sandakan, Tawau, and West Coast divisions. Endemic to Borneo.

####### Remarks.

Only dry shells were found during the surveys.

**Figure 2.**
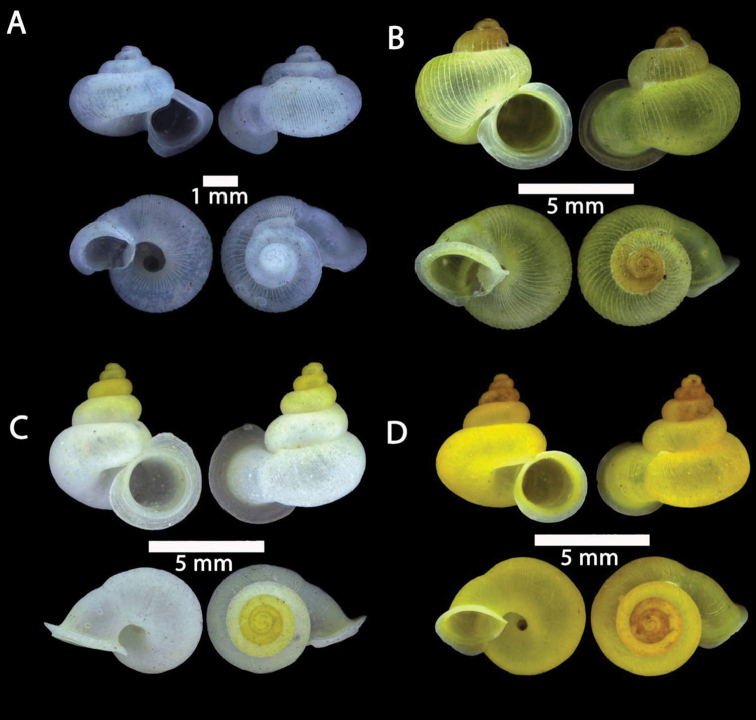
**A***Chamalycaeus specus* (Godwin-Austen, 1889) ME 9046 Gunung Kapor **B***Pincerna globosa* (H. Adams, 1870) ME 6979 Lobang Angin **C***Stomacosmethis hosei* (Godwin-Austen, 1889) ME 1010 Gunung Batu **D***Stomacosmethis sadongensis* (E. A. Smith, 1895) ME 8761 Gunung Kapor.

##### *Pincerna* Preston, 1907

###### Pincerna globosa

Taxon classificationAnimaliaArchitaenioglossaAlycaeidae

(H. Adams, 1870)

A86CAE92-66D2-5196-804B-D09DB6592A63

Figures 2B , 47D

Alycaeus globosus H. Adams, 1870: 794.

####### Type locality.

“Busan, near Sarawak, Borneo” [= Jambusan Hills, Bau, Sarawak].

####### Material examined.

Bukit Sekunyit: ME 1016, ME 6980. Gunung Doya: ME 1059, ME 9698, ME 8958, ME 9090. Gunung Kapor: ME 1004, ME 1015, ME 1053, ME 8489, ME 8974. Gunung Stulang: ME 5904. Lobang Angin: ME 1029, ME 6979, ME 8726, ME 8746, ME 8749, ME 9022. Gunung Batu: ME 1014, ME 1023, ME 1054, ME 8829.

####### Distribution in Borneo.

Sarawak: Kuching, Serian, and Miri divisions. Sabah: West Coast Division. Endemic to Borneo.

####### Remarks.

Smith (1895) classified *Pincerna globosa* into five different forms: *globosa*, *rabongensis*, *muluana*, *kinabaluana*, and *pygmaea*, of which two forms were collected from Bau: *globosa* with a smaller yellowish orange shell while *rabongensis* has a larger yellow shell. The differences between the two forms may due to sexual dimorphism; hence, we considered these forms as synonyms. Living snails were observed foraging on the leaf surfaces of small trees and palms at the base of the limestone cliff.

##### *Stomacosmethis* Bollinger, 1918

###### Stomacosmethis hosei

Taxon classificationAnimaliaArchitaenioglossaAlycaeidae

(Godwin-Austen, 1889)

571E6640-EC3B-5BCE-B91A-EA28F6FBF9BA

Figure 2C

Alycaeus hosei Godwin-Austen, 1889: 347, pl. 37, fig. 2.

####### Type locality.

“Busan Hills, Borneo” [= Jambusan Hills, Bau, Sarawak].

####### Material examined.

Gunung Doya: ME 1088, ME 9133. Gunung Kapor: ME 1024, ME 8084. Gunung Stulang: ME 5903. Gunung Batu: ME 1002, ME 1010, ME 7180, ME 8803.

####### Distribution in Borneo.

Sarawak: Kuching and Serian divisions. Endemic to Borneo.

####### Remarks.

Living snails were observed foraging on the moderately wet vertical limestone rock surfaces that covered with lichens. The shell of this snail is always covered with calcareous dirt.

###### Stomacosmethis sadongensis

Taxon classificationAnimaliaArchitaenioglossaAlycaeidae

(E. A. Smith, 1895)

251DCF31-AE54-558E-9779-A5DCB8FB074A

Figures 2D , 47C

Alycaeus (Orthalycaeus) sadongensis Smith, 1895: 117, pl. 3, fig. 27.

####### Type locality.

“Sadong, Sarawak”.

####### Material examined.

Gunung Kapor: ME 1003, ME 8761. Gunung Batu: ME 2896, ME 2900, ME 8804.

####### Distribution in Borneo.

Sarawak: Kuching and Serian divisions. Endemic to Borneo.

####### Remarks.

This species differs from other Bornean species of *Stomacosmethis* by having a shell with dense, regular, rather low riblets on the shell instead of irregular low riblets. Living snails were observed foraging on wet vertical limestone rock surfaces covered with mosses and lichens.

#### Family Cyclophoridae Gray, 1847

##### *Craspedotropis* W. T. Blanford, 1864

###### Craspedotropis borneensis

Taxon classificationAnimaliaArchitaenioglossaCyclophoridae

(Godwin-Austen, 1889)

DC494095-8F3E-56A7-BDFA-66C893BBD74E

Figure 3A

Jerdonia borneensis Godwin-Austen, 1889: 345–346, pl. 36, figs 6, 6A.

####### Type locality.

“Busan Hills, Borneo” [= Jambusan Hills, Sarawak].

####### Material examined.

Gunung Doya: ME 8909, ME 9182. Gunung Batu: ME 0839, ME 2839.

####### Distribution in Borneo.

Sarawak: Kuching and Miri division. Endemic to Borneo.

####### Remarks.

Only dry shells were found during the surveys.

**Figure 3.**
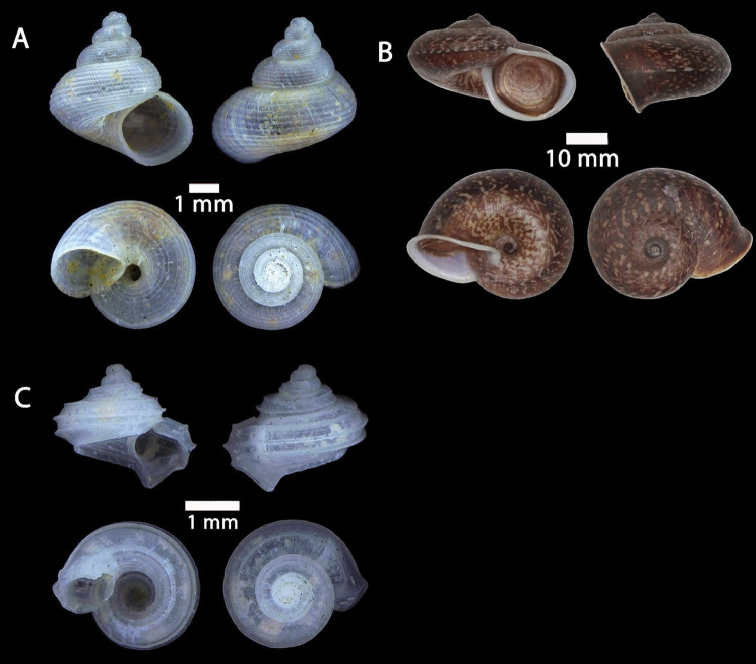
**A***Craspedotropis borneensis* (Godwin-Austen, 1889) ME 839 Gunung Batu **B***Cyclophorus perdix borneensis* (Metcalfe, 1851) ME 8921 Gunung Doya **C***Ditropopsis everetti* (E. A. Smith, 1895) ME 835 Gunung Kapor.

##### *Cyclophorus* Montfort, 1810

###### Cyclophorus perdix borneensis

Taxon classificationAnimaliaArchitaenioglossaCyclophoridae

(Metcalfe, 1851)

26E607D0-5551-5900-BE75-EA610CEDA511

Figures 3B , 47A

Cyclostoma borneensis Metcalfe, 1851: 71.

####### Type locality.

“Borneo”.

####### Material examined.

Gunung Sebayat: ME 8007. Bukit Sekunyit: ME 2631. Gunung Doya: ME 2675, ME 8921, ME 8947, ME 9169. Gunung Kapor: ME 2636, ME 2647, ME 8068, ME 8706, ME 8753, ME 8769, ME 9468. Gunung Stulang: ME 5905. Kampung Bunga Rampai: ME 2610, ME 5947. Kampung Padang Pan: ME 6667. Lobang Angin: ME 2648, ME 9183, ME 9483. Gunung Batu: ME 2633, ME 2642, ME 8805.

####### Distribution in Borneo.

Sarawak: Kuching, Samarahan, Serian, Mukah, Kapit, and Miri divisions. Sabah: West Coast Division. Kalimantan: West, South, and East Kalimantan provinces. ***Distribution elsewhere.*** West Malaysia (Stoliczka 1872; Morgan 1885).

####### Remarks.

Living snails were observed foraging among leaf litter and plant debris near the cliff in lowland limestone forest.

##### *Ditropopsis* E. A. Smith, 1897

###### Ditropopsis everetti

Taxon classificationAnimaliaArchitaenioglossaCyclophoridae

(E. A. Smith, 1895)

8102474F-F60E-588A-BF46-A1F576A290AF

Figure 3C

Cyathopoma everetti E. A. Smith, 1895: 115, pl. 3, figs 21, 22.

####### Type locality.

“Rumbang, Sarawak” [= Rumbang Hills, Padawan, Sarawak].

####### Material examined.

Gunung Sebayat: ME 8009. Gunung Kapor: ME 0835, ME 0842, ME 8494, ME 9079, ME 9239.

####### Distribution in Borneo.

Sarawak: Kuching Division. Endemic to Borneo.

####### Remarks.

Living snails were observed foraging among leaf litter and plant debris near the cliff in a limestone forest. It differs from other Bornean *Ditropopsis* species by having seven distinct lirae on the last whorl (one supra-peripheral, two peripheral, one basal, and three umbilical), instead of 4–6 lirae.

##### *Japonia* A. A. Gould, 1859

###### Japonia barbata

Taxon classificationAnimaliaArchitaenioglossaCyclophoridae

(L. Pfeiffer, 1855)

1763B0AF-ACF5-5B4F-BD96-18323C35AB8C

Figures 4A–B , 46A

Cyclostoma (Leptopoma) barbatum L. Pfeiffer, 1855: 104–105.

####### Type locality.

“Borneo, Sarawak”.

####### Material examined.

Gunung Doya: ME 0817, ME 8903, ME 9092, ME 9668. Gunung Kapor: ME 0737, ME 0780, ME 0784, ME 2949, ME 8069, ME 8490, ME 8768, ME 8783, ME 9047, ME 9609, ME 9841. Lobang Angin: ME 0791, ME 9083, ME 9134, ME 9275. Gunung Batu: ME 0783, ME 0796, ME 0800, ME 8808. Gunung Sebayat: ME 8008. Kampung Bunga Rampai: ME 0833, ME 0834. Kampung Padang Pan: ME 6668.

####### Distribution in Borneo.

Sarawak: Kuching, Samarahan, Serian, Sibu, and Mukah divisions. Endemic to Borneo.

####### Remarks.

**Figure 4.**
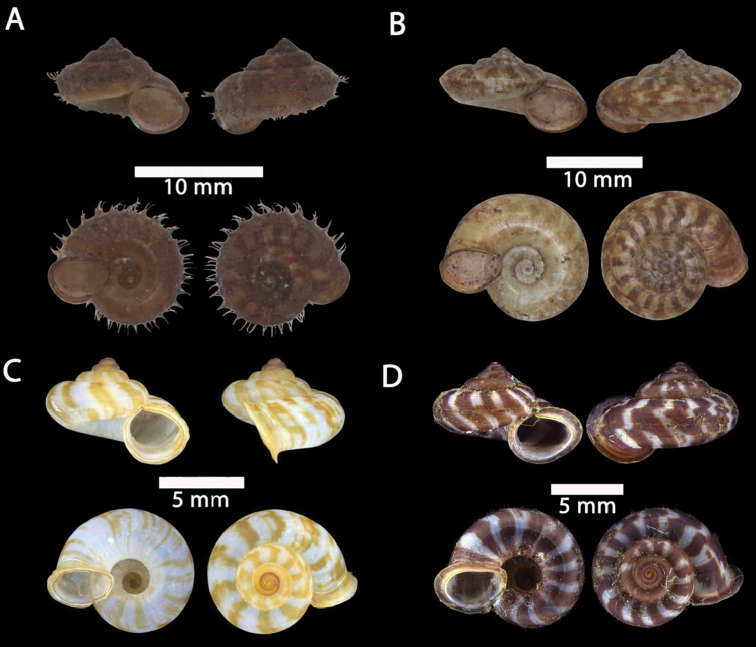
**A***Japonia barbata* (L. Pfeiffer, 1855) ME 833 Kampung Bunga Rampai **B***Japonia barbata* (L. Pfeiffer, 1855) ME 2141 Lobang Angin **C***Japonia bauensis* sp. nov., ME 7231 paratype Gunung Kapor **D***Japonia bauensis* sp. nov., MZU.MOL.20.02 holotype Gunung Doya.

It differs from other Bornean *Japonia* species by the following combination of characters: more depressed medium-sized shell with wide umbilicus, last whorl with distinctly keeled periphery, with long, deciduous, slender, feather-like periostracal hairs along the first peripheral ridge and below the periphery. Shells of different local populations may vary: shells from near Jambusan have a smooth surface with very faint lirae; shells from near Lobang Angin have a distinctly keeled periphery (Fig. 4B); shells from non-limestone areas usually have a somewhat rounded periphery (Fig. 4A). *Japonia similis* (E. A. Smith, 1893) differs by having a larger, high spired shell with moderately wide umbilicus. Living snails were observed foraging among leaf litter and plant debris near the cliff in lowland limestone forest.

###### Japonia bauensis sp. nov.

Taxon classificationAnimaliaArchitaenioglossaCyclophoridae

B115FAA5-B402-5BD7-BC42-87136404E6D5

http://zoobank.org/D7EC642E-FFA0-495E-B506-4A815EF378D0

Figures 4C, D , 5

####### Material examined.

***Holotype*** (SH 5.84 mm, SW 8.00 mm) (MZU.MOL.20.02), Malaysia, Sarawak, Kuching Division, Gunung Doya, limestone hill near Sungai Sebuyoh, 3.4 miles SE Bau, 1°22'57.24"N, 110°11'39.42"E, coll. M. E. Marzuki, 7.I.2018. ***Paratypes***: 1 ex. (ME0000817), the same locality as Holotype, coll. M. E. Marzuki, 10.VII.2011; 1 ex. (ME0009667), Bukit Sokwang (Site 3), northern site of Gunung Doya, limestone hill along Skio road, 2.05 miles E Bau, 1°23'49.87"N, 110°10'32.14"E, coll. M. E. Marzuki, 7.I.2018; 4 ex. (ME0008907), the same locality, coll. M. E. Marzuki, 22.IV.2017; 1 ex. (ME0009167), Bukit Sokwang (Site 2), northern site of Gunung Doya, limestone hill along Skio road, 2.05 miles E Bau, 1°23'45.69"N, 110°10'35.04"E, coll. M. E. Marzuki, 22.IV.2017; >10 ex. (ME0000743), Fairy Caves (Site 1), south part of Gunung Kapor, 4 miles SW Bau, 1°22'53.76"N, 110°7'4.34"E, coll. M. E. Marzuki, 18.VIII.2007; >10 ex. (ME0000779), the same locality, coll. M. E. Marzuki, 11.III.2011; 4 ex. (ME0005974), the same locality, coll. M. E. Marzuki, 21.II.2015; >10 ex. (ME0007231), the same locality, coll. M. E. Marzuki, 27.X.2008; 2 ex. (MZU.MOL.20.03), the same locality, coll. M. E. Marzuki, 27.X.2008; 6 ex. (ME0008491), the same locality, coll. M. E. Marzuki, 10.II.2017; 1 ex. (ME0009216), the same locality, coll. M. E. Marzuki, 8.IV.2017.

####### Differential diagnosis.

It differs from *Japonia barbata* (L. Pfeiffer, 1855) by having a smaller shell (shell height: 5.84–6.20 mm vs. 6.25–9.25 mm; shell width: 8.0–8.88 mm vs. 9.5–15.8 mm). In addition, it has very short feather-like periostracal hairs along the first peripheral ridge, while *J. barbata* has five-times longer feather-like periostracal hairs.

####### Description.

Shell small, depressed-conical, dextral, rather solid. Colour brownish to yellowish white, translucent, shiny with or without prominent brown radiating markings. Suture impressed. Whorls 5¼, convex, regularly increasing in diameter. Periphery rounded, slightly angular at ultimate whorl. Protoconch: smooth, dark brown, more or less rounded without spiral striae. Teleoconch with radial sculpture consisting of very fine transverse growth lines all over the shell. Spiral sculpture with seven spiral ridges, three above periphery, two along periphery, one below periphery, and one near base. Spiral striae absent. Aperture almost circular, somewhat oblique, parietal area between two spiral ridges below periphery and near base. Peristomes double, with prominent outer peristome except for the supra-columellar site, with a distinct notch near suture, inner peristome slightly expanded. Periostracum thin, corneous, and smooth with very short, deciduous, slender, feather-like hairs along first peripheral ridge and below periphery in fresh condition. Umbilicus: open, moderately wide, 1.80–2.00 mm in diameter. Dimensions: Shell height 5.84–6.20 mm; shell width 8.0–8.88 mm; Aperture height and width 2.60 mm.

####### Remarks.

Shells of some populations (i.e., Gunung Kapor areas) without or with inconspicuous spiral lirae (Fig. 4C).

####### Geographic distribution and habitat.

Bau and Serian-Padawan limestone hill clusters. Living snails were observed foraging among leaf litter and plant debris near the cliff in lowland limestone forest.

####### Etymology.

For Bau District, where the specimens were found.

**Figure 5.**
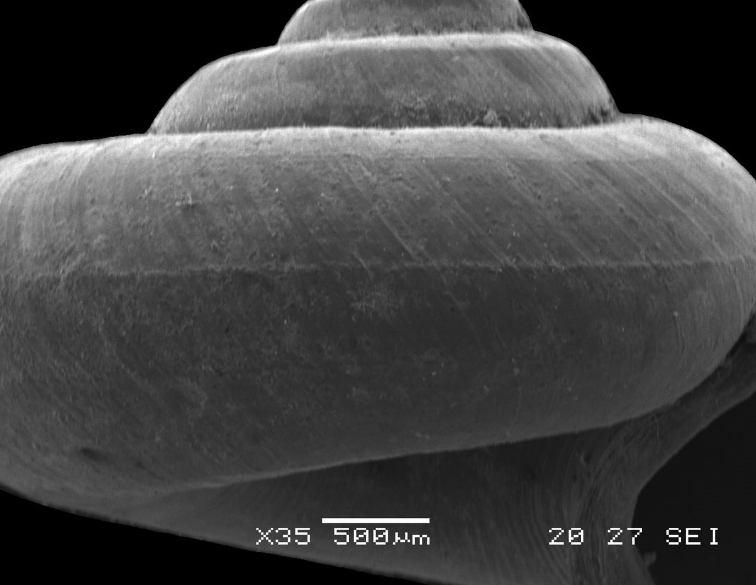
*Japonia bauensis* sp. nov., ME 7231 paratype. Enlargement of the body whorl showing the shell sculpture.

###### Japonia metcalfei

Taxon classificationAnimaliaArchitaenioglossaCyclophoridae

(Issel, 1874)

2D57A225-6761-520B-A6C2-32E60B18E7B5

Figure 6A

Cyclophorus (Craspedotropis) metcalfei Issel, 1874: 432–433, pl. 6, figs 4–6.

####### Type locality.

“Territorio di Sarawak”.

####### Material examined.

Gunung Doya: ME 9161. Lobang Angin: ME 9279. Gunung Batu: ME 2916.

####### Distribution in Borneo.

Sarawak: Kuching Division. Endemic to Borneo.

####### Remarks.

Only dry shells were found during the surveys.

**Figure 6.**
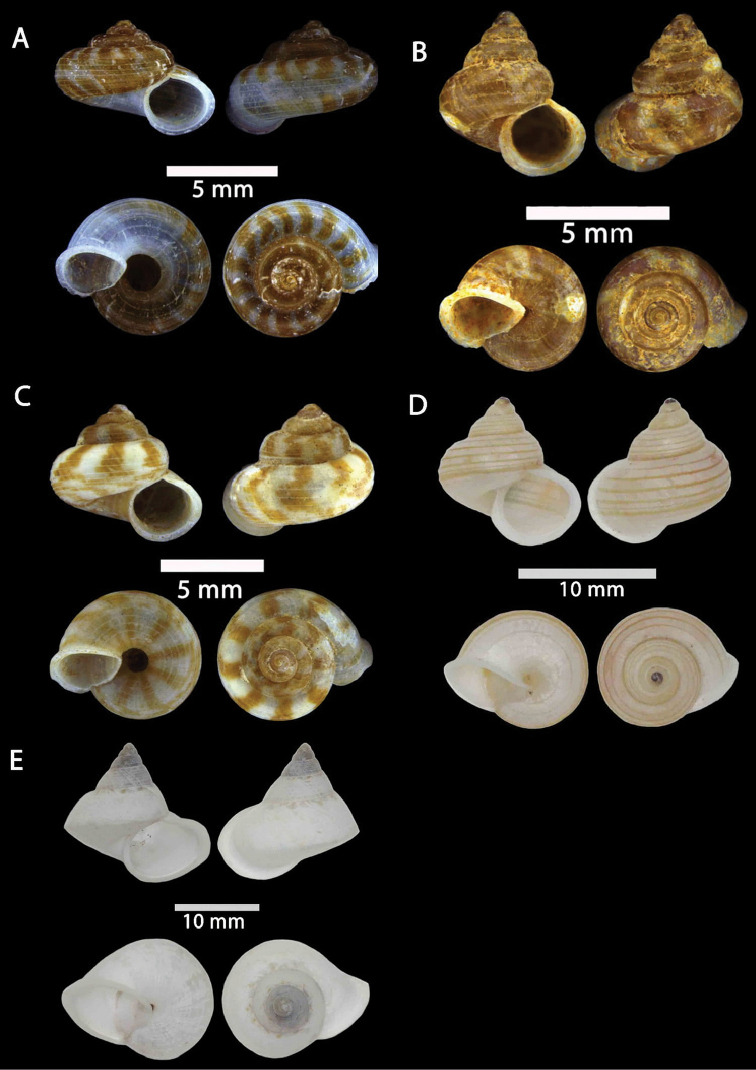
**A***Japonia metcalfei* (Issel, 1874) ME 2916 Gunung Batu **B***Japonia mundyana* (Godwin-Austen, 1889) ME 0838 Gunung Batu **C***Japonia rabongensis* (E. A. Smith, 1895) ME 9080 Gunung Kapor **D***Leptopoma sericatum* (L. Pfeiffer, 1851) ME 5900 Gunung Stulang **E***Leptopoma undatum* (Metcalfe, 1851) ME 2684 Gunung Batu.

###### Japonia mundyana

Taxon classificationAnimaliaArchitaenioglossaCyclophoridae

(Godwin-Austen, 1889)

7E2E79B3-9D2A-5F13-A10E-3D26112B1619

Figure 6B

Lagocheilus mundyanus Godwin-Austen, 1889: 338–339, pl. 39, figs 6, 6A, B.

####### Type locality.

“Busan Hills, Borneo” [= Jambusan Hills, Bau, Sarawak].

####### Material examined.

Gunung Kapor: ME 0821, ME 8493, ME 9249. Gunung Batu: ME 0808, ME 0838.

####### Distribution in Borneo.

Sarawak: Kuching Division. Endemic to Borneo.

####### Remarks.

It differs from other the Bornean *Japonia* species by having a considerably smaller shell with higher spire. Only dry shells were found during the surveys.

###### Japonia rabongensis

Taxon classificationAnimaliaArchitaenioglossaCyclophoridae

(E. A. Smith, 1895)

0E2FC2D8-A354-5B66-995B-09C6841D21D8

Figure 6C

Lagochilus rabongensis E. A. Smith, 1895: 120–121, pl. 4, fig. 6.

####### Type locality.

“Mount Rabong, West Sarawak”.

####### Material examined.

Gunung Kapor: ME 8492, ME 9080. Gunung Batu: ME 0824.

####### Distribution in Borneo.

Sarawak: Kuching Division. Endemic to Borneo.

####### Remarks.

It is similar to *Japonia metcalfei* (Issel, 1874), but differs in having higher spire and narrower umbilicus. Only dry shells were found during the surveys.

##### *Leptopoma* L. Pfeiffer, 1847

###### Leptopoma sericatum

Taxon classificationAnimaliaArchitaenioglossaCyclophoridae

(L. Pfeiffer, 1851)

647EC4F7-FB38-5327-9CFB-B32E0E88818D

Figure 6D

Cyclostoma (Leptopoma) sericatum L. Pfeiffer, 1851: 244.

####### Type locality.

“Borneo”.

####### Material examined.

Bukit Sekunyit: ME 1350. Gunung Doya: ME 1355, ME 8906, ME 9155. Gunung Kapor: ME 1290, ME 1347, ME 1352, ME 8070, ME 9232, ME 9403. Gunung Stulang: ME 5900. Gunung Batu: ME 1351, ME 1354, ME 1359, ME 8831.

####### Distribution in Borneo.

Sarawak: Kuching, Serian, Kapit and Miri division. Sabah, Brunei Darussalam, Kalimantan: West, South, and East Kalimantan provinces. Endemic to Borneo.

###### Leptopoma undatum

Taxon classificationAnimaliaArchitaenioglossaCyclophoridae

(Metcalfe, 1851)

E23931E0-9BC0-5844-B1C7-586DDB21953A

Figure 6E

Cyclostoma undatum Metcalfe, 1851: 71.

####### Type locality.

“Borneo”.

####### Material examined.

Gunung Stulang: ME 5899. Kampung Padang Pan: ME 6723. Gunung Batu: ME 2684, ME 2686.

####### Distribution in Borneo.

Sarawak: Kuching and Miri divisions. Sabah: Interior, Sandakan, Tawau, and West Coast divisions. Brunei Darussalam: Temburong District. Kalimantan: West and East Kalimantan provinces. ***Distribution elsewhere.*** Palawan (Vermeulen 1999).

##### *Opisthoporus* Benson, 1851

###### Opisthoporus biciliatus

Taxon classificationAnimaliaArchitaenioglossaCyclophoridae

(Mousson, 1849)

85F39FE1-9165-562B-B6D0-89D9935B760C

Figures 7A , 46C

Pterocyclos biciliatum Mousson, 1849: 49–50, pl. 20, fig. 9.

####### Type locality.

“Java” [= Borneo (Metcalfe, 1851)].

####### Material examined.

Gunung Doya: ME 4744, ME 9144, ME 9192. Gunung Kapor: ME 3723, ME 4742, ME 4746, ME 5973, ME 8073, ME 8460, ME 8754, ME 8757, ME 8777. Lobang Angin: ME 4743, ME 8725, ME 8729, ME 8739, ME 9484. Gunung Batu: ME 4745, ME 4748, ME 8806.

####### Distribution in Borneo.

Sarawak: Kuching, Samarahan, Serian, and Sibu divisions. Kalimantan: West Kalimantan Province. Endemic to Borneo.

**Figure 7.**
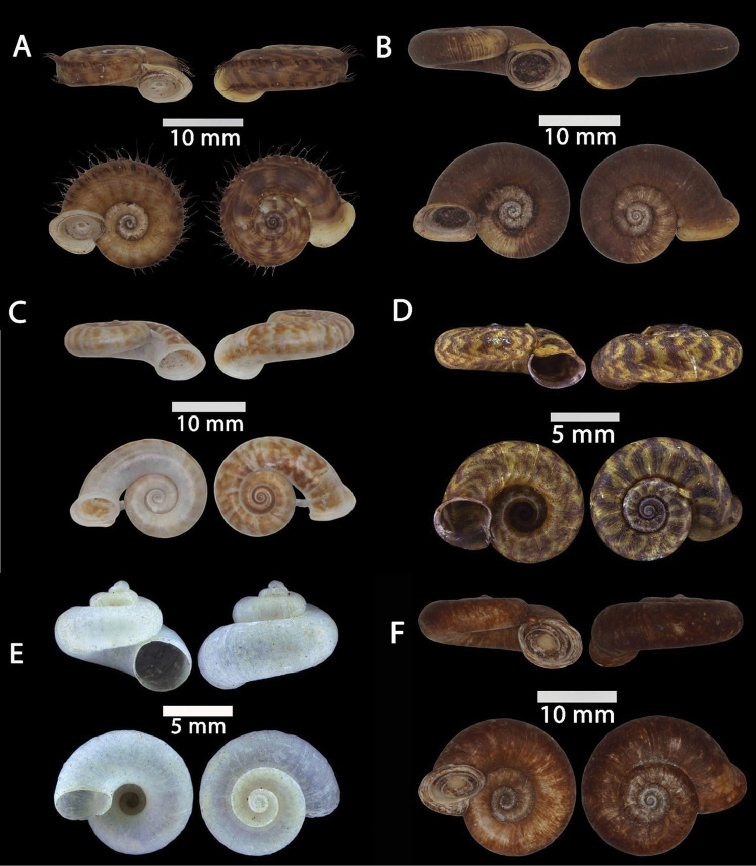
**A***Opisthophorus biciliatus* (Mousson, 1849) ME 8754 Gunung Kapor **B***Opisthophorus birostris* (L. Pfeiffer, 1854) ME 8758 Gunung Kapor **C***Opisthophorus cavernae* (Godwin-Austen, 1889) ME 8904 Gunung Doya **D***Opisthophorus euryomphalus* (L. Pfeiffer, 1856) ME 8779 Gunung Kapor **E***Platyrhaphe linita* (Godwin-Austen, 1889) ME 0881 Gunung Batu **F***Pterocyclos tenuilabiatus* (Metcalfe, 1851) ME 5910 Gunung Batu.

####### Remarks.

Living snails were observed foraging among leaf litter and plant debris near the cliff in lowland limestone forest. It differs from other Bornean *Opisthoporus* species by having a moderately large shell, short sutural tube projecting upwards, and long, double, deciduous appendages along the first peripheral ridge and below the periphery that can be seen on a fresh shell.

###### Opisthoporus birostris

Taxon classificationAnimaliaArchitaenioglossaCyclophoridae

(L. Pfeiffer, 1854)

B4A99D04-E014-51DF-9A9F-1764CE95B72B

Figures 7B , 46D

Cyclostoma birostre Pfeiffer, 1854b: 300.

####### Type locality.

“Sarawak, Borneo”.

####### Material examined.

Bukit Sekunyit: ME 4735. Gunung Doya: ME 1312. Gunung Kapor: ME 4731, ME 4736, ME 4737, ME 8072, ME 8495, ME 8755, ME 8758, ME 9605. Lobang Angin: ME 4738. Gunung Batu: ME 4732, ME 4734, ME 4739, ME 8830.

####### Distribution in Borneo.

Sarawak: Kuching Division. Endemic to Borneo.

####### Remarks.

Living snails were observed foraging among leaf litter and plant debris near the cliff in lowland limestone forest. It differs from other Bornean *Opisthoporus* species by having a large shell, short sutural tube projecting downwards, and a thick brown periostracum can be seen on fresh shells.

###### Opisthoporus cavernae

Taxon classificationAnimaliaArchitaenioglossaCyclophoridae

(Godwin-Austen, 1889)

A0F31125-9243-524D-A0BB-85CEBC2013E2

Figure 7C

Rhiostoma cavernae Godwin-Austen, 1889: 342, pl. 36, figs 1, 1A.

####### Type locality.

“Sarawak proper, Borneo” [= Kuching, Sarawak].

####### Material examined.

Gunung Doya: ME 8904, ME 9093.

####### Distribution in Borneo.

Sarawak: Kuching and Serian divisions. Endemic to Borneo.

####### Remarks.

Living snails were observed foraging among leaf litter and plant debris near the cliff in lowland limestone forest. It differs other Bornean *Opisthoporus* species by having a moderate shell, short sutural tube projecting upward, long *Rhiostoma*-like detached tuba.

###### Opisthoporus euryomphalus

Taxon classificationAnimaliaArchitaenioglossaCyclophoridae

(L. Pfeiffer, 1856)

4D33DFF0-6084-5159-8ED1-30C33EBCE196

Figures 7D , 46B

Cyclostoma (Opisthoporus) euryomphalum Pfeiffer, 1856: 337.

####### Type locality.

“Borneo”.

####### Material examined.

Gunung Doya: ME 1295, ME 8905, ME 9113, ME 9157. Gunung Kapor: ME 1294, ME 1298, ME 1299, ME 8071, ME 8496, ME 8779. Gunung Batu: ME 1300, ME 1301, ME 2613, ME 8807.

####### Distribution in Borneo.

Sarawak: Kuching Division. Kalimantan: West Kalimantan Province. Endemic to Borneo.

####### Remarks.

Living snails were observed foraging among leaf litter and plant debris near the cliff in a lowland limestone forest. It differs from *O. biciliatus* by having a smaller shell without the hairy periostracum.

##### *Platyrhaphe* Möllendorff, 1890

###### Platyrhaphe linita

Taxon classificationAnimaliaArchitaenioglossaCyclophoridae

(Godwin-Austen, 1889)

1EF40A3B-9DFF-5886-AF4B-30DEA95A6CB4

Figures 7E , 47B

Cyclotus linitus Godwin-Austen, 1889: 345, pl. 36, fig. 3.

####### Type locality.

“Busan Hills, Borneo” [= Jambusan Hills, Bau, Sarawak].

####### Material examined.

Gunung Doya: ME 9700, ME 8908, ME 9094, ME 9175. Gunung Batu: ME 0881, ME 0882.

####### Distribution in Borneo.

Sarawak: Kuching and Serian divisions. Endemic to Borneo.

####### Remarks.

This species is one of only four species of *Platyrhaphe* described from Borneo and the only one recorded from Sarawak. Living snails were observed foraging among leaf litter and plant debris near the cliff in lowland limestone forest. The shell is always covered with thick dirt.

##### *Pterocyclos* Benson, 1832

###### Pterocyclos tenuilabiatus

Taxon classificationAnimaliaArchitaenioglossaCyclophoridae

(Metcalfe, 1851)

B7303B33-9324-5AAE-8A9B-C8016963EA13

Figure 7F

Cyclostoma tenuilabiatum Metcalfe, 1851: 71–72.

####### Type locality.

“Borneo”.

####### Material examined.

Gunung Kapor: ME 8074, ME 9467. Gunung Stulang: ME 5901.

####### Distribution in Borneo.

Sabah: West Coast, Interior, Sandakan, and Tawau divisions. SARAWAK: Kuching Division. Kalimantan: West Kalimantan Province. Endemic to Borneo.

####### Remarks.

Living snails were observed foraging among leaf litter and plant debris near the cliff in a lowland limestone forest.

#### Family Diplommatinidae L. Pfeiffer, 1856

##### *Diplommatina* Benson, 1849

###### Diplommatina adversa

Taxon classificationAnimaliaArchitaenioglossaDiplommatinidae

(H. Adams & A. Adams, 1851)

672C93A4-132F-50DC-AEBF-70CF5C9C188C

Figures 8A , 49A

Paxillus adversus H. Adams & A. Adams, 1851: 63.

####### Type locality.

Singapore.

####### Material examined.

Bukit Sekunyit: ME 0462. Gunung Doya: ME 0543, ME 9695, ME 8898, ME 9089. Gunung Kapor: ME 0455, ME 0456, ME 0523, ME 0525, ME 8067, ME 8513, ME 8760, ME 8766, ME 8776. Kampung Padang Pan: ME 6672. Lobang Angin: ME 0463, ME 0524, ME 8735, ME 8742, ME 8748. Gunung Batu: ME 0459, ME 0526, ME 0535, ME 8799.

####### Distribution in Borneo.

Sarawak: Kuching and Serian divisions. Sabah: Tawau Division. ***Distribution elsewhere.*** West Malaysia, Singapore, Bunguran (Indonesia) and Sirhassen (Indonesia) (Laidlaw 1949).

####### Remarks.

Living snails were observed foraging among leaf litter and plant debris near the cliff in a lowland limestone forest.

**Figure 8.**
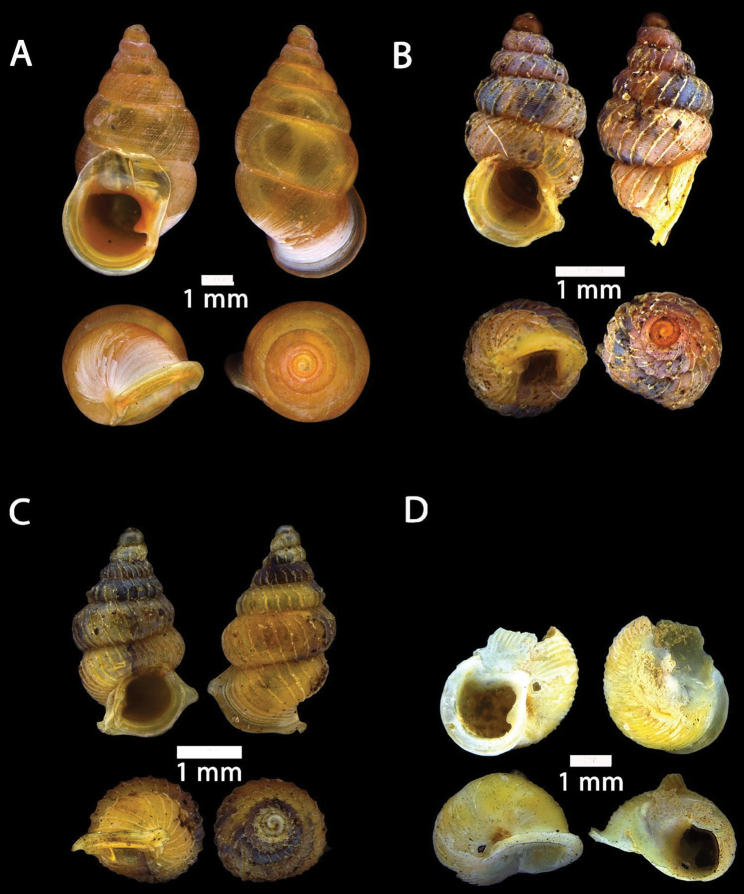
**A***Diplommatina adversa* (H. Adams & A. Adams, 1851) ME 8799 Gunung Batu **B***Diplommatina busanensis* Godwin-Austen, 1889 ME 8991 Gunung Doya **C***Diplommatina concinna* H. Adams, 1872 ME 8745 Lobang Angin **D***Diplommatina isseli* Godwin-Austen, 1889 ME 2869 Gunung Batu.

###### Diplommatina busanensis

Taxon classificationAnimaliaArchitaenioglossaDiplommatinidae

Godwin-Austen, 1889

4115B9B6-A792-534F-8702-1750018DF93D

Figure 8B

Diplommatina busanensis Godwin-Austen, 1889: 348–349, pl. 37, fig. 4.

####### Type locality.

“Busan Hills, Borneo” [= Jambusan Hills, Bau, Sarawak].

####### Material examined.

Gunung Doya: ME 8902, ME 8991, ME 9088. Gunung Kapor: ME 0307, ME 0483, ME 0600, ME 8485, ME 9233, ME 9260, ME 9278. Gunung Batu: ME 0467, ME 8800.

####### Distribution in Borneo.

Sarawak: Kuching Division. Endemic to Borneo.

####### Remarks.

Living snails were observed foraging among leaf litter and plant debris near the cliff in a lowland limestone forest.

###### Diplommatina concinna

Taxon classificationAnimaliaArchitaenioglossaDiplommatinidae

H. Adams, 1872

41D3366A-DE94-5F18-83BC-B5DDFDDF0088

Figures 8C , 49B

Diplommatina concinna H. Adams, 1872: 13, pl. 3, fig. 22.

####### Type locality.

“Borneo”.

####### Material examined.

Gunung Sebayat: ME 8003. Bukit Sekunyit: ME 0464. Gunung Doya: ME 0544, ME 9696, ME 8992, ME 9087, ME 9101. Gunung Kapor: ME 0466, ME 0491, ME 0517, ME 0599, ME 8147, ME 8484, ME 9005, ME 9137, ME 9230. Gunung Stulang: ME 5902. Kampung Bunga Rampai: ME 0736. Kampung Padang Pan: ME 6671. Lobang Angin: ME 0515, ME 8745, ME 8981, ME 9024. Gunung Batu: ME 0512, ME 0538, ME 0540, ME 0597, ME 8797.

####### Distribution in Borneo.

Sarawak: Kuching, Serian, and Miri divisions. ***Distribution elsewhere.*** Bunguran (Indonesia) (Smith 1874).

####### Remarks.

Living snails were observed foraging among leaf litter and plant debris near the cliff in a lowland limestone forest.

###### Diplommatina isseli

Taxon classificationAnimaliaArchitaenioglossaDiplommatinidae

Godwin-Austen, 1889

23135A60-E827-58C6-9810-C36C0583DF21

Figure 8D

Diplommatina isseli Godwin-Austen, 1889: 348, pl. 38, figs 5, 5A.

####### Type locality.

“Sarawak proper and Busan Hills, Borneo” [= Jambusan Hills, Bau, Sarawak].

####### Material examined.

Gunung Batu: ME 2869.

####### Distribution in Borneo.

Sarawak: Kuching Division. Sabah: Interior and West Coast divisions. Endemic to Borneo.

####### Remarks.

Only dry shells were found during the surveys.

###### Diplommatina maduana maduana

Taxon classificationAnimaliaArchitaenioglossaDiplommatinidae

Laidlaw, 1949

5BA1CF4B-2AAC-5AD6-A7EF-0C08BA82BE65

Figure 9A

Diplommatina maduana Laidlaw, 1949: 209, fig. 3B.

####### Type locality.

“Gua Madu, Kelantan”.

####### Material examined.

Gunung Sebayat: ME 8004. Gunung Doya: ME 0542, ME 9030, ME 9086, ME 9162. Gunung Kapor: ME 0475, ME 0532, ME 0598, ME 8486, ME 9004, ME 9028, ME 9076. Gunung Batu: ME 0534, ME 8798.

####### Distribution in Borneo.

Sarawak: Kuching, Serian and Miri divisions. ***Distribution elsewhere.*** West Malaysia (Laidlaw 1949).

####### Remarks.

Living snails were observed foraging among leaf litter and plant debris near the cliff in a lowland limestone forest.

**Figure 9.**
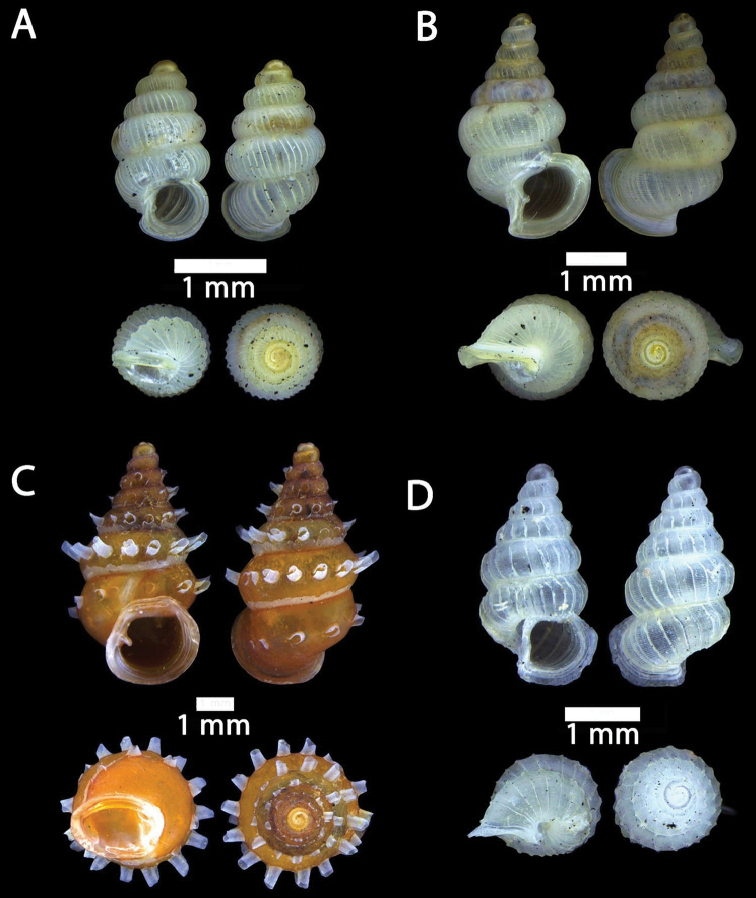
**A***Diplommatina maduana maduana* Laidlaw, 1949 ME 9030 Gunung Doya **B***Diplommatina onyx* Fulton, 1901 ME 9102 Gunung Doya **C***Diplommatina spinosa* Godwin-Austen, 1889 ME 8801 Gunung Batu **D***Diplommatina toretos* Vermeulen, 1993 ME 0536 Gunung Batu.

###### Diplommatina onyx

Taxon classificationAnimaliaArchitaenioglossaDiplommatinidae

Fulton, 1901

33C29F56-5A2C-58B2-B7E1-256CB229681B

Figure 9B

Diplommatina onyx Fulton, 1901: 244.

####### Type locality.

“Busan, N. Borneo” [= Jambusan Hills, Sarawak].

####### Material examined.

Gunung Doya: ME 0541, ME 9014, ME 9102, ME 9150. Gunung Kapor: ME 8487. Gunung Batu: ME 0484, ME 0537, ME 8888.

####### Distribution in Borneo.

Sarawak: Kuching, Serian, and Miri divisions. Endemic to Borneo.

####### Remarks.

Living snails were observed foraging among leaf litter and plant debris near the cliff in a lowland limestone forest.

###### Diplommatina spinosa

Taxon classificationAnimaliaArchitaenioglossaDiplommatinidae

Godwin-Austen, 1889

661EA70F-ACD2-5870-9744-0B409F4BB9A3

Figures 9C , 49C

Diplommatina spinosa Godwin-Austen, 1889: 349, pl. 38, fig. 1.

####### Type locality.

“Cave exploration A, Borneo” [= Tupak Cave, Jambusan Hills (Cranbrook, 2013)].

####### Material examined.

Gunung Batu: ME 0522, ME 0539, ME 0596, ME 8801.

####### Distribution in Borneo.

Sarawak: Kuching and Serian divisions. Endemic to Borneo.

####### Remarks.

Living snails were observed foraging among leaf litter and plant debris near the cliff in a lowland limestone forest.

###### Diplommatina toretos

Taxon classificationAnimaliaArchitaenioglossaDiplommatinidae

Vermeulen, 1993

A68C2F21-2434-5D8D-8F47-4A9A4CE5DD55

Figure 9D

Diplommatina toretos Vermeulen, 1993: 19–20, fig. 13A–D.

####### Type locality.

“SARAWAK. 1^st^ Div.: G. Pangga 3 km ENE of Bau”.

####### Material examined.

Gunung Doya: ME 0545. Gunung Batu: ME 0536.

####### Distribution in Borneo.

Sarawak: Kuching, Serian and Miri divisions. Endemic to Borneo.

####### Remarks.

Only dry shells were found during the surveys.

##### *Opisthostoma* W. T. Blanford & H. F. Blanford, 1860

###### Opisthostoma ballorum

Taxon classificationAnimaliaArchitaenioglossaDiplommatinidae

Vermeulen, 1991

6D67E733-FD1C-5875-A3BB-AE472B9A838E

Figure 10A

Opisthostoma ballorum Vermeulen, 1991a: 162–163, fig. 10b.

####### Type locality.

“SARAWAK. 1^st^ Div.: G. Kapur 6 km SE of Bau”.

####### Material examined.

Gunung Doya: ME 8989, ME 9109, ME 9149. Gunung Kapor: ME 0274, ME 0277, ME 9002, ME 9206. Lobang Angin: ME 9171. Gunung Batu: ME 0273, ME 0276, ME 0331, ME 8796.

####### Distribution in Borneo.

Sarawak: Kuching Division. Endemic to Borneo.

####### Remarks.

Living snails were observed foraging among leaf litter and plant debris near the cliff in a lowland limestone forest.

**Figure 10.**
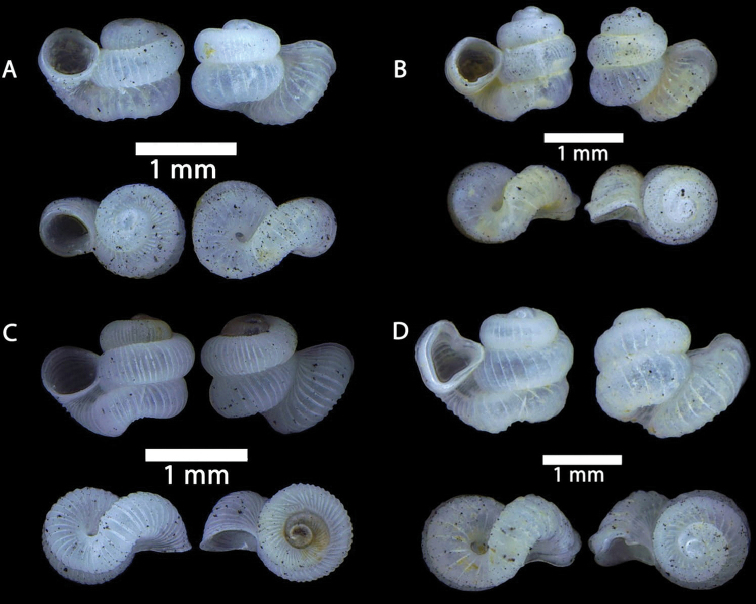
**A***Opisthostoma ballorum* Vermeulen, 1991 ME 9002 Gunung Kapor **B***Opisthostoma brachyacrum brachyacrum* Thompson, 1978 ME 0353 Gunung Batu **C***Opisthostoma brachyacrum lambi* (Vermeulen, 1991) ME 9003 Gunung Kapor **D***Opisthostoma cryptodon* Vermeulen, 1991 ME 9084 Gunung Doya.

###### Opisthostoma brachyacrum brachyacrum

Taxon classificationAnimaliaArchitaenioglossaDiplommatinidae

Thompson, 1978

6879E1AB-A543-58CF-8DC3-9A5226A438A1

Figure 10B

Opisthostoma (Opisthostoma) brachyacrum Thompson, 1978: 388–389, figs 2A–E.

####### Type locality.

“BORNEO: Sarawak. Fourth Division. Limestone hill on the trail from the Niah River to Niah Cave, Batu Niah”.

####### Material examined.

Gunung Kapor: ME 0285. Gunung Batu: ME 0279, ME 0284, ME 0353.

####### Distribution in Borneo.

Sarawak: Kuching and Miri divisions. Endemic to Borneo.

####### Remarks.

Living snails were observed foraging among leaf litter and plant debris near the cliff in a lowland limestone forest.

###### Opisthostoma brachyacrum lambi

Taxon classificationAnimaliaArchitaenioglossaDiplommatinidae

(Vermeulen, 1991)

39270A4F-6E2A-5695-84C8-B687C3AD15AC

Figure 10C

Opisthostoma lambii Vermeulen, 1991a: 155.

####### Type locality.

“SARAWAK. 1^st^ Div.: W of Kpg. Lobang Batu 12.5 km S of Tebakang”.

####### Material examined.

Gunung Sebayat: ME 8002. Bukit Sekunyit: ME 0311. Gunung Doya: ME 0287, ME 9082, ME 9246. Gunung Kapor: ME 0281, ME 0310, ME 2875, ME 8978, ME 9003, ME 9077. Kampung Padang Pan: ME 6670. Lobang Angin: ME 9106, ME 9147, ME 9224.

####### Distribution in Borneo.

Sarawak: Kuching, Serian and Kapit divisions. Sabah: Interior and West Coast divisions. Endemic to Borneo.

####### Remarks.

Living snails were observed foraging among leaf litter and plant debris near the cliff in a lowland limestone forest.

###### Opisthostoma cryptodon

Taxon classificationAnimaliaArchitaenioglossaDiplommatinidae

Vermeulen, 1991

C81B2CB9-1A65-57F5-8A17-46DE8D188BC1

Figure 10D

Opisthostoma cryptodon Vermeulen, 1991: 148–150, fig. 4B.

####### Type locality.

“SARAWAK. 1^st^ Div.: W of Kpg. Lobang Batu 12.5 km S of Tebakang”.

####### Material examined.

Gunung Doya: ME 8901, ME 8988, ME 9084. Gunung Kapor: ME 0278, ME 9099, ME 9253. Lobang Angin: ME 8980, ME 9141. Gunung Batu: ME 0329.

####### Distribution in Borneo.

Sarawak: Kuching and Serian divisions. Endemic to Borneo.

####### Remarks.

Living snails were observed foraging among leaf litter and plant debris near the cliff in lowland limestone forest.

###### Opisthostoma planiapex

Taxon classificationAnimaliaArchitaenioglossaDiplommatinidae

Vermeulen, 1991

8AC8846C-5079-55D4-83BE-29EA247DCCC0

Figure 11A

Opisthostoma planiapex Vermeulen, 1991: 145–147, fig. 3C.

####### Type locality.

“SARAWAK. 1^st^ Div.: G. Kapur 6 km SE of Bau”.

####### Material examined.

Gunung Kapor: ME 0263, ME 0352, ME 8483, ME 9205, ME 9250, ME 9267.

####### Distribution in Borneo.

Sarawak: Kuching Division. Endemic to Borneo.

####### Remarks.

Living snails were observed foraging among leaf litter and plant debris near the cliff in a lowland limestone forest.

**Figure 11.**
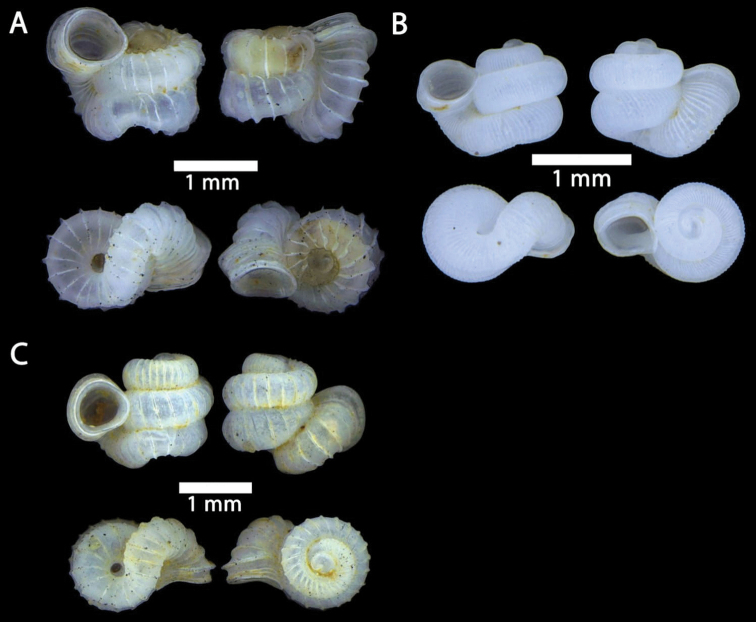
**A***Opisthostoma planiapex* Vermeulen, 1991 ME 9250 Gunung Kapor **B***Opisthostoma simile* Vermeulen, 1991 ME 11471 Gunung Kapor **C***Opisthostoma tridens* Vermeulen, 1991 ME 0333 Gunung Kapor.

###### Opisthostoma simile

Taxon classificationAnimaliaArchitaenioglossaDiplommatinidae

Vermeulen, 1994

A594B454-500D-5CFE-A419-71A73ABC7156

Figure 11B

Opisthostoma (Opisthostoma) simile Vermeulen, 1994: 96, figs 15A, B, 67.

####### Type locality.

“SARAWAK. 1^st^ Div.: G. Lelat 1 mile SW of Nyabet, 24 miles SSE of Kuching”.

####### Material examined.

Gunung Kapor: ME 9598, ME 11471, ME 11478.

####### Distribution in Borneo.

Sarawak: Kuching Division. Endemic to Borneo.

####### Remarks.

Only dry shells were found during the surveys.

###### Opisthostoma tridens

Taxon classificationAnimaliaArchitaenioglossaDiplommatinidae

Vermeulen, 1991

CBA67E1F-2086-5599-AC4C-3925387E4573

Figure 11C

Opisthostoma tridens Vermeulen, 1991: 152, fig. 5C, D.

####### Type locality.

“SARAWAK. 1^st^ Div.: Kpg. Beratok along road Kuching-Serian”.

####### Material examined.

Gunung Kapor: ME 0333.

####### Distribution in Borneo.

Sarawak: Kuching and Serian divisions. Endemic to Borneo.

####### Remarks.

Only dry shells were found during the surveys.

##### *Plectostoma* H. Adams, 1865

###### Plectostoma austeni

Taxon classificationAnimaliaArchitaenioglossaDiplommatinidae

(E. A. Smith, 1894)

2BC99DAF-7D72-517A-B176-5F9AF231D98A

Figures 12A , 48C

Opisthostoma austeni E. A. Smith, 1894a: 272–273.

####### Type locality.

“Rumbang, Sarawak”.

####### Material examined.

Gunung Doya: ME 0248, ME 9013, ME 9244. Gunung Kapor: ME 0255. Lobang Angin: ME 0250, ME 8734, ME 8744, ME 9180. Gunung Batu: ME 0249, ME 0253, ME 0259, ME 8794.

####### Distribution in Borneo.

Sarawak: Kuching and Serian divisions. Endemic to Borneo.

####### Remarks.

Living snails were observed foraging inside the rock crevices and cave walls, away from direct exposure to light. A single sinistral shell was found within the normal dextral populations.

**Figure 12.**
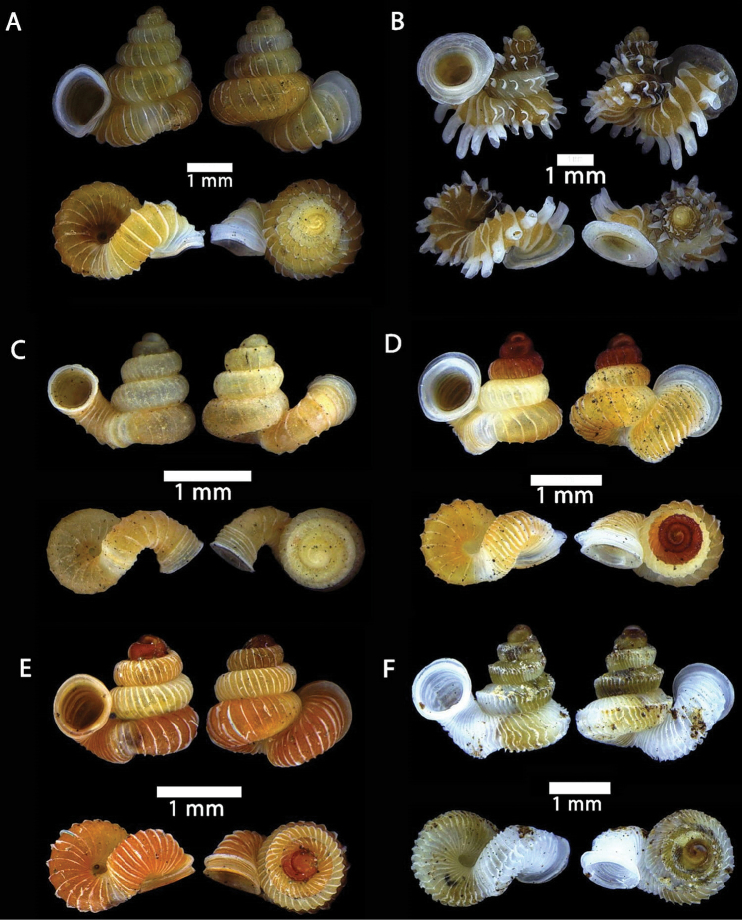
**A***Plectostoma austeni* (E. A. Smith, 1894) ME 8734 Lobang Angin **B***Plectostoma everetti* (E. A. Smith, 1893) ME 8793 Gunung Batu **C***Plectostoma margaretchanae*, sp. nov., MZU.MOL.20.06 paratype Gunung Batu **D***Plectostoma wallacei busauense* (E. A. Smith, 1893) ME 9495 Gunung Doya **E***Plectostoma wallacei teinostoma* (Vermeulen, 1994) ME 8899 Gunung Doya **F***Plectostoma wallacei wallacei* Ancey, 1887 ME 5898 Gunung Stulang.

###### Plectostoma everetti

Taxon classificationAnimaliaArchitaenioglossaDiplommatinidae

(E. A. Smith, 1893)

E531BC26-4A45-51F7-93AE-C440B3ECAD8B

Figures 12B , 48D

Opisthostoma everetti E. A. Smith, 1893: 346–347, pl. 25, figs 12, 12A.

####### Type locality.

“Jambusan, N.W. Borneo” [= Jambusan Hills, Bau, Sarawak].

####### Material examined.

Gunung Doya: ME 8900, ME 9012, ME 9158. Gunung Batu: ME 0219, ME 0222, ME 2834, ME 8793.

####### Distribution in Borneo.

Sarawak: Kuching Division. Endemic to Borneo.

####### Remarks.

Living snails were observed foraging on the moderately wet vertical limestone rock surfaces covered with mosses and lichens.

###### Plectostoma margaretchanae sp. nov.

Taxon classificationAnimaliaArchitaenioglossaDiplommatinidae

5E3AE750-50B2-54DB-B617-ECD13F2632E9

http://zoobank.org/EBE82C79-E745-439F-B353-419D1D2D1B0B

Figures 12C , 13A–F

####### Material examined.

***Holotype*** (SH 1.35 mm, SW 1.80 mm) (MZU.MOL.20.04), Malaysia, Sarawak, Kuching Division, Gunung Batu, limestone hill along Skio road, Jambusan, 2.4 miles E Bau, 1°23'50.65"N, 110°11'19.99"E, coll. M. E. Marzuki, 10.VII.2011. ***Paratypes***: > 10 ex. (ME0000227), same data as holotype; 2 ex. (MZU.MOL.20.05), > 10 ex. (ME0000217), the same locality as holotype, coll. M. E. Marzuki, 11.III.2011.

####### Differential diagnosis.

It differs other Bornean *Plectostoma* species by having a tiny shell, long projecting tuba free from the spire, and a constriction with a transverse palatalis and transverse basalis. It is most similar to *P. pyrgiscus* (Vermeulen, 1994) and *P. tuba* (Vermeulen, 1994) but *P. pyrgiscus* has a higher spire of six whorls with widely spaced radial ribs, while *P. tuba* also has higher spire without spiral striation, but with a double peristome.

####### Description.

Shell spire conical with slightly convex sides. Apex not or slightly oblique. Whorls 4½, convex; last whorl rounded. Constriction with a transverse palatalis, and a transverse basalis. Tuba free from the spire, long projecting, abruptly narrowed towards the constriction, rounded below. Teleoconch: radial ribs on spire moderately spaced (six ribs/0.5 mm on the penultimate whorl), not sinuous, those close to tuba not sinuous; those on tuba widely spaced (three ribs/0.5 mm half-way), not or hardly sinuous below. Spiral striation present, distinct. Aperture hardly tilted with regards to coiling axis, circular to elliptical, peristome simple, distant from the spire; slightly spreading. Umbilicus open, 0.13 mm across. Dimensions: spire height 1.25–1.35 mm; spire width 1.00 mm, shell width (including tube) 1.60–1.80 mm; aperture height and width 0.47 mm.

####### Geographic distribution and habitat.

Only known from the type locality. Living snails were observed foraging on the wet vertical limestone rock surfaces covered with mosses and lichens.

####### Etymology.

The specific epithet honours the Agronomist, Margaret Chan Kit Yok of Universiti Teknologi MARA, who was the mentor for the first author by providing valuable guidance for his malacological research in Sarawak.

**Figure 13.**
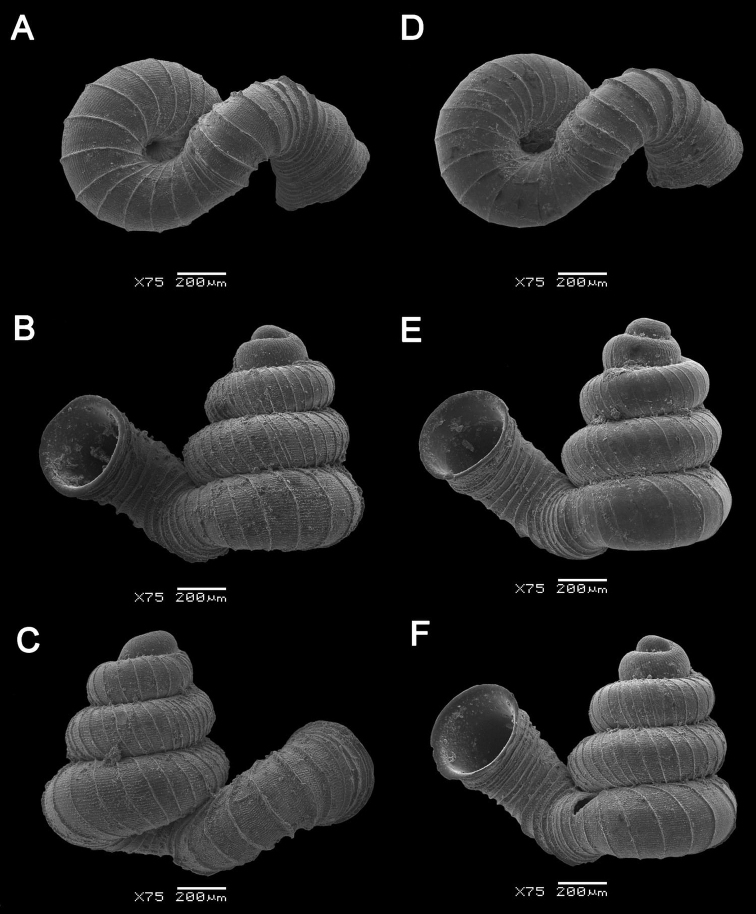
*Plectostoma margaretchanae* sp. nov. **A–C**MZU.MOL.20.04 holotype **A** Basal view **B** Apertural view **C** Abapertural view **D–F** ME 0227, paratypes **D** Basal view **E–F** Apertural view.

###### Plectostoma wallacei busauense

Taxon classificationAnimaliaArchitaenioglossaDiplommatinidae

(E. A. Smith, 1893)

88965090-2E35-539D-A147-3AC6372ABA28

Figure 12D

Opisthostoma busauense E. A. Smith, 1893: 348, pl. 25, figs 16, 16A.

####### Type locality.

“Busau, N.W. Borneo” [= Jambusan Hills, Bau, Sarawak].

####### Material examined.

Bukit Sekunyit: ME 2145. Gunung Doya: ME 9495.

####### Distribution in Borneo.

SARAWAK: Kuching Division. Endemic to Borneo.

####### Remarks.

Living snails were observed foraging on the wet vertical limestone rock surfaces covered with mosses and lichens.

###### Plectostoma wallacei teinostoma

Taxon classificationAnimaliaArchitaenioglossaDiplommatinidae

(Vermeulen, 1994)

517707B0-AFB3-5739-BD04-D36914EC9B7E

Figure 12E

Opisthostoma (Plectostoma) wallacei teinostoma Vermeulen, 1994: 129, figs 59A, B, 71.

####### Type locality.

“SARAWAK. 1^st^ Div.: G. Pangga 3 km ENE of Bau”.

####### Material examined.

Gunung Doya: ME 0716, ME 8899, ME 9031, ME 9085. Gunung Kapor: ME 0234, ME 5972, ME 9204, ME 9213, ME 9251, ME 9842. Gunung Batu: ME 0226, ME 0229, ME 0703, ME 8795.

####### Distribution in Borneo.

Sarawak: Kuching Division. Endemic to Borneo.

####### Remarks.

Living snails were observed foraging on the wet vertical limestone rock surfaces covered with mosses and lichens. Shells from Jambusan areas are very similar to *Plectostoma dancei dispersum* (Vermeulen, 1994) but differ by having a deep red shell colour and fine spiral striations on the shell surface.

###### Plectostoma wallacei wallacei

Taxon classificationAnimaliaArchitaenioglossaDiplommatinidae

Ancey, 1887

A39144A6-42A9-5C5C-97B9-A4B3B674AACD

Figures 12F , 48B

Plectostoma wallacei Ancey, 1887: 276–277.

####### Type locality.

“Borneo”.

####### Material examined.

Gunung Doya: ME 0239, ME 9697. Gunung Kapor: ME 0218, ME 0240, ME 0242, ME 0243, ME 0244, ME 2915, ME 8066, ME 8482, ME 8759, ME 8767, ME 8780. Lobang Angin: ME 0246, ME 8730, ME 8733, ME 8743. Gunung Batu: ME 0245, ME 0247. Gunung Stulang: ME 5898. Kampung Padang Pan: ME 6669.

####### Distribution in Borneo.

Sarawak: Kuching and Serian divisions. Endemic to Borneo.

####### Remarks.

Living snails were observed foraging on the wet vertical limestone rock surfaces covered with mosses and lichens.

#### Family Pupinidae L Pfeiffer, 1853

##### *Pupina* Vignard, 1829

###### Pupina doriae

Taxon classificationAnimalia ArchitaenioglossaPupinidae

Godwin-Austen, 1889

C8B54A9F-9892-5FB9-9349-FCEED704A37B

Figure 14A

Pupina doriae Godwin-Austen, 1889: 351, pl. 39, figs 2, 2A, 2B.

####### Type locality.

“Busan Hills, Borneo” [= Jambusan Hills, Bau, Sarawak].

####### Material examined.

Gunung Doya: ME 1114, ME 8946, ME 9112. Gunung Kapor: ME 1116, ME 9234. Gunung Batu: ME 1115, ME 1121, ME 8809.

####### Distribution in Borneo.

Sarawak: Kuching and Serian divisions. Endemic to Borneo.

####### Remarks.

Living snails were observed foraging among leaf litter and plant debris near the cliff in lowland limestone forest. It differs from other Bornean *Pupina* species by having a smaller pearly white shell with a wide notch near the sutural margin.

**Figure 14.**
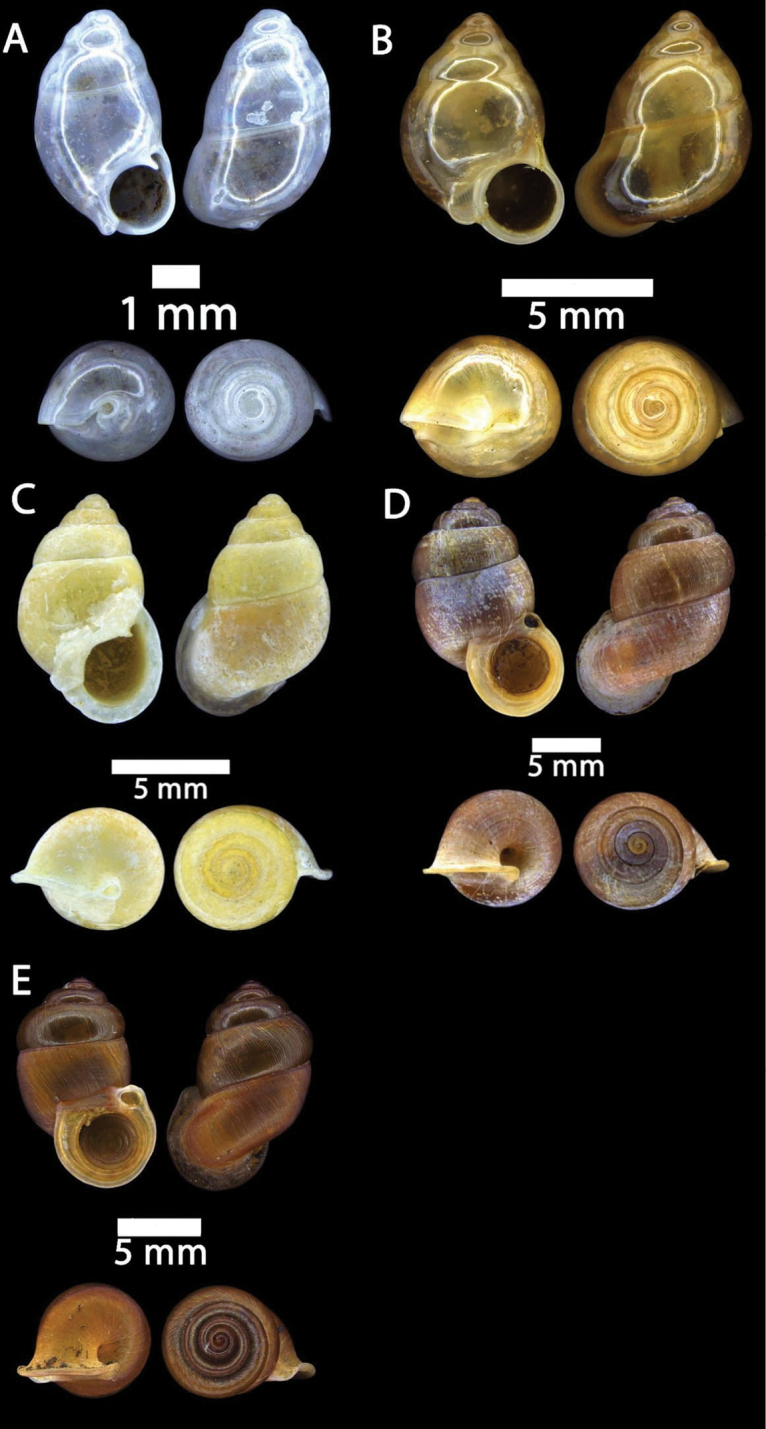
**A***Pupina doriae* Godwin-Austen, 1889 ME 8946 Gunung Doya **B***Pupina evansi* Godwin-Austen, 1889 ME 9053 Lobang Angin **C***Pupina hosei* Godwin-Austen, 1889 ME 1132 Gunung Batu **D***Rhaphaulus bombycinus* (L. Pfeiffer, 1855) ME 9556 Lobang Angin **E***Rhaphaulus pfeifferi* (Issel, 1874) ME 8741 Lobang Angin.

###### Pupina evansi

Taxon classificationAnimalia ArchitaenioglossaPupinidae

Godwin-Austen, 1889

54FE500E-6403-50C0-9FA4-B7F7446B57A9

Figures 14B , 49D

Pupina evansi Godwin-Austen, 1889: 351–352, pl. 39, figs 3, 3A.

####### Type locality.

“From deposit in Cave A, Borneo” [= Tupak Cave, Jambusan Hills (Cranbrook, 2013)].

####### Material examined.

Gunung Sebayat: ME 8006. Gunung Doya: ME 1129. Gunung Kapor: ME 1127, ME 1130, ME 8786, ME 9254. Lobang Angin: ME 9053, ME 9203.

####### Distribution in Borneo.

Sarawak: Kuching Division. ***Distribution elsewhere*.** Sirhassen, Natuna Islands (Smith 1894b).

####### Remarks.

Living snails were observed foraging among leaf litter and plant debris near the cliff in a lowland limestone forest. It differs from other Bornean *Pupina* species by having an intermediate size, globose, pale brown shell with a narrow notch near the sutural margin.

###### Pupina hosei

Taxon classificationAnimalia ArchitaenioglossaPupinidae

Godwin-Austen, 1889

BFA5AC30-EE20-56CA-AB53-B12A163A5404

Figure 14C

Pupina hosei Godwin-Austen, 1889: 351, pl. 39, figs 1, 1A.

####### Type locality.

“Busan Hills, Borneo” [= Jambusan Hills, Bau, Sarawak].

####### Material examined.

Gunung Batu: ME 1132, ME 1134.

####### Distribution in Borneo.

Sarawak: Kuching and Miri divisions. Sabah: Sandakan Division. ***Distribution elsewhere.*** Balabac and Palawan in Philippines (Kobelt and Möllendorff 1897).

####### Remarks.

Only dry shells were found during the surveys. It differs from other Bornean *Pupina* species by having a larger high spire dark brown shell with a wide notch near the sutural margin.

##### *Rhaphaulus* L. Pfeiffer, 1856

###### Rhaphaulus bombycinus

Taxon classificationAnimaliaArchitaenioglossaPupinidae

(L. Pfeiffer, 1855)

80A086E4-2D36-54B6-9B16-19EAC7389D96

Figure 14D

Anaulus bombycinus L. Pfeiffer 1855: 105–106, pl. 32, fig. 10.

####### Type locality.

“Borneo, Sarawak”.

####### Material examined.

Gunung Kapor: ME 1098, ME 1099, ME 3358, ME 9017, ME 9215. Lobang Angin: ME 9556. Gunung Batu: ME 1100, ME 1103.

####### Distribution in Borneo.

Sarawak: Kuching Division. Endemic to Borneo.

####### Remarks.

Living snails were observed foraging among leaf litter and plant debris near the cliff in lowland limestone forest. See remarks under *Rhaphaulus pfeifferi* (Issel, 1874).

###### Rhaphaulus pfeifferi

Taxon classificationAnimaliaArchitaenioglossaPupinidae

Issel, 1874

49C9C36F-58C7-505C-9BC5-B3EA41E085B2

Figure 14E

Raphaulus pfeifferi Issel, 1874: 443–444, pl. 7, figs 4–6.

####### Type locality.

“Territorio di Sarawak”.

####### Material examined.

Lobang Angin: ME 9556.

####### Distribution in Borneo.

Sarawak: Kuching Division. Endemic to Borneo.

####### Remarks.

Living snails were observed foraging among leaf litter and plant debris near the cliff in a lowland limestone forest. It differs from *R. bombycinus* by having a smaller shell with a less oblique spire and with a more spreading sutural tube.

#### Family Assimineidae H. Adams & A. Adams, 1856

##### *Acmella* W. T. Blanford, 1869

##### Acmella bauensis sp. nov.

Taxon classificationAnimaliaLittorinimorphaAssimineidae

EA93F4D6-C28D-58FD-BC4F-2F3426B7D052

http://zoobank.org/D854D239-B7FD-496D-9A84-AE7B1DCD2418

**Figure 15.**
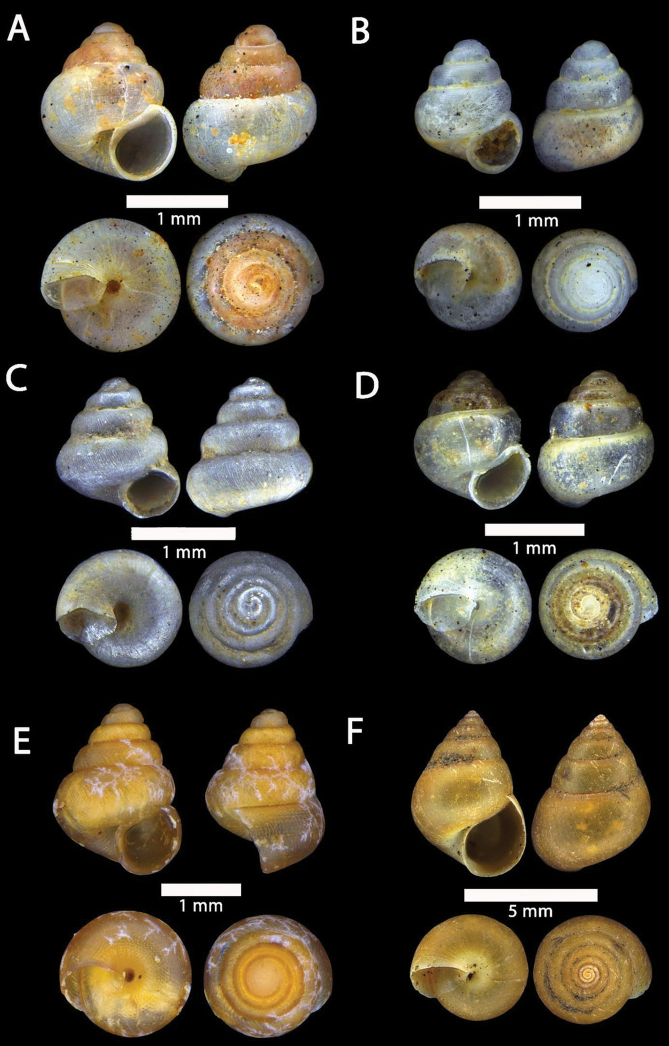
**A***Acmella bauensis*, sp. nov., MZU.MOL.20.01 holotype Gunung Kapor **B***Acmella cyrtoglyphe* Vermeulen, Liew & Schilthuizen, 2015 ME 8960 Gunung Doya [not in natural colour, shell surface coated with platinum for examination under scanning electron microscope] **C***Acmella nana* Vermeulen, Liew & Schilthuizen, 2015 ME 8497 Gunung Kapor **D***Acmella ovoidea* Vermeulen, Liew & Schilthuizen, 2015 ME 2215 Gunung Batu **E***Anaglyphula sauroderma* Vermeulen, Liew & Schilthuizen, 2015 ME 6910 Gunung Doya **F***Solenomphala scalaris* (Heude, 1882) ME 8810 Gunung Batu.

**Figure 16.**
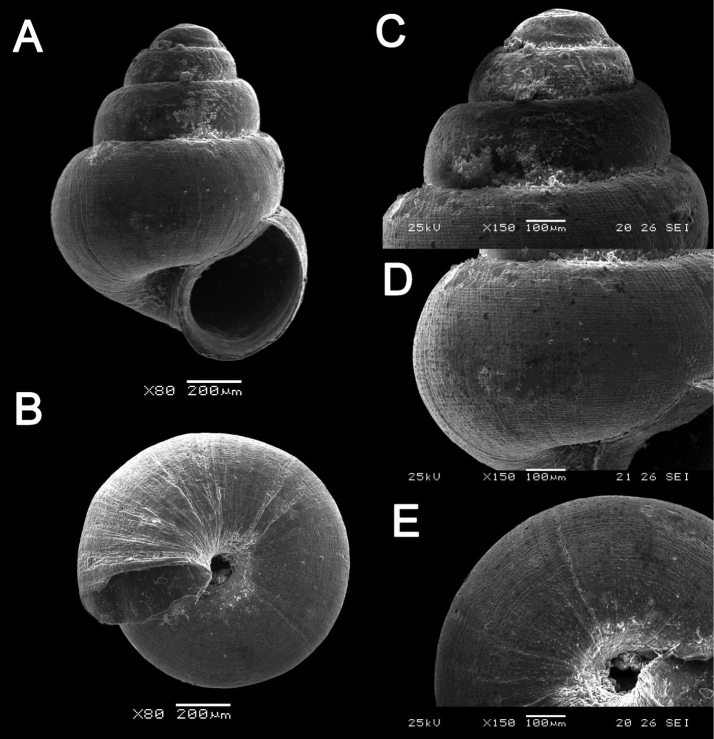
*Acmella bauensis* sp. nov. **A–F**MZU.MOL.20.01 holotype **A** apertural view **B** basal view **C** enlargement of the apical side showing the apex **D** enlargement of the body whorl showing the shell sculpture **E** enlargement of the basal side of the shell.

**Figure 17.**
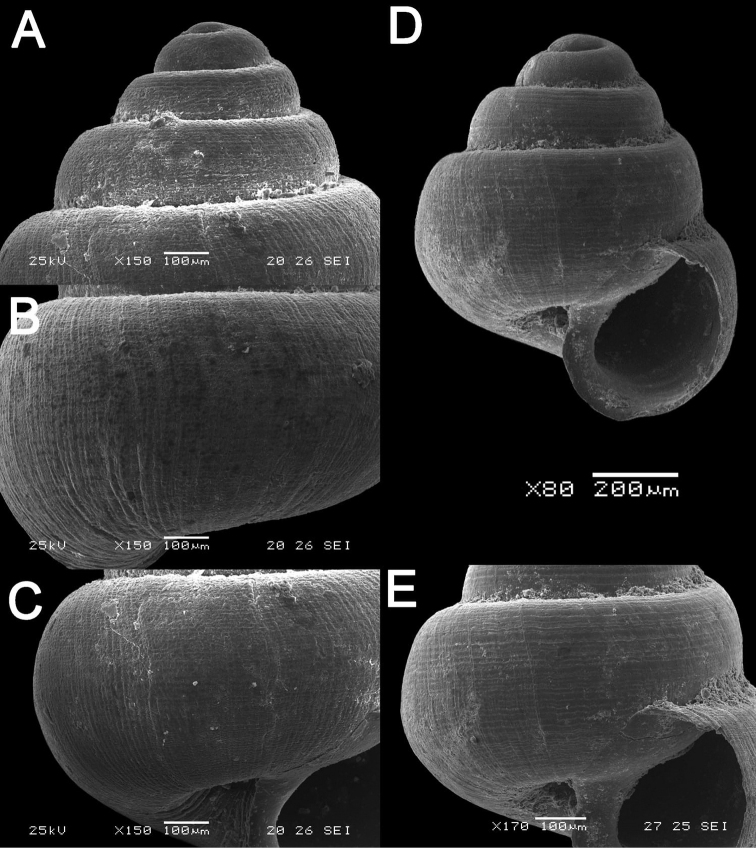
*Acmella bauensis* sp. nov. **A–C** ME0002340 paratype **A** enlargement of the apical side showing the apex **B** enlargement of the body whorl (abapertural side) showing the shell sculpture **C** enlargement of the body whorl (apertural side) showing the shell sculpture. *Acmella nana* Vermeulen, Liew, & Schilthuizen, 2015 **D–E** ME0009091 SPECIMEN **D** apertural view **E** enlargement of the body whorl (apertural side) showing the shell sculpture.

Figures 15A , 16A–E , 17A–C

###### Material examined.

***Holotype*** (SH 1.24 mm, SW 0.96 mm) (MZU.MOL.20.01), Malaysia, Sarawak, Kuching Division, Fairy Caves, south part of Gunung Kapor, 4 miles Southwest Bau, 1°22'53.97"N, 110°7'2.29"E, coll. M. E. Marzuki, 10.VII.2011. ***Paratypes***: 1 ex. (ME0002340), same data as Holotype.

###### Differential diagnosis.

This species differs from other Bornean *Acmella* species by having dull shell surface with inconspicuous, closely spaced spiral striae crossed by inconspicuous growth lines. *Acmella conica* Vermeulen & Junau, 2007 and *Acmella obtusa* Vermeulen & Junau, 2007, both from Central Sarawak, have a smaller shell with an elliptic aperture with a flattened parietal side. *Acmella minuttisima* (Maassen, 2000), from Sumatra, and *Acmella subcancellata* Vermeulen, Liew & Schilthuizen, 2015, from Sabah have more prominent spiral striae on the shell surface.

###### Description.

Shell minute, rather thin, translucent, and white. Surface not glossy. Spire conical with slightly convex side, apex rounded, whorls moderately convex, and slightly shouldered. Suture impressed. Protoconch sculpture microscopically cancellated. Teleoconch spiral sculpture with very fine, closely spaced, continuous spiral striae crossed by inconspicuous growth lines just visible at 80 times magnification. Aperture obliquely ovate in outline, with a concave to slightly convex parietal side, transition from parietal to basal side rounded. Peristome simple, expanded but not reflected on the columellar side. Umbilicus open, narrow, 0.08 mm in diameter. Dimensions: height < 1.24 mm; width < 0.96 mm; the number of whorls < 4; aperture height < 0.52 mm; aperture width < 0.44 mm.

###### Geographic distribution and habitat.

It is known only from the type locality. Only dry shells were found during the surveys.

###### Etymology.

The specific epithet *bauensis* is from the name of Bau District, where the shells were found.

##### Acmella cyrtoglyphe

Taxon classificationAnimaliaLittorinimorphaAssimineidae

Vermeulen, Liew & Schilthuizen, 2015

48D32CA2-CDB6-5E24-925D-4B42E6E3BE18

Figure 15B

Acmella cyrtoglyphe Vermeulen et al., 2015: 7–9, fig. 1A–D.

###### Type locality.

“Malaysia, Sabah, Interior Province, Sepulut valley, Gua Sanaron”.

###### Material examined.

Gunung Doya: ME 8960.

###### Distribution in Borneo.

Sabah: Interior, Sandakan and Tawau divisions. Sarawak: Kuching Division. Kalimantan: Exact location was not mentioned in Vermeulen et al. (2015). Endemic to Borneo.

###### Remarks.

Only dry shells were found during the surveys.

##### Acmella nana

Taxon classificationAnimaliaLittorinimorphaAssimineidae

Vermeulen, Liew & Schilthuizen, 2015

B7D9BBBB-5EF1-59B3-AFB9-B3891ECE60DA

Figures 15C , 17D, E

Acmella nana Vermeulen et al., 2015: 14, fig. 5A–D.

###### Type locality.

“Malaysia, Sarawak, 4^th^ Division, Niah Caves, Southside of limestone area, West side of the quarry, soil-filled crevice opened in quarry”.

###### Material examined.

Gunung Kapor: ME 8497. Gunung Doya: ME 9091.

###### Distribution in Borneo.

Sarawak: Kuching and Miri divisions. Sabah: Interior and Sandakan divisions. Endemic to Borneo.

###### Remarks.

Only dry shells were found during the surveys. The shells from Bau population are larger than the description of the type specimen (height 0.88 mm, width 0.76 mm).

##### Acmella ovoidea

Taxon classificationAnimaliaLittorinimorphaAssimineidae

Vermeulen, Liew & Schilthuizen, 2015

AD4E8359-65D4-56F0-A09B-5475BAA3BAFF

Figure 15D

Acmella ovoidea Vermeulen et al., 2015: 12–13, fig. 4A–D.

###### Type locality.

“Malaysia, Sabah, Interior Province, Pinangah valley, Batu Urun [= Bukit Sinobang]”.

###### Material examined.

Gunung Doya: ME 9103, ME 9104. Gunung Kapor: ME 1889, ME 8148, ME 9000, ME 9178, ME 9408. Kampung Padang Pan: ME 6673. Lobang Angin: ME 1081. Gunung Batu: ME 2215.

###### Distribution in Borneo.

Sabah: Interior, Sandakan and Tawau divisions. Sarawak: Kuching and Serian divisions. Kalimantan: East Kalimantan Province. Endemic to Borneo.

###### Remarks.

Only dry shells were found during the surveys.

####### *Anaglyphula* B. Rensch, 1932

##### Anaglyphula sauroderma

Taxon classificationAnimaliaLittorinimorphaAssimineidae

Vermeulen, Liew & Schilthuizen, 2015

B867AC09-98AD-54EE-9394-DA10B3B1BAEF

Figure 15E

Anaglyphula sauroderma Vermeulen et al., 2015: 18, fig. 8A–D.

###### Type locality.

“Malaysia, Sabah, Tawau Province, Batu Baturong ca. 50 km W.S.W. of Lahad Datu”.

###### Material examined.

Gunung Doya: ME 9143. Gunung Batu: ME 2141.

###### Distribution in Borneo.

Sarawak: Kuching Division. Sabah: Tawau Division. Kalimantan: East Kalimantan Province. Endemic to Borneo.

###### Remarks.

This is the first record of this species in Sarawak. Living snails were observed foraging on wet rotten wood surfaces at the base of the limestone cliff.

####### *Solenomphala* Heude, 1882

##### Solenomphala scalaris

Taxon classificationAnimaliaLittorinimorphaAssimineidae

(Heude, 1882)

65742F28-2FBA-5AB2-B3FD-2B0E9EAEB307

Figure 15F

Assiminea (Solenomphala) scalaris Heude, 1882: 83–84, pl. 21, figs 5, 5a–c.

###### Type locality.

“Ad parietes humidos in civitate Chang-hai sat copiosa” [= Shanghai, China].

###### Material examined.

Gunung Kapor: ME 8091, ME 9231. Gunung Batu: ME 2216, ME 2339, ME 8810.

###### Distribution in Borneo.

Sarawak: Kuching, Samarahan, and Mukah divisions. ***Distribution elsewhere.*** East to Southeast Asia (Fukuda and Ponder 2003).

###### Remarks.

This amphibious snail is an introduced species because all the known records were found in the damp area among human settlements (Chan 1997; this study). The shell form is similar to that of *Cyclotropis bollingi* Brandt, 1974, but differs by having fine spiral sculptures.

#### Family Hydrocenidae Troschel, 1857

##### *Georissa* W. T. Blanford, 1864

###### Georissa bauensis

Taxon classificationAnimaliaCycloneritidaHydrocenidae

Khalik, Hendriks, Vermeulen & Schilthuizen, 2018

FC182359-7744-5437-9E54-D9443FDFBA75

Figures 18A , 50A

Georissa bauensis Khalik et al., 2018: 42.

####### Type locality.

“Wind Cave Passage 3, Wind Cave Nature Reserve, Bau, Sarawak, Malaysia”.

####### Material examined.

Lobang Angin: ME 0908, ME 8731, ME 8736.

####### Distribution in Borneo.

Sarawak: Kuching Division. Endemic to Borneo.

####### Remarks.

Living snails were observed foraging on the wet vertical limestone rock surfaces covered with mosses and lichens.

**Figure 18.**
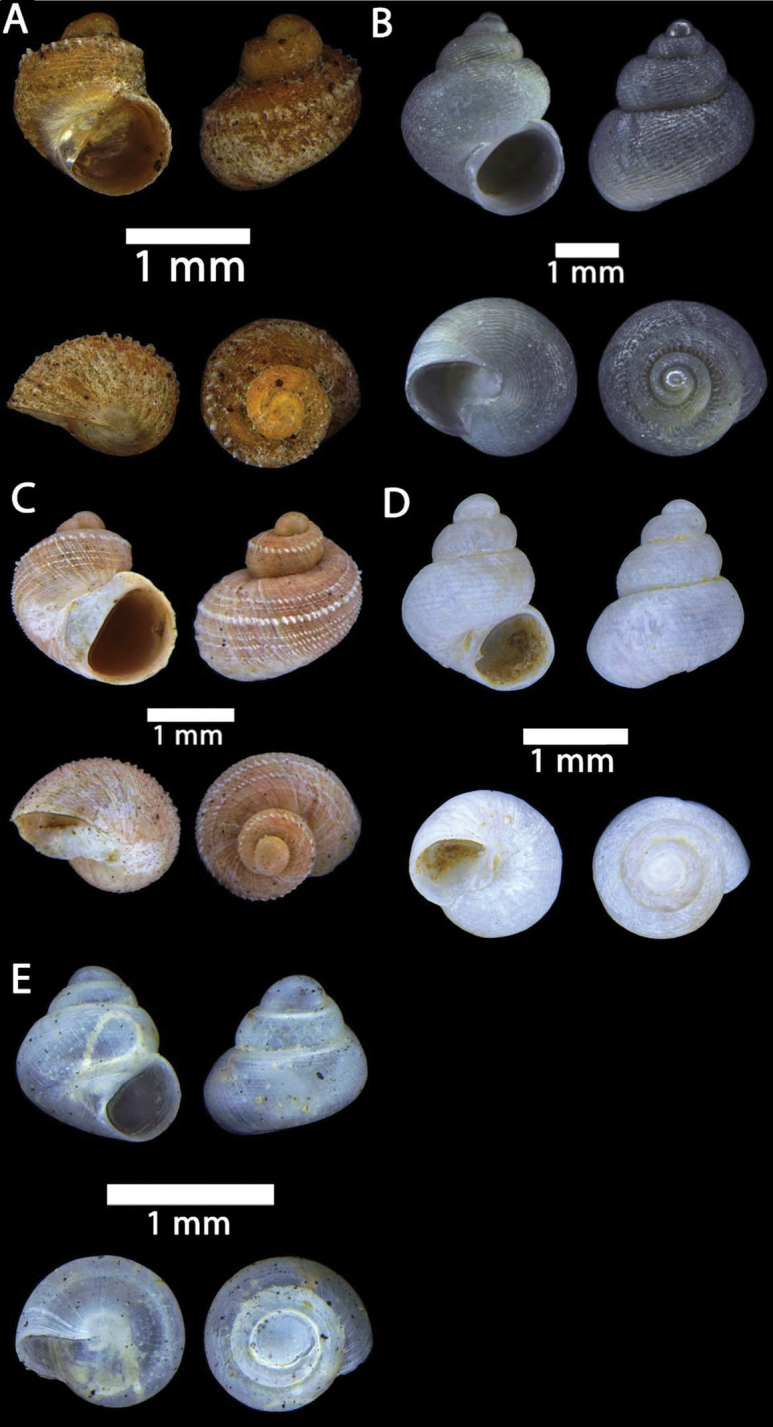
**A***Georissa bauensis* Khalik, Hendricks, Vermeulen & Schilthuizen, 2018 ME 8731 Lobang Angin **B***Georissa everetti* E. A. Smith, 1895 ME 0906 Gunung Batu [not in natural colour, shell surface coated with platinum for examination under scanning electron microscope] **C***Georissa hosei* Godwin-Austen, 1889 ME 8498 Gunung Kapor **D***Georissa hungerfordi* Godwin-Austen, 1889, ME 11472 Gunung Kapor **E***Georissa leucococca* Vermeulen, Liew & Schilthuizen, 2015 ME 9496 Gunung Doya.

###### Georissa everetti

Taxon classificationAnimaliaCycloneritidaHydrocenidae

E. A. Smith, 1895

6B5A65AF-1C48-5211-83BE-6F22168D3FC7

Figure 18B

Georissa everetti E. A. Smith, 1895: 125, pl. 4, fig. 15.

####### Type locality.

“Rumbang, W. Sarawak”.

####### Material examined.

Gunung Batu: ME 0906.

####### Distribution in Borneo.

Sarawak: Kuching and Miri divisions. Endemic to Borneo.

####### Remarks.

Only dry shells were found during the surveys.

###### Georissa hosei

Taxon classificationAnimaliaCycloneritidaHydrocenidae

Godwin-Austen, 1889

B93F8234-16D4-538D-8F12-F8F3D0D57258

Figure 18C

Georissa hosei Godwin-Austen, 1889: 353, pl. 39, fig. 11.

####### Type locality.

“Borneo”.

####### Material examined.

Gunung Doya: ME 1478. Gunung Kapor: ME 8498.

####### Distribution in Borneo.

Sarawak: Kuching Division. Endemic to Borneo.

####### Remarks.

Only dry shells were found during the surveys.

###### Georissa hungerfordi

Taxon classificationAnimaliaCycloneritidaHydrocenidae

Godwin-Austen, 1889

E2F8F295-6992-517A-BDA5-121CF1AC0D29

Figure 18D

Georissa hungerfordi Godwin-Austen, 1889: 354, pl. 39, fig. 9.

####### Type locality.

“Borneo”.

####### Material examined.

Gunung Kapor: ME 11472.

####### Distribution in Borneo.

Sarawak: Kuching Division. Endemic to Borneo.

####### Remarks.

Only dry shells were found during the surveys.

###### Georissa leucococca

Taxon classificationAnimaliaCycloneritidaHydrocenidae

Vermeulen, Liew & Schilthuizen, 2015

DAC9EB74-D36D-5198-B74D-22546B1E352C

Figure 18E

Georissa leucococca Vermeulen et al., 2015: 33, fig. 19A, B.

####### Type locality.

“Malaysia, Sabah, Interior Province, Sepulut valley, Gua Pungiton”.

####### Material examined.

Gunung Doya: ME 9701, ME 9496.

####### Distribution in Borneo.

Sarawak: Kuching and Serian divisions. Sabah: Interior and Tawau divisions. Endemic to Borneo.

####### Remarks.

Only dry shells were found during the surveys.

#### Family Achatinellidae Gulick, 1873

##### *Elasmias* Pilsbry, 1910

###### Elasmias sundanum

Taxon classificationAnimaliaStylommatophoraAchatinellidae

(Möllendorff, 1897)

540CE328-E5DA-5BAF-9682-92972F41625D

Figure 19A

Tornatellina sundana Möllendorff, 1897a: 90.

####### Type locality.

“Java”.

####### Material examined.

Kampung Padang Pan: ME 6828. Lobang Angin: ME 8982.

####### Distribution in Borneo.

Sarawak: Kuching and Serian divisions. ***Distribution elsewhere.*** Sumatra to Java (Van Benthem-Jutting 1952)

####### Remarks.

This is the first record of this species in Borneo. Only dry shells were found during the surveys.

**Figure 19.**
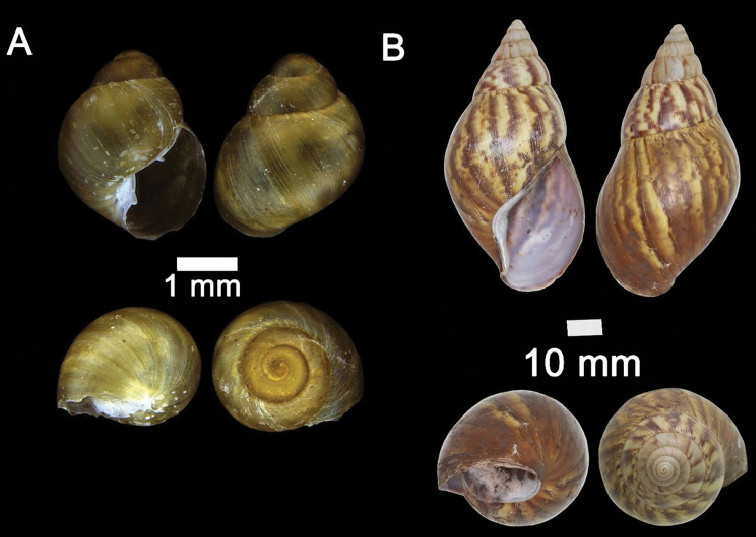
**A***Elasmias sundanum* (Möllendorff, 1897) ME 8982 Lobang Angin **B***Lissachatina fulica* (Bowdich, 1822) ME 9241 Gunung Kapor.

#### Family Achatinidae Swainson, 1840

##### *Lissachatina* Bequaert, 1950

###### Lissachatina fulica

Taxon classificationAnimaliaStylommatophoraAchatinidae

(Bowdich, 1822)

3EFF86E2-B4F2-52BC-BF74-73E2B570AD1B

Figure 19B

Achatina fulica Bowdich, 1822: pl. 13, fig. 3.

####### Type locality.

“L’ile de France” [= Mauritius].

####### Material examined.

Gunung Doya: ME 8920, ME 9228. Gunung Kapor: ME 8507, ME 9016, ME 9241.

####### Distribution in Borneo.

Sarawak: Kuching, Samarahan, Serian, Sibu, Mukah and Miri divisions. Sabah: Kudat, West Coast, Interior, Sandakan and Tawau divisions. Kalimantan. ***Distribution elsewhere.*** Circumtropical (Vermeulen and Whitten 1998).

####### Remarks.

Living snails were observed foraging among leaf litter and plant debris near the cliff in lowland limestone forest. The species was firstly introduced into Sarawak in 1928 as poultry food and became pest a year later (Jarrett 1931). Apparently widespread throughout Borneo.

##### *Allopeas* Baker, 1935

###### Allopeas clavulinum

Taxon classificationAnimaliaStylommatophoraAchatinidae

(Potiez & Michaud, 1838)

D7A2EE5A-EFC1-5C6A-A69B-F6C6229903A7

Figure 20A

Bulimus clavulinus Potiez & Michaud, 1838: 136.

####### Type locality.

“L’ile Bourbon” [=La Réunion].

####### Material examined.

Gunung Sebayat: ME 8013. Gunung Doya: ME 2905, ME 9704, ME 8928, ME 8948, ME 9036. Gunung Kapor: ME 0753, ME 2877, ME 2948, ME 8506, ME 9043, ME 9229, ME 9248. Lobang Angin: ME 9105, ME 9276. Gunung Batu: ME 2835, ME 2841, ME 2870, ME 8821.

####### Distribution in Borneo.

Sarawak: Kuching, Serian, Sibu, and Miri divisions. Sabah: Interior, Sandakan, Tawau, and West Coast divisions. ***Distribution elsewhere.*** Circumtropical (Vermeulen and Whitten 1998).

####### Remarks.

Living snails were observed foraging among leaf litter and plant debris near the cliff in a lowland limestone forest. Introduced species. Widespread throughout Borneo.

**Figure 20.**
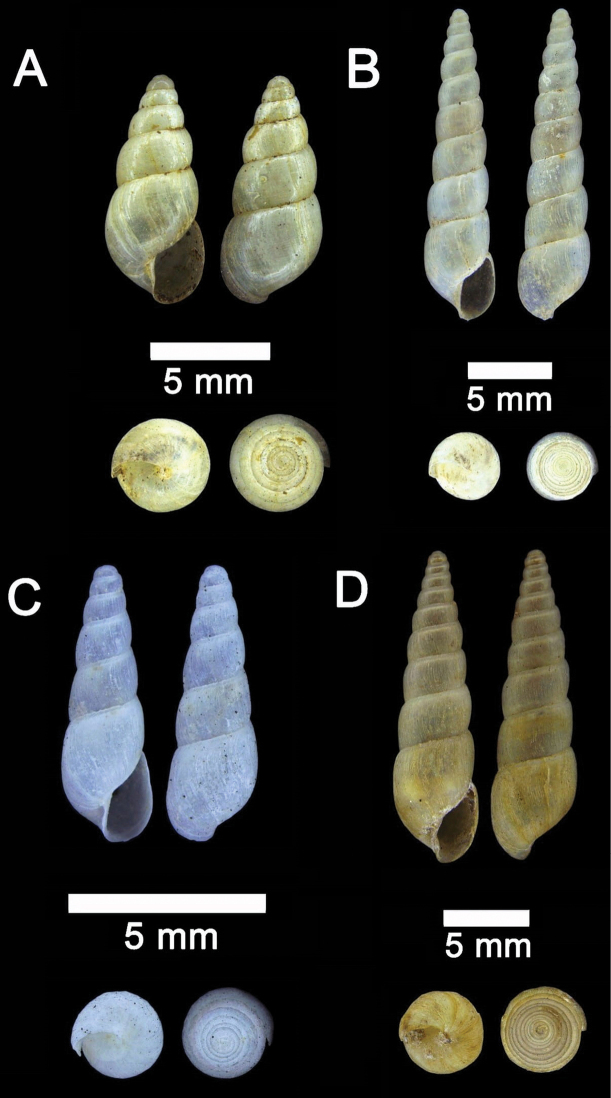
**A***Allopeas clavulinum* (Potiez & Michaud, 1838) ME 0753 Gunung Kapor **B***Allopeas gracile* (T. Hutton, 1834) ME 2942 Gunung Kapor **C***Opeas didyma* (Westerlund, 1883) ME 9096 Gunung Kapor **D***Paropeas achatinaceum* (L. Pfeiffer, 1846) ME 5977 Gunung Kapor.

###### Allopeas gracile

Taxon classificationAnimaliaStylommatophoraAchatinidae

(T. Hutton, 1834)

96192863-F468-50AA-9CF3-7AF49C039A4D

Figures 20B , 54B

Bulimus gracilis T. Hutton, 1834: 84–85, 93.

####### Type locality.

“Mirzapoor, India”.

####### Material examined.

Gunung Doya: ME 8929, ME 8949, ME 9035. Gunung Kapor: ME 0312, ME 1229, ME 2942, ME 2943, ME 8149, ME 8504, ME 8970, ME 9011, ME 9069. Kampung Padang Pan: ME 6681. Lobang Angin: ME 9135, ME 9263. Gunung Batu: ME 2842, ME 2866, ME 8822.

####### Distribution in Borneo.

Sarawak: Kuching, Serian, Mukah, and Miri divisions. Sabah: Interior, Kudat, Sandakan, Tawau, and West Coast divisions. Kalimantan: West Kalimantan Province. ***Distribution elsewhere.*** Circumtropical (Vermeulen and Whitten 1998).

####### Remarks.

Living snails were observed foraging among leaf litter and plant debris near the cliff in a lowland limestone forest. Introduced species. Widespread throughout Borneo.

##### *Opeas* Albers, 1850

###### Opeas didyma

Taxon classificationAnimaliaStylommatophoraAchatinidae

(Westerlund, 1883)

7FD7A760-C599-5ECE-8163-7798ED9DFC2C

Figure 20C

Stenogyra didyma Westerlund, 1887: 197–198, pl. 3, fig. 9.

####### Type locality.

“Malakka, Singapore”.

####### Material examined.

Gunung Kapor: ME 9096, ME 9237.

####### Distribution in Borneo.

Sarawak: Kuching Division. ***Distribution elsewhere.*** West Malaysia and Singapore (Westerlund 1887)

####### Remarks.

Living snails were observed foraging among leaf litter and plant debris near the cliff in a lowland limestone forest.

##### *Paropeas* Pilsbry, 1906

###### Paropeas achatinaceum

Taxon classificationAnimaliaStylommatophoraAchatinidae

(L. Pfeiffer, 1846)

E1D9B477-7D57-5B0E-95C6-FF3D9340C387

Figures 20D , 54A

Bulimus achatinaceus L. Pfeiffer, 1846a: 82.

####### Type locality.

“Java”.

####### Material examined.

Gunung Kapor: ME 2876, ME 2878, ME 2946, ME 2951, ME 5977, ME 8086, ME 8505, ME 8785, ME 8964. Lobang Angin: ME 8983, ME 9172, ME 9269. Gunung Batu: ME 8823.

####### Distribution in Borneo.

Sarawak: Kuching, Serian, Kapit, and Miri divisions. Labuan, Sabah: Sandakan and West Coast divisions. Kalimantan: West Kalimantan Province. ***Distribution elsewhere.*** South to East Asia, South-east Asia, Pacific Islands (Naggs 1994; Vermeulen and Whitten 1998).

####### Remarks.

Living snails were observed foraging among leaf litter and plant debris near the cliff in a lowland limestone forest. Widespread throughout Borneo.

#### Family Diapheridae Panha & Naggs, 2010

##### *Platycochlium* Laidlaw, 1950

###### Platycochlium sarawakense

Taxon classificationAnimaliaStylommatophoraDiapheridae

Laidlaw, 1950

4AFE7C8A-B7F3-58EA-9AAF-C352B548BE52

Figure 21A

Platycochlium sarawakense Laidlaw, 1950: 370–372, fig. 1A, B.

####### Type locality.

“Gunong Kapor, Bau District, Sarawak”.

####### Material examined.

Gunung Doya: ME 2906, ME 8930, ME 8950, ME 8993. Gunung Kapor: ME 8150, ME 8503, ME 9050. Gunung Batu: ME 2838, ME 2843, ME 2871, ME 8819.

####### Distribution in Borneo.

Sarawak: Kuching and Serian divisions. Endemic to Borneo.

####### Remarks.

Living snails were observed foraging among leaf litter and plant debris near the cliff in a lowland limestone forest.

**Figure 21.**
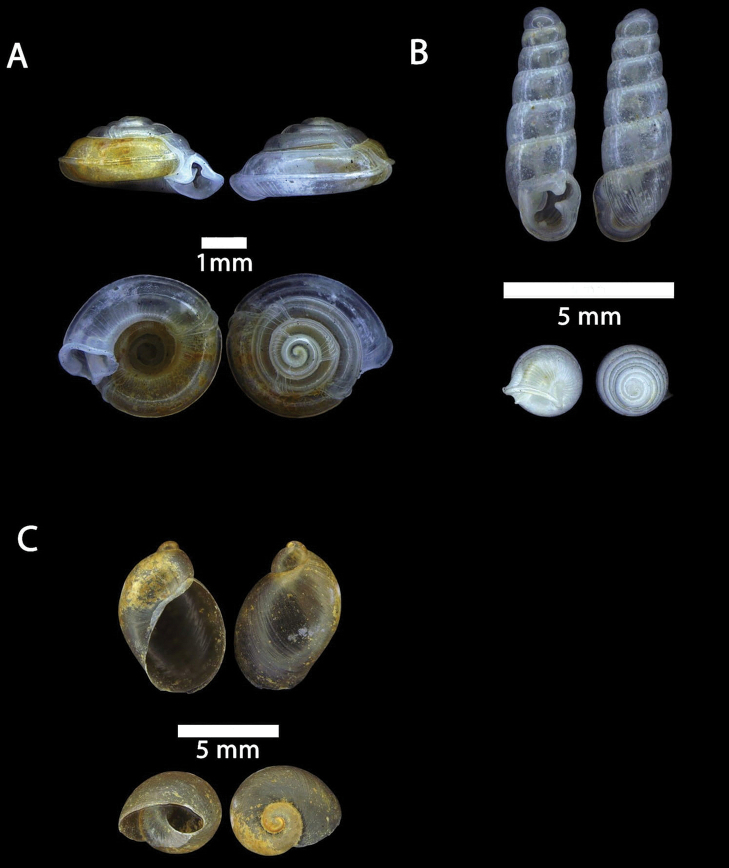
**A***Platycochlium sarawakense* Laidlaw, 1950 ME 8993 Gunung Doya **B***Gulella bicolor* (T. Hutton, 1834) ME 9095 Gunung Kapor **C***Succinea obesa* Martens, 1867 ME 2903 Gunung Batu.

#### Family Streptaxidae Gray, 1860

##### *Gulella* L. Pfeiffer, 1856

###### Gulella bicolor

Taxon classificationAnimaliaStylommatophoraStreptaxidae

(T. Hutton, 1834)

10DBA90F-6F5B-5004-A7AB-E8D30D2B14D8

Figure 21B

Pupa bicolor T. Hutton, 1834: 86, 93.

####### Type locality.

“Mirzapoor, India”.

####### Material examined.

Gunung Kapor: ME 9095.

####### Distribution in Borneo.

Sabah: Interior, Kudat, Tawau, and West Coast divisions. Sarawak: Kuching, Samarahan, Serian, Sibu, and Miri divisions. ***Distribution elsewhere.*** South to East Asia, Malay Archipelago (Vermeulen and Whitten 1998).

####### Remarks.

Only dry shells were found during the surveys. Introduced species.

#### Family Succineidae Beck, 1837

##### *Succinea* Draparnaud, 1801

###### Succinea obesa

Taxon classificationAnimaliaStylommatophoraSuccineidae

(Martens, 1867)

AD735D70-61EB-5EB2-BD92-AE27766B77A6

Figure 21C

Succinea obesa Martens, 1867: 387, pl. 22, fig. 21.

####### Type locality.

“Oestliches Java, am See von Grati bei Passuruan gesammelt”.

####### Material examined.

Gunung Batu: ME 2903, ME 8820.

####### Distribution in Borneo.

Sarawak: Kuching Division. ***Distribution elsewhere.*** Sumatra, Java, and Madura, Indonesia (Van Benthem-Jutting 1952).

####### Remarks.

Only dry shells were found during the surveys. The shells from Bau match the descriptions of *Succinea obesa* Martens, 1867 sensu Van Benthem-Jutting (1952). Two other species, namely *Succinea borneensis* Pfeiffer, 1853 and *Succinea subrugata* Pfeiffer, 1853, were described from Borneo. However, the original descriptions of these two species were not detailed enough for species identification.

#### Family Ariophantidae Godwin-Austen, 1883

##### *Damayantia* Issel, 1874

###### Damayantia carinata

Taxon classificationAnimaliaStylommatophoraAriophantidae

Collinge, 1901

9A080F60-5A37-51BD-9473-A37676DA3E94

Figures 22A , 51A

Damayantia carinata Collinge, 1901: 298–299, pl. 1, figs 4, 5 and pl. 2, figs 22, 23.

####### Type locality.

“Kuching, Mt. Penrissen, and Mt. Santubong, N.W. Borneo”.

####### Material examined.

Gunung Doya: ME 8922. Lobang Angin: ME 9054.

####### Distribution in Borneo.

Sarawak: Kuching and Sibu divisions. Endemic to Borneo.

####### Remarks.

It is an arboreal semi-slug which can also be found in lowland peat swamp forests.

**Figure 22.**
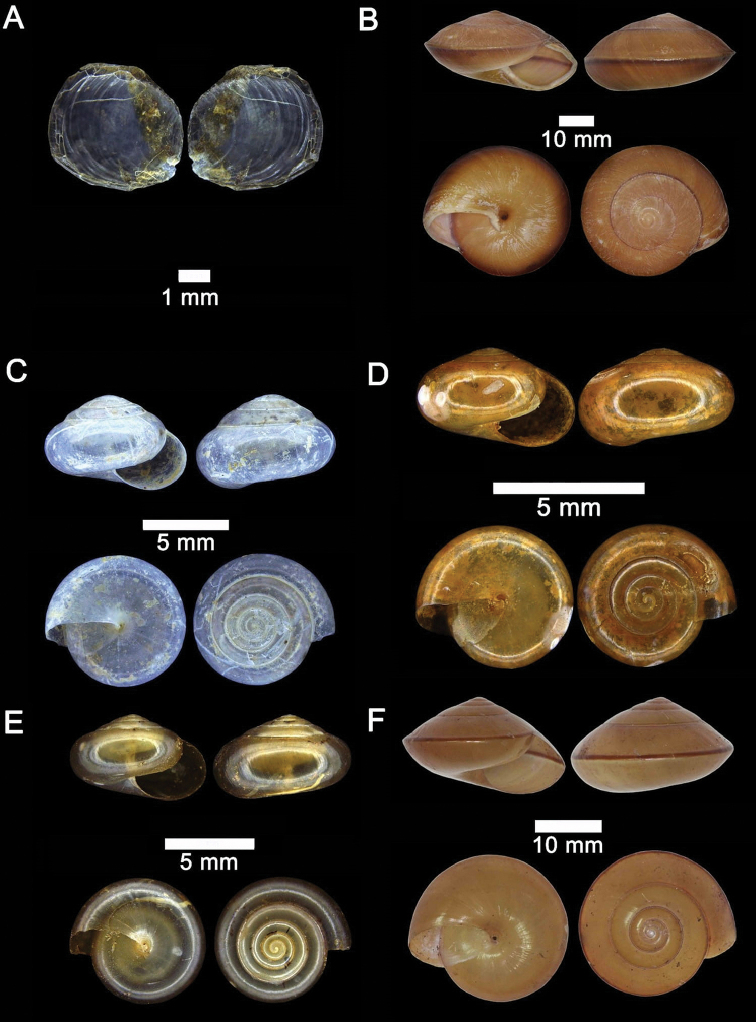
**A***Damayantia carinata* Collinge, 1901 ME 9054 Lobang Angin **B***Hemiplecta densa* (A. Adams & Reeve, 1850) ME 9241 Gunung Doya **C***Macrochlamys infans* (L. Pfeiffer, 1854) ME 2854 Gunung Batu **D***Macrochlamys saintjohni* (Godwin-Austen, 1891) ME 9900 Gunung Kapor **E***Macrochlamys tersa* (Issel, 1874) ME 9620 Gunung Kapor **F***Vitrinula glutinosa* (Metcalfe, 1851) ME 8782 Gunung Kapor.

##### *Hemiplecta* Albers, 1850

###### Hemiplecta densa

Taxon classificationAnimaliaStylommatophoraAriophantidae

(A. Adams & Reeve, 1850)

CE354587-9432-53C6-BA42-72E91D02725B

Figures 22B , 51C

Helix densa A. Adams & Reeve, 1850: 62, pl. 16, fig. 8.

####### Type locality.

“Philippine Islands”.

####### Material examined.

Gunung Doya: ME 8892, ME 9160. Gunung Kapor: ME 2581, ME 4723, ME 4724, ME 5971, ME 8079, ME 8791, ME 9243. Gunung Stulang: ME 5907. Kampung Padang Pan: ME 6674. Lobang Angin: ME 9485. Gunung Batu: ME 4725, ME 4726, ME 8833.

####### Distribution in Borneo.

Sarawak: Kuching Division. Labuan, Sabah: Sandakan Division. Kalimantan: West and East Kalimantan provinces. ***Distribution elsewhere.*** Philippines and Indonesia: Java and Sumatra (Mousson 1857; Smith 1895).

####### Remarks.

Further study is needed to clarify the taxonomic status of this species together with *Hemiplecta humpreysiana* (I. Lea, 1840).

##### *Macrochlamys* Gray, 1847

###### Macrochlamys infans

Taxon classificationAnimaliaStylommatophoraAriophantidae

(L. Pfeiffer, 1854)

A77327E4-646C-580D-A0A9-B440678468FC

Figure 22C

Helix infans L. Pfeiffer, 1854a: 290.

####### Type locality.

“Sarawak, Borneo”.

####### Material examined.

Gunung Batu: ME 1858, ME 2854.

####### Distribution in Borneo.

Sarawak: Kuching Division. Labuan: Endemic to Borneo.

####### Remarks.

Only dry shells specimens were found during the surveys. It differs from *Macrochlamys tersa* (Issel, 1874) by having a white shell without spiral sculpture or only with very fine spiral grooves. The taxonomic status and occurrence of *M. infans* in Java and Bali were discussed in Vermeulen (1996).

###### Macrochlamys sainctjohni

Taxon classificationAnimaliaStylommatophoraAriophantidae

(Godwin-Austen, 1891)

8DB71F92-AFAA-5E91-81B5-2CA475170899

Figure 22D

Microcystina st. johni Godwin-Austen, 1891: 38, pl. 4, figs 3, 3A.

####### Type locality.

“Busan Hills, Borneo”.

####### Material examined.

Gunung Sebayat: ME 8011. Gunung Doya: ME 3053, ME 9111, ME 9184. Gunung Kapor: ME 3850, ME 8509, ME 9208, ME 9240, ME 9900. Gunung Batu: ME 2837.

####### Distribution in Borneo.

Sarawak: Kuching Division. ***Distribution elsewhere.*** Palawan (Smith 1895).

####### Remarks.

Living snails were observed foraging among leaf litter and plant debris near the cliff in a lowland limestone forest. It differs from other Bornean *Macrochlamys* species by having a small reddish brown shell with well-spaced fine spiral grooves.

###### Macrochlamys tersa

Taxon classificationAnimaliaStylommatophoraAriophantidae

(Issel, 1874)

7A5AE932-9DAB-5CBE-BFDF-0FE7FC515B28

Figures 22E , 51B

Nanina (Macrochlamys) tersa Issel, 1874: 399–400, pl. 5, figs 1–4.

####### Type locality.

“Borneo”.

####### Material examined.

Gunung Doya: ME 8936, ME 8957, ME 9037. Gunung Kapor: ME 2880, ME 2882, ME 2945, ME 3040, ME 8089, ME 8771, ME 8966, ME 9048, ME 9620. Kampung Padang Pan: ME 6680. Lobang Angin: ME 9142, ME 9152, ME 9272. Gunung Batu: ME 1858, ME 2855, ME 2868, ME 8827.

####### Distribution in Borneo.

Sarawak: Kuching, Serian, and Miri divisions. Sabah: Kudat, West Coast, Interior, and Tawau divisions. Endemic to Borneo.

####### Remarks.

Living snails were observed foraging among leaf litter and plant debris near the cliff in a lowland limestone forest. It differs from other Bornean *Macrochlamys* species by having a moderately sized pale brown shell with very fine closely spaced spiral grooves.

##### *Microcystina* Mörch, 1872

###### Microcystina arabii sp. nov.

Taxon classificationAnimaliaStylommatophoraAriophantidae

09CE9751-97D6-52F1-88C8-7DFFE9742CBE

http://zoobank.org/A9C3898C-E203-427A-B1B3-1350F235EB3C

Figures 23A , 24A–F

####### Material examined.

***Holotype*** (SH 1.38 mm, SW 1.75 mm) (MZU.MOL.20.06), Malaysia, Sarawak, Kuching Division, Bukit Sokwang (Site 2), northern site of Gunung Doya, limestone hill along Skio road, 2.05 miles E Bau, 1°23'45.69"N, 110°10'35.04"E, coll. M. E. Marzuki, 22.IV.2017. ***Paratypes***: 1 ex. (ME0009154), same data as the holotype; 9 ex. (ME0009165), Bukit Sokwang (Site 1), northern site of Gunung Doya, limestone hill along Skio road, 2.05 miles E Bau, 1°23'52.11"N, 110°10'27.93"E, coll. M. E. Marzuki, 22.IV.2017; 6 ex. (ME0009168), Bukit Sokwang (Site 3), northern site of Gunung Doya, limestone hill along Skio road, 2.05 miles E Bau, 1°23'49.87"N, 110°10'32.14"E, coll. M. E. Marzuki, 22.IV.2017; 2 ex. (ME0002899), Gunung Batu, limestone outcrop along Skio road, Jambusan, 2.4 miles E Bau, 1°23'50.65"N, 110°11'19.99"E, coll. M. E. Marzuki, 23.VI.2010; 4 ex. (ME0001757), the same locality, coll. M. E. Marzuki, 11.III.2011; 1 ex. (ME0008889), the same locality, coll. M. E. Marzuki, 10.II.2017; 2 ex (ME0002899), Fairy Caves (Site 1), south part of Gunung Kapor, 4 miles SW Bau, 1°22'53.76"N, 110°7'4.34"E, coll. M. E. Marzuki, 23.VI.2010; 2 ex. (ME0001762), 2 ex. (MZU.MOL.20.07), South Flank of Bukit Akud, near Kampung Beratok, Serian-Kuching road, 14 miles NW Serian, 1°18'23.26"N, 110°24'15.07"E, coll. M. E. Marzuki, 21.VI.2010; 7 ex. (ME0007020), small limestone outcrop at Kampung Beratok, Serian-Kuching road, 14.3 miles NW Serian, 1°18'41.05"N, 110°24'37.13"E, coll. M. E. Marzuki, 27.XII.2015; 1 ex. (ME0011489), Lobang Angin (Site 1), limestone outcrop near Sungai Sarawak Kanan, 1.75 miles W of Bau, 1°24'48.14"N, 110°8'12.21"E, coll. M. E. Marzuki, 15.IV.2017; 6 ex. (ME0008138), Limestone escarpment near Kampung Benuk, 8.2 miles SW Kota Padawan, coll. M. E. Marzuki, 13.IX.2017.

**Figure 23.**
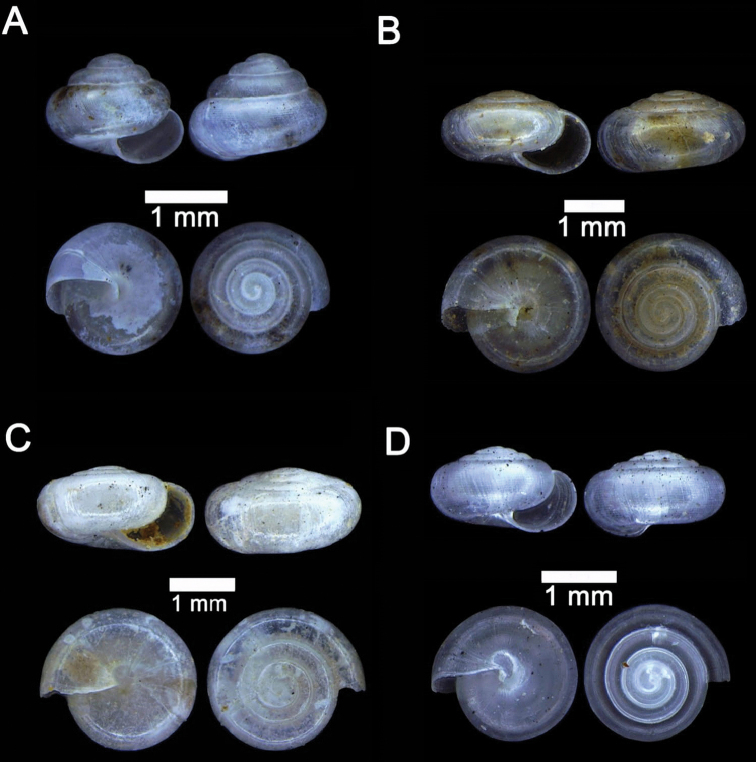
**A***Microcystina arabii*, sp. nov., MZU.MOL.20.06 Holotype Gunung Batu **B***Microcystina atoni*, sp. nov., MZU.MOL.20.11 Paratype Gunung Batu **C***Microcystina kilat*, sp. nov., MZU.MOL.20.19 Paratype Gunung Batu **D***Microcystina lirata*, sp. nov., MZU.MOL.20.15 paratype Gunung Kapor.

####### Differential diagnosis.

It differs from *Microcystina sinica* Möllendorff, 1885, *Microcystina oswaldbrakeni* sp. nov., and *Microcystis bunguranensis* Smith, 1894 by having cancellated sculpture on the shell surface.

####### Description.

Shell very small, thin, translucent, white, straw yellow to brown; spire distinctly elevated, conical with convex sides or depressed-ovoid; apex rounded. Surface with a silky to glossy lustre. Whorls convex, periphery rounded. Last whorl with a thin, inconspicuous peripheral thread coinciding with the suture of the penultimate whorl. Number of whorls < 4¼. Protoconch: with a fine, moderately spaced spiral striation consisting of rows of minute spiral grooves crossed by equally strong radial riblets which are arranged in a cancellated pattern towards the teleoconch. Teleoconch moderately spaced spiral striation consisting of rows of minute spiral grooves crossed by equally strong radial riblets which are arranged in a cancellated pattern. Radial sculpture teleoconch of very fine, densely spaced radial riblets, oblique, predominant at the peripheral region and inconspicuous at the umbilical region. Aperture: lunulate. Peristome simple; somewhat thickened and reflected on the columellar side, not thickened nor reflected on the basal and palatal side. Umbilicus open, narrow, partly covered; umbilical region moderately concave. Dimensions: shell height < 1.38 mm; shell width < 1.75 mm; diameters of the first three whorls 0.38–0.42 mm, 0.75–0.83 mm, and 1.17–1.29 mm, respectively; aperture height < 0.67 mm; aperture width < 0.92 mm.

**Figure 24.**
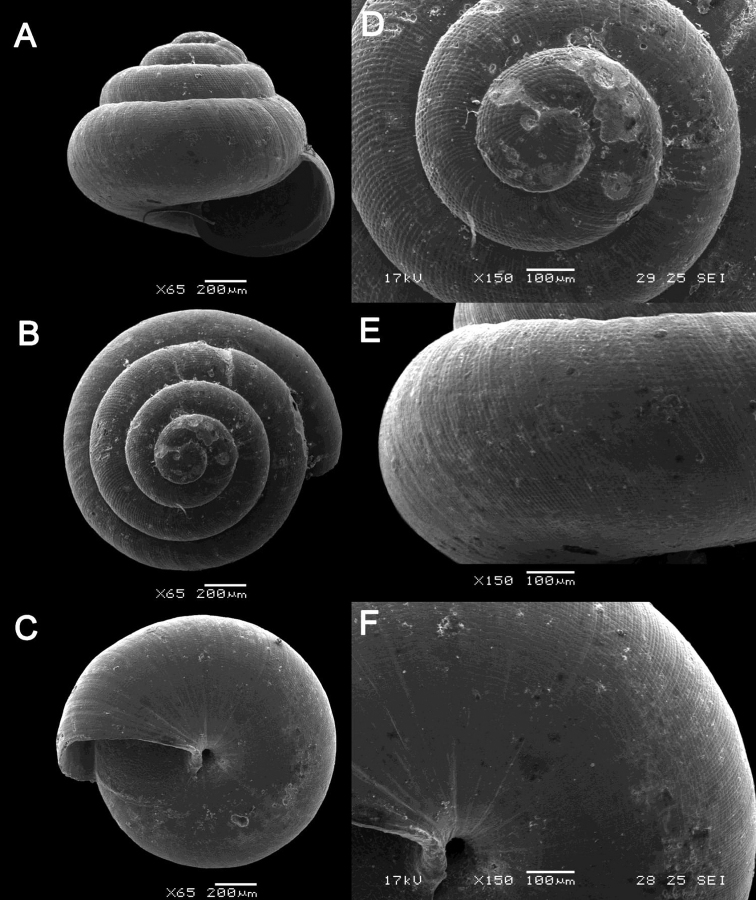
*Microcystina arabii* sp. nov. **A–F**MZU.MOL.20.07 Paratype **A** Apertural view **B** Apical view, **C** Basal view **D** Enlargement of the apical side showing the apex **E** Enlargement of the body whorl showing the shell sculpture **F** Enlargement of the basal side of the shell.

####### Remarks.

The shells of this species display high variability in terms of colour and height/width ratio.

####### Geographic distribution and habitat.

It is known from the Bau and Padawan-Serian limestone hill clusters. Only dry shells specimens were found during the surveys.

####### Etymology.

The specific epithet honours Mr. Abang Arabi Abang Aimran, Chief Wildlife Warden of Sarawak Forestry Corporation, who has contributed significantly to the conservation of wildlife in Sarawak.

###### Microcystina atoni sp. nov.

Taxon classificationAnimaliaStylommatophoraAriophantidae

BA83972B-67E7-52B5-831D-F56843B3FB51

http://zoobank.org/324D05A6-F0D5-43C4-99C2-1217B79149A9

Figures 23B , 25A–F

####### Material examined.

***Holotype*** (SH 1.38 mm, SW 2.46 mm) (MZU.MOL.20.10), Malaysia, Sarawak, Kuching Division, Gunung Batu, limestone outcrop along Skio road, Jambusan, 2.4 miles E Bau, 1°23'50.65"N, 110°11'19.99"E, coll. M. E. Marzuki, 10.VII.2011. ***Paratypes***: 1 ex. (MZU.MOL.20.11), 1 ex. (ME0009903), same data as the holotype.

####### Differential diagnosis.

It is similar to *Microcystina chionodiscus* Vermeulen, 1996 and *Microcystina striatula* Vermeulen, Liew & Schilthuizen, 2015 in terms of the shell shape, but does not have the spiral striations on the protoconch and teleoconch. It is also different from *Microcystina microrhynchus* Vermeulen, Liew & Schilthuizen, 2015 and *Microcystina kilat* sp. nov. in lacking shell grooves and radial sculptures, and with an open but narrow umbilicus.

**Figure 25.**
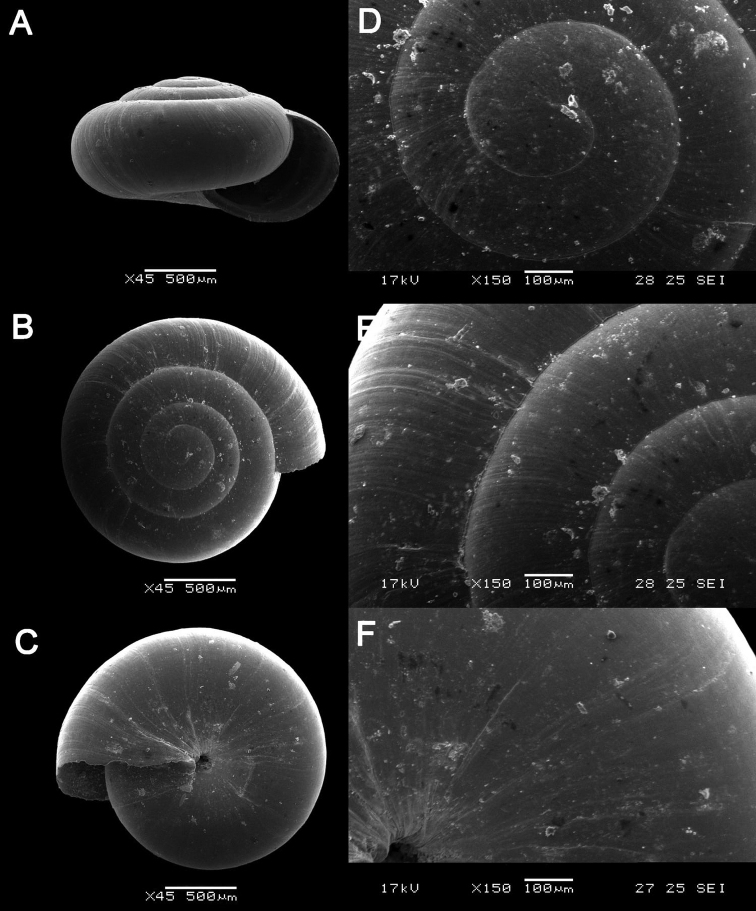
*Microcystina atoni* sp. nov. **A–F**MZU.MOL.20.10 Holotype **A** apertural view **B** apical view **C** basal view **D** enlargement of the apical side showing the apex **E** enlargement of the teleoconch showing the shell sculpture **F** enlargement of the basal side of the shell.

####### Description.

Shell very small, thin, translucent, white, lenticular, spire almost flat or slightly elevated. Surface with a slightly silky lustre. Whorls slightly convex. Number of whorls < 4. Protoconch dull, without spiral and radial threads. Teleoconch with spiral sculpture absent. Radial sculpture teleoconch: growth lines very fine, inconspicuous. Aperture lunulate. Peristome simple; somewhat thickened and reflected on columellar side, not thickened nor reflected on basal and palatal side. Umbilicus open, narrow; columellar wall very thick; umbilical region concave. Dimensions: shell height < 1.38 mm; shell width < 2.46 mm; diameters of the first three whorls 0.50 mm, 1.00 mm, and 1.58 mm, respectively; aperture height < 1.00 mm; aperture width < 1.25 mm.

####### Geographic distribution and habitat.

It is known from the Bau limestone hill clusters only. Only dry shells were found during the surveys.

####### Etymology.

The specific epithet honours Mr. Zolkipli Mohamad Aton, Chief Executive Officer of Sarawak Forestry Corporation, Controller of Wildlife and Controller of National Parks and Nature Reserves, who has contributed significantly to the conservation of wildlife in Sarawak.

###### Microcystina kilat sp. nov.

Taxon classificationAnimaliaStylommatophoraAriophantidae

62FEEBCF-EDBF-5D4C-BF03-6EA4FBB95D2C

http://zoobank.org/0C722321-FA76-41D5-B4C5-FB28A192013A

Figures 23C , 26A–F

####### Material examined.

***Holotype*** (SH 1.33 mm, SW 2.42 mm) (MZU.MOL.20.18), Malaysia, Sarawak, Kuching Division, Lobang Angin (Site 2), limestone outcrop near Sungai Sarawak Kanan, 1.75 miles W of Bau, 1°24'51.01"N, 110°8'13.48"E, coll. M. E. Marzuki, 16.IV.2017. ***Paratypes***: 1 ex. (ME0009430), same data as Holotype; 5 ex. (ME0009898), Bukit Sokwang (Site 2), northern site of Gunung Doya, limestone hill along Skio road, 2.05 miles E Bau, 1°23'45.69"N, 110°10'35.04"E, coll. M. E. Marzuki, 22.IV.2017; 1 ex. (ME0001764), Lobang Angin (Site 1), limestone outcrop near Sungai Sarawak Kanan, 1.75 miles W of Bau, 1°24'48.14"N, 110°8'12.21"E, coll. M. E. Marzuki, 11.III.2011; 1 ex. (ME0009273), Lobang Angin (Site 3), limestone outcrop near Sungai Sarawak Kanan, 1.75 miles W of Bau, 1°24'54.96"N, 110°8'13.62"E, coll. M. E. Marzuki, 23.IV.2017; 5 ex. (ME0001829), Fairy Caves (Site 1), south part of Gunung Kapor, 4 miles SW Bau, 1°22'53.76"N, 110°7'4.34"E, coll. M. E. Marzuki, 11.III.2011; 2 ex. (ME0009895), the same locality, coll. M. E. Marzuki, 23.VI.2010; 6 ex. (ME0008088), the same locality, coll. M. E. Marzuki, 17.IX.2016; 1 ex. (ME0010477), the same locality, coll. M. E. Marzuki, 7.VIII.2008; 1 ex. (ME0009902), the same locality, coll. M. E. Marzuki, 8.IV.2017; >10 ex. (ME0009899), Fairy Caves (Site 2), south part of Gunung Kapor, 4 miles SW Bau, 1°22'56.09"N, 110°6'58.82"E, coll. M. E. Marzuki, 8.IV.2017; 9 ex. (ME0009646), the same locality, coll. M. E. Marzuki, 7.I.2018; 24 ex. (ME0009001), Buddha Caves (Site 3), north part of Gunung Kapor, 3 miles SW Bau, 1°23'26.51"N, 110°7'10.02"E, coll. M. E. Marzuki, 9.IV.2017; 2 ex. (ME0009897), Bukit Sokwang (Site 1), northern site of Gunung Doya, limestone hill along Skio road, 2.05 miles E Bau, 1°23'52.11"N, 110°10'27.93"E, coll. M. E. Marzuki, 22.IV.2017; 4 ex. (ME0008943), Bukit Sokwang (Site 3), northern site of Gunung Doya, limestone hill along Skio road, 2.05 miles E Bau, 1°23'49.87"N, 110°10'32.14"E, coll. M. E. Marzuki, 22.IV.2017; 2 ex. (MZU.MOL.20.19), >10 ex. (ME0009904), Gunung Batu, limestone hill along Skio road, Jambusan, 2.4 miles E Bau, 1°23'50.65"N, 110°11'19.99"E, coll. M. E. Marzuki, 10.VII.2011; 1 ex. (ME0001767), the same locality, coll. M. E. Marzuki, 23.VI.2010; 10 ex. (ME0001699), Limestone escarpment near Kampung Benuk, 8.2 miles SW Kota Padawan, 1°18'41.43"N, 110°17'32.03"E, coll. M. E. Marzuki, 20.VIII.2008; 8 ex. (ME0001770), small limestone outcrop at Kampung Beratok, Serian-Kuching road, 14.3 miles NW Serian, 1°18'41.05"N, 110°24'37.13"E, coll. M. E. Marzuki, 21.VI.2010; 7 ex. (ME0006997), Gua Raya, along Kampung Skuduk-Chupak, 8.8 miles SE Siburan, 1°14'23.29"N, 110°25'49.05"E, coll. M. E. Marzuki, 1.I.2016; 3 ex. (ME0009455), Serian Division, Gunung Storib, small northern peak of Gunung Silabor, 15 miles S Serian, 0°57'30.75"N, 110°30'3.00"E, coll. M. E. Marzuki, 22.IX.2017; 6 ex. (ME0001773), Gunung Suka, Limestone outcrop near Kampung Picsing, Tebakang-Tebedu road, 8.45 miles SW Serian, 1°8'5.08"N, 110°26'53.30"E, coll. M. E. Marzuki, 20.VI.2010.

**Figure 26.**
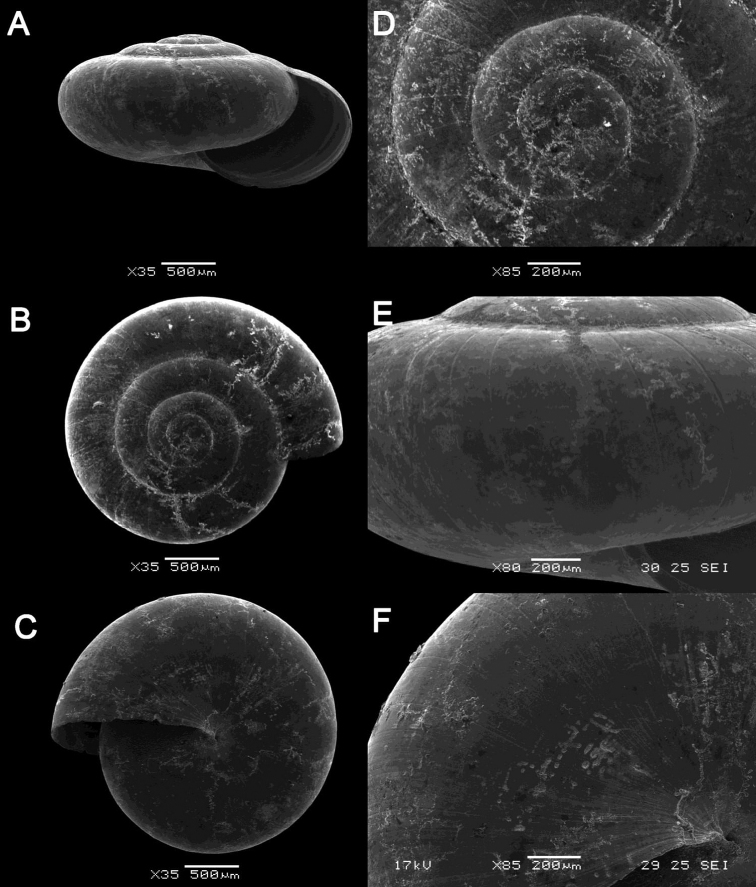
*Microcystina kilat*, sp. nov. **A–F**MZU.MOL.20.18 Holotype **A** apertural view **B** apical view **C** basal view **D** enlargement of the apical side showing the apex **E** enlargement of the body whorl showing the shell sculpture **F** enlargement of the basal side of the shell.

####### Differential diagnosis.

It differs from *Microcystina callifera* Vermeulen, Liew & Schilthuizen, 2015, in having a shell without spiral sculpture. The shell with an umbilicus that is entirely covered by a callus separates this new species from *Microcystina microrhynchus* Vermeulen, Liew & Schilthuizen, 2015 from Sabah.

####### Description.

Shell very small, thin, translucent, white, lenticular, spire almost flat or slightly elevated. Surface with shiny or a slightly silky lustre. Whorls slightly convex. Number of whorls < 4. Protoconch smooth, sometimes with a few inconspicuous, scattered radial grooves only. Teleoconch: spiral sculpture absent. Radial sculpture teleoconch growth lines inconspicuous, next to these inconspicuous to distinct, densely placed shallow grooves, often at irregular intervals. Aperture lunulate. Peristome simple; somewhat reflected on the columellar side, not thickened nor reflected on the basal and palatal side. Umbilicus entirely covered by callus; columellar wall thickened; umbilical region is moderately concave. Dimensions: shell height 1.33–1.85 mm; shell width 2.42–3.70 mm; diameters of the first three whorls 0.42 mm, 0.79 mm, and 1.50 mm, respectively; aperture height < 1.08 mm; aperture width < 1.25 mm.

####### Geographic distribution and habitat.

It is known from the Bau-Padawan limestone hill clusters. Only dry shells were found during the surveys.

####### Etymology.

From the Malay *kilat*, meaning shiny, in reference to the shell surface of the new species.

###### Microcystina lirata sp. nov.

Taxon classificationAnimaliaStylommatophoraAriophantidae

2BDF8C8E-440C-5CCB-A9B4-08EE094FD776

http://zoobank.org/9287C0EC-F61D-42AD-B349-875AFFCABAA4

Figures 23D , 27A–F

####### Material examined.

***Holotype*** (SH 0.77 mm, SW 1.35 mm) (MZU.MOL.20.14), Malaysia, Sarawak, Kuching Division, Buddha Caves (Site 3), north part of Gunung Kapor, 3 miles SW Bau, 1°23'26.51"N, 110°7'10.02"E, coll. M. E. Marzuki, 9.IV.2017. ***Paratypes***: 2 ex. (MZU.MOL.20.15), >10 ex. (ME0009214), same data as the holotype; 1 ex. (ME0006721), small limestone escarpment near Kampung Padang Pan, 15 miles SW Bau, 1°19'24.07"N, 110°3'46.34"E, coll. M. E. Marzuki, 27.IX.2015; 2 ex. (ME0008774), Bukit Sokwang (Site 3), northern site of Gunung Doya, limestone hill along Skio road, 2.05 miles E Bau, 1°23'49.87"N, 110°10'32.14"E, coll. M. E. Marzuki, 22.IV.2017; 4 ex. (ME0009166), Lobang Angin (Site 2), limestone outcrop near Sungai Sarawak Kanan, 1.75 miles W of Bau, 1°24'51.01"N, 110°8'13.48"E, coll. M. E. Marzuki, 16.IV.2017; 3 ex. (ME0008979), Fairy Caves (Site 1), south part of Gunung Kapor, 4 miles SW Bau, 1°22'53.76"N, 110°7'4.34"E, coll. M. E. Marzuki, 8.IV.2017; >10 ex. (ME0009235), Fairy Caves (Site 2), south part of Gunung Kapor, 4 miles SW Bau, 1°22'56.09"N, 110°6'58.82"E, coll. M. E. Marzuki, 8.IV.2017.

####### Differential diagnosis.

It differs from *Microcystina circumlineata* (Möllendorff, 1897) by having a smaller white shell with somewhat punctured-like secondary spiral grooves in between moderately spaced spiral threads.

####### Description.

Shell very small, rather thin, translucent, white, lenticular, spire moderately elevated. Surface with a silky lustre. Whorls slightly convex. Number of whorls < 4¼. Protoconch with a fine, moderately spaced spiral striation consisting of rows of minute, rather sharply outlined pits which are arranged in a cancellated pattern towards the teleoconch. Teleoconch: spiral sculpture present with very distinct, moderately spaced, continuous elevated spiral threads. In between these very fine, two rows of low, somewhat punctured-like secondary spiral grooves. Radial sculpture of teleoconch very fine as well as irregularly spaced growth lines. Periphery rounded; suture shallow. Aperture lunulate. Peristome simple; somewhat thickened and reflected on the columellar side, not thickened nor reflected on the basal and palatal side. Umbilicus open, narrow, partly covered by reflected peristome; umbilical region is moderately concave. Dimensions: shell height < 1.17 mm; shell width < 1.92 mm; diameters of the first three whorls 0.60 mm, 0.90 mm, and 1.37 mm, respectively; aperture height < 0.83 mm; aperture width < 0.83 mm.

####### Geographic distribution and habitat.

It is known from the Bau limestone hill clusters only. Only dry shells were found during the surveys.

####### Etymology.

From the Latin *lirata*, in reference to the prominent spiral sculpture of the shell.

**Figure 27.**
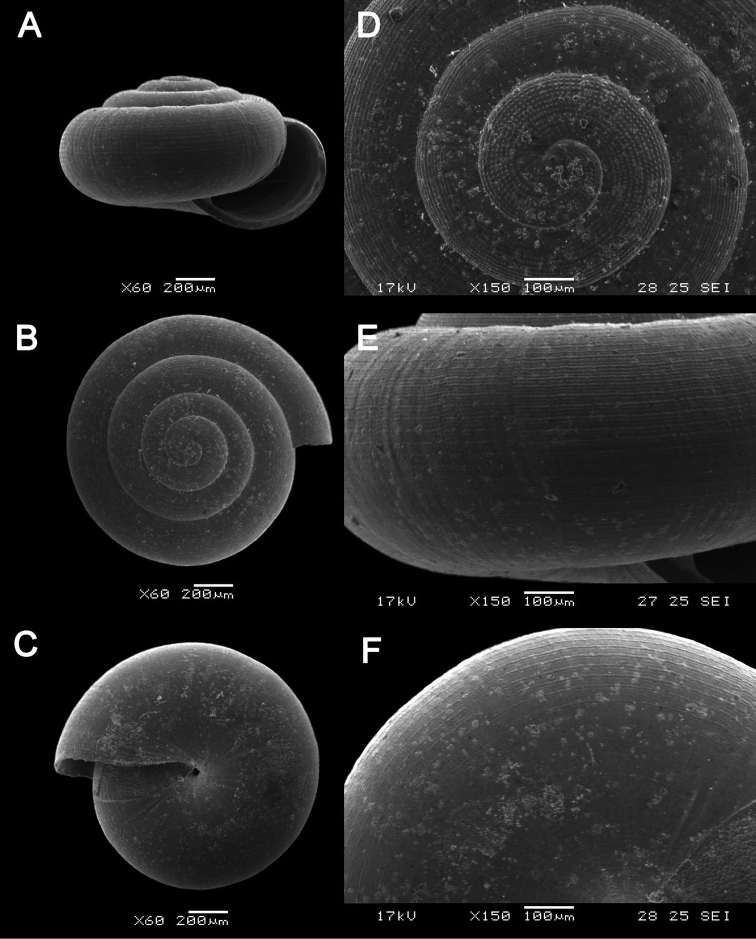
*Microcystina lirata*, sp. nov. **A–F**MZU.MOL.20.14 Holotype **A** apertural view **B** apical view **C** basal view **D** enlargement of the apical side showing the apex **E** enlargement of the body whorl showing the shell sculpture **F** enlargement of the basal side of the shell.

###### Microcystina oswaldbrakeni sp. nov.

Taxon classificationAnimaliaStylommatophoraAriophantidae

8833415D-777C-59B6-96FF-4D990F48EBB9

http://zoobank.org/F4562288-C1BF-43A2-B1C5-F11DC97F56B5

Figures 28A , 29A–F

####### Material examined.

***Holotype*** (SH 1.79 mm, SW 1.99 mm) (MZU.MOL.20.16), Malaysia, Sarawak, Kuching Division, Bukit Sokwang (Site 2), northern site of Gunung Doya, limestone hill along Skio road, 2.05 miles E Bau, 1°23'45.69"N, 110°10'35.04"E, coll. M. E. Marzuki, 22.IV.2017. ***Paratypes***: 2 ex. (MZU.MOL.20.17), >10 ex. (ME0002268), same data as the holotype; 6 ex. (ME0001758), Gunung Batu, limestone outcrop along Skio road, Jambusan, 2.4 miles E Bau, 1°23'50.65"N, 110°11'19.99"E, coll. M. E. Marzuki, 23.VI.2010; 1 ex. (ME0003275), the same locality, coll. M. E. Marzuki, 10.VII.2011; 4 ex. (ME0008818), the same locality, coll. M. E. Marzuki, 10.II.2017; 1 ex (ME0010295), Gua Tupap, Northern Gunung Batu Complex, Jambusan, 2.51 miles E Bau, Kuching Division, 1°24'21.25"N, 110°11'21.70"E, coll. M. E. Marzuki, 26.XII.2018; >10 ex. (ME0001751), Gunung Doya, limestone hill near Sungai Sebuyoh, 3.4 miles SE Bau, 1°22'57.24"N, 110°11'39.42"E, coll. M. E. Marzuki, 10.VII.2011; 3 ex (ME0007194), Fairy Caves (Site 1), south part of Gunung Kapor, 4 miles SW Bau, 1°22'53.76"N, 110°7'4.34"E, coll. M. E. Marzuki, 17.IX.2016; 1 ex. (ME0010478), the same locality, coll. M. E. Marzuki, 10.II.2017; 1 ex. (ME0011473), Fairy Caves (Site 2), south part of Gunung Kapor, 4 miles SW Bau, 1°22'56.09"N, 110°6'58.82"E, coll. M. E. Marzuki, 8.IV.2017; 2 ex. (ME0008012), Gunung Sebayat, limestone hill near Bengoh resettlement scheme, along Jambusan-Semadang road, 10 miles SE Bau, 1°18'24.54"N, 110°15'21.80"E, coll. M. E. Marzuki, 13.IX.2016; 10 ex. (ME0001796), Gunung Suka, Limestone outcrop near Kampung Picsing, Tebakang-Tebedu road, 8.45 miles SW Serian, 1°8'5.08"N, 110°26'53.30"E, coll. M. E. Marzuki, 20.VI.2010.

**Figure 28.**
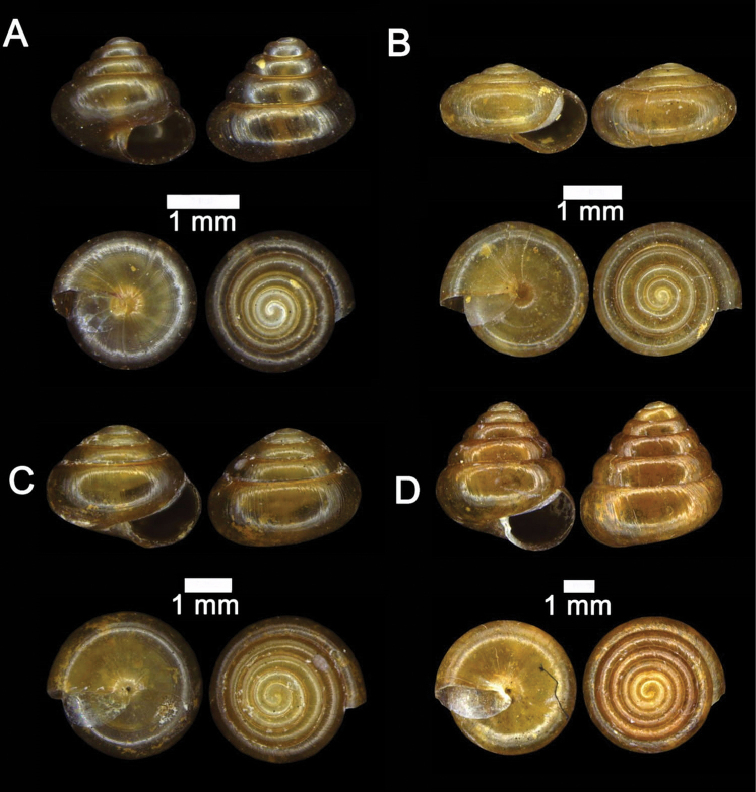
**A***Microcystina oswaldbrakeni*, sp. nov., MZU.MOL.20.17 Paratype Gunung Batu **B***Microcystina paripari*, sp. nov., ME 1746 Paratype Gunung Batu **C***Microcystina physotrochus* Vermeulen, Liew & Schilthuizen, 2015 ME 9072 Gunung Kapor **D***Microcystina seclusa* Godwin-Austen, 1891 ME 8153 Gunung Kapor.

####### Differential diagnosis.

It differs from *Microcystina seclusa* Godwin-Austen, 1891 in having a smaller shell with a silky surface that is covered by well-spaced faint spiral grooves and a closed umbilicus. This species also similar to *Microcystina arabii* sp. nov., see Remarks under that species.

####### Description.

Shell very small, thin, translucent, brown; conical-ovoid with convex sides, spire elevated, apex rounded. Surface with a glossy lustre. Whorls convex, rounded or slightly angular. Number of whorls < 5½. Protoconch with very fine, moderately spaced spiral striation consisting of rows of minute spiral grooves crossed by inconspicuous radial riblets towards the teleoconch. Teleoconch: spiral sculpture obsolete or with densely spaced, continuous, inconspicuous, narrow spiral grooves above the periphery but rather well-spaced below the periphery. Radial sculpture inconspicuous, oblique, widely but irregularly spaced, growth lines. Aperture lunulate. Peristome simple; somewhat reflected on columellar side, not thickened nor reflected on basal and palatal side. Umbilicus open, narrow, partly, or almost closed by reflected peristome; umbilical region is moderately concave. Dimensions: shell height < 1.79 mm; shell width < 1.99 mm; diameters of the first three whorls 0.42 mm, 0.74 mm, and 1.05 mm, respectively; aperture height < 0.73 mm; aperture width < 1.05 mm.

**Figure 29.**
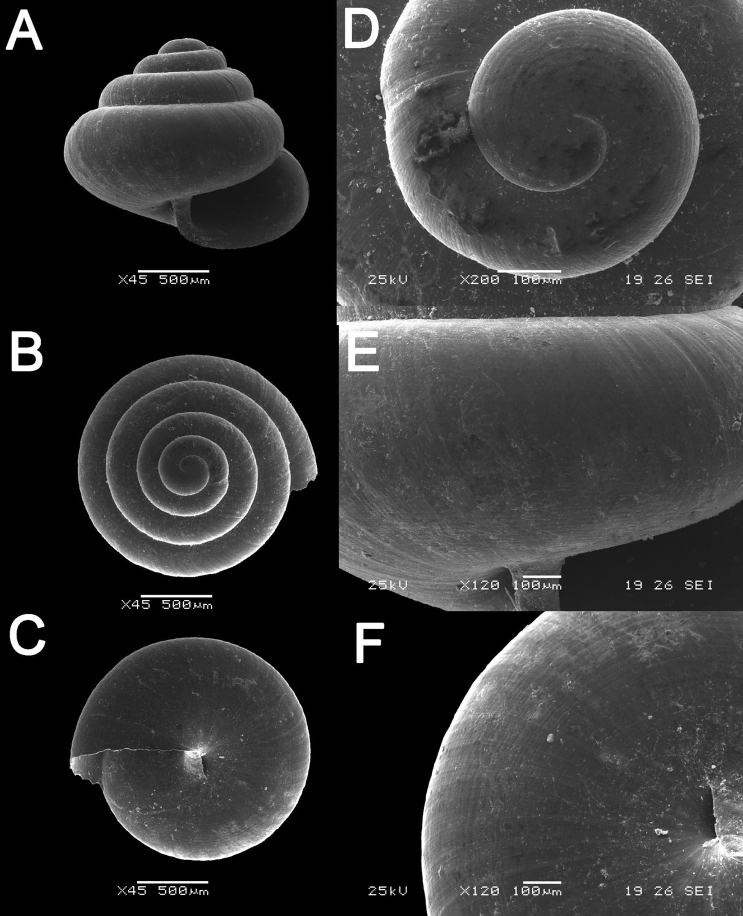
*Microcystina oswaldbrakeni*, sp. nov. **A–F**MZU.MOL.20.16 Holotype **A** apertural view **B** apical view **C** basal view **D** enlargement of the apical side showing the apex, **E** enlargement of the teleoconch showing the shell sculpture **F** enlargement of the basal side of the shell.

####### Geographic distribution and habitat.

It is known from the Bau and Serian-Padawan limestone hill clusters. Only dry shells were found during the surveys.

####### Etymology.

The specific epithet honours Mr. Oswald Braken Tisen, Deputy Chief Executive Officer of Sarawak Forestry Corporation, who has contributed significantly to the conservation of wildlife in Sarawak.

###### Microcystina paripari sp. nov.

Taxon classificationAnimaliaStylommatophoraAriophantidae

8C7D39D3-3B91-56B6-87C3-F20647AA3A7D

http://zoobank.org/28BC9C5A-A013-4B23-976B-E519B9ED15D0

Figures 28B , 30A–F

####### Material examined.

***Holotype*** (SH 1.25 mm, SW 2.08 mm) (MZU.MOL.20.12), Malaysia, Sarawak, Kuching Division, Fairy Caves (Site 2), south part of Gunung Kapor, 4 miles SW Bau, 1°22'56.09"N, 110°6'58.82"E, coll. M. E. Marzuki, 7.I.2018. ***Paratypes***: 2 ex. (MZU.MOL.20.13), 7 ex. (ME0009647), same data as the holotype; >10 ex. (ME0009329), the same locality, coll. M. E. Marzuki, 8.IV.2017; 1 ex. (ME0003845), Fairy Caves (Site 1), south part of Gunung Kapor, 4 miles SW Bau, 1°22'53.76"N, 110°7'4.34"E, coll. M. E. Marzuki, 11.III.2011; 2 ex. (ME0009469), the same locality, coll. M. E. Marzuki, 8.IV.2017; 2 ex. (ME0008510), the same locality, coll. M. E. Marzuki, 10.II.2017; 2 ex. (ME0001761), the same locality, coll. M. E. Marzuki, 23.VI.2010; 1 ex. (ME0001749), Gunung Doya, limestone hill near Sungai Sebuyoh, 3.4 miles SE Bau, 1°22'57.24"N, 110°11'39.42"E, coll. M. E. Marzuki, 10.VII.2011; 1 ex. (ME0009677), Bukit Sokwang (Site 3), northern site of Gunung Doya, limestone hill along Skio road, 2.05 miles E Bau, 1°23'49.87"N, 110°10'32.14"E, coll. M. E. Marzuki, 7.I.2018; 37 ex. (ME0009044), Buddha Caves (Site 3), north part of Gunung Kapor, 3 miles SW Bau, 1°23'26.51"N, 110°7'10.02"E, coll. M. E. Marzuki, 9.IV.2017; 1 ex. (ME0000783), Gunung Batu, limestone outcrop along Skio road, Jambusan, 2.4 miles E Bau, 1°23'50.65"N, 110°11'19.99"E, coll. M. E. Marzuki, 11.III.2011; >10 ex. (ME0001746), same locality, coll. M. E. Marzuki, 10.VII.2011.

####### Differential diagnosis.

It differs from *Microcystina muscorum* Van Benthem-Jutting, 1959 and *Microcystina gratilla* Van Benthem-Jutting, 1950 in having a shell without spiral striations on both the protoconch and teleoconch.

####### Description.

Shell very small, thin, translucent, straw yellow to brown, lenticular, spire moderately elevated. Surface with a glossy lustre. Whorls slightly convex. Number of whorls < 4. Protoconch smooth, sometimes with a few inconspicuous, corrugation at the suture. Teleoconch without spiral sculpture. Radial sculpture on teleoconch: inconspicuous growth lines, then next to these with distinct, well-spaced to densely spaced, shallow grooves, sometimes the latter striation is predominant. Aperture lunulate. Peristome simple; somewhat thickened and reflected on columellar side, not thickened nor reflected on basal and palatal side. Umbilicus open, narrow; umbilical region is moderately concave. Dimensions: shell height < 1.25 mm; shell width < 2.08 mm; diameters of the first three whorls 0.13 mm, 0.21 mm, and 0.25 mm, respectively; aperture height < 0.75 mm; aperture width < 0.92 mm.

####### Geographic distribution and habitat.

It is known from the Bau limestone hill clusters only. Only dry shells were found during the surveys.

####### Etymology.

The specific epithet *paripari* is in reference to the type locality, Gua Pari-pari, and is the Malay word for fairies.

**Figure 30.**
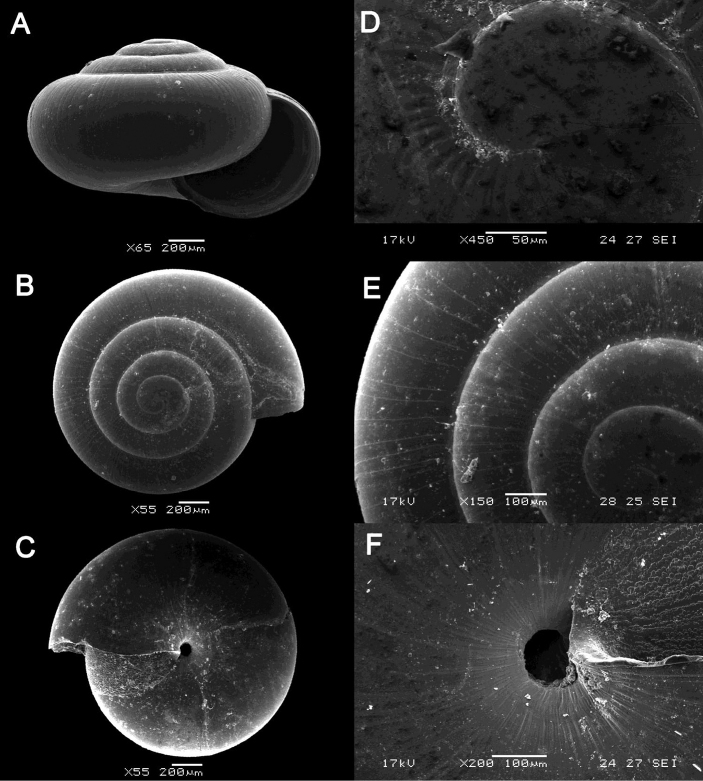
*Microcystina paripari*, sp. nov. **A–F**MZU.MOL.20.12 Holotype **A** apertural view **B** apical view **C** basal view **D** enlargement of the apical side showing the apex **E** enlargement of the teleoconch showing the shell sculpture **F** enlargement of the basal side of the shell.

###### Microcystina physotrochus

Taxon classificationAnimaliaStylommatophoraAriophantidae

Vermeulen, Liew & Schilthuizen, 2015

C75AC108-8739-5CAD-9066-10ED71D4ED64

Figure 28C

Microcystina physotrochus Vermeulen et al., 2015: 57–59, fig. 37A–C.

####### Type locality.

“Malaysia, Sabah, Sandakan Province, Kinabatangan Valley, Batu Keruak 2, near Sukau”.

####### Material examined.

Gunung Doya: ME 8953. Gunung Kapor: ME 2262, ME 2264, ME 2265, ME 2266, ME 9072, ME 9098, ME 9256. Lobang Angin: ME 9174. Gunung Batu: ME 2263.

####### Distribution in Borneo.

Sarawak: Kuching, Bintulu, and Miri divisions. Enemic to Borneo.

####### Remarks.

Living snails were observed foraging among leaf litter and plant debris near the cliff in a lowland limestone forest.

###### Microcystina seclusa

Taxon classificationAnimaliaStylommatophoraAriophantidae

Godwin-Austen, 1891

FE1ABB60-A026-5794-B38E-9ABCD3657227

Figure 28D

Microcystina seclusa Godwin-Austen, 1891: 38.

####### Type locality.

“Borneo, cave-earth”.

####### Material examined.

Gunung Doya: ME 8933, ME 8954. Gunung Kapor: ME 8153, ME 9209. Gunung Batu: ME 0439, ME 2261.

####### Distribution in Borneo.

Sarawak: Kuching Division. Endemic to Borneo.

####### Remarks.

Only dry shells were found during the surveys. It differs from *Microcystis bunguranensis* Smith, 1894 from Natuna Island by having a larger and higher spire shell (Smith 1894b).

##### *Vitrinula* Gray, 1857

###### Vitrinula glutinosa

Taxon classificationAnimaliaStylommatophoraAriophantidae

(Metcalfe, 1851)

3A9A45F3-6B4B-5D91-BD7D-C25D92AD1E74

Figures 22F , 51D

Helix glutinosa Metcalfe, 1851: 70–71.

####### Type locality.

“Borneo”.

####### Material examined.

Gunung Doya: ME 1620, ME 8915, ME 9114. Gunung Kapor: ME 1617, ME 1618, ME 1619, ME 8080, ME 8508, ME 8782, ME 8967, ME 9407. Kampung Padang Pan: ME 6675. Lobang Angin: ME 8728, ME 8750, ME 9023. Gunung Batu: ME 1621, ME 4785, ME 4983, ME 8832.

####### Distribution in Borneo.

Sarawak: Kuching, Sibu, Mukah, Kapit, and Miri divisions. Kalimantan: West Kalimantan Province. Endemic to Borneo.

####### Remarks.

This species exhibits high variability in shell form, ranging from high to low spire and in colour from pale to dark brown.

#### Family Camaenidae Pilsbry, 1895

##### *Bradybaena* Beck, 1837

###### Bradybaena similaris

Taxon classificationAnimaliaStylommatophoraCamaenidae

(Férussac, 1822)

920A00B4-9CC0-574C-8742-6741E83FB0A1

Figure 31A

Helix (Helicigona) similaris Férussac, 1822: 43.

####### Type locality.

“Timor”, Indonesia.

####### Material examined.

Bukit Sekunyit: ME 4905. Gunung Batu: ME 4906, ME 8828.

####### Distribution in Borneo.

Sabah: Interior, West Coast, Kudat and Sandakan divisions. Sarawak: Kuching, Samarahan, Sibu, Mukah, Kapit, and Miri divisions. ***Distribution elsewhere.*** Southeast mainland Asia to Indo-Australian archipelago, South America (Reeve 1851; Vermeulen and Whitten 1998).

####### Remarks.

Probably an introduced species. The species is known only from disturbed habitats.

**Figure 31.**
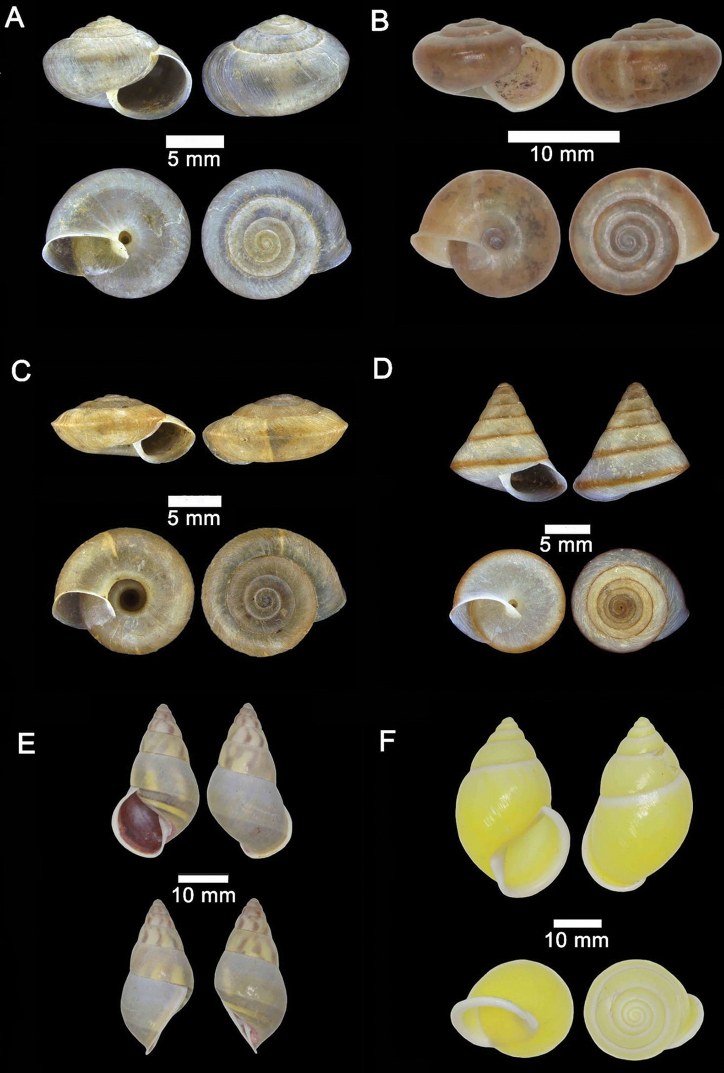
**A***Bradybaena similaris* (Férussac, 1821) ME 4906 Gunung Batu **B***Chloritis tomentosa* (L. Pfeiffer, 1854) ME 8917 Gunung Doya **C***Landouria winteriana* (L. Pfeiffer, 1842) ME 8078 Gunung Kapor **D***Ganesella acris* (Benson, 1859) ME 8963 Gunung Kapor **E***Amphidromus angulatus* Fulton, 1896 ME 4632 Gunung Kapor **F**Amphidromus cf. similis Pilsbry, 1900 ME 8756 Gunung Kapor.

##### *Chloritis* Beck, 1837

###### Chloritis tomentosa

Taxon classificationAnimaliaStylommatophoraCamaenidae

(L. Pfeiffer, 1854)

C0B950F5-034A-5D74-8C48-D1501BCA0DC8

Figure 31B

Helix tomentosa L. Pfeiffer, 1854a: 289–290.

####### Type locality.

“Sarawak, Borneo”.

####### Material examined.

Gunung Doya: ME 8917, ME 9164. Gunung Kapor: ME 1532, ME 1549, ME 8077, ME 9266. Kampung Padang Pan: ME 6683. Gunung Batu: ME 1540, ME 1547, ME 1548.

####### Distribution in Borneo.

Sarawak: Kuching Division. Labuan: Kuraman Island. ***Distribution elsewhere.*** Sumatra (Bock 1881; Van Benthem-Jutting 1959).

####### Remarks.

Only dry shells were found during the surveys. This species is different from *Bradybaena similaris* (Férussac, 1822) in having very fine hair pits that cover the shell surfaces and a slightly angular peristome between the columellar and basal sides.

##### *Landouria* Godwin-Austen, 1918

###### Landouria winteriana

Taxon classificationAnimaliaStylommatophoraCamaenidae

(L. Pfeiffer, 1842)

902D2E2F-F842-5B72-B270-7B060C895550

Figure 31C

Helix winteriana Pfeiffer In Philippi, 1843: 23, pl. 2, fig. 7.

####### Type locality.

“Java”.

####### Material examined.

Bukit Sekunyit: ME 1580. Gunung Kapor: ME 1579, ME 1583, ME 1584, ME 5979, ME 8078, ME 8778, ME 9226. Lobang Angin: ME 9274.

####### Distribution in Borneo.

Sarawak: Kuching and Miri divisions. ***Distribution elsewhere.*** Indo-Australian archipelago (Vermeulen and Whitten 1998).

####### Remarks.

The shells from Bau are similar to the syntype of *Plectotropis kraepelini* Leschke, 1914, ZMH 98416 [= *Landouria winteriana* (Pfeiffer, 1842)]. For further details, see Nurinsiyah et al. (2019: 10–17)

##### *Ganesella* W. T. Blanford, 1863

###### Ganesella acris

Taxon classificationAnimaliaStylommatophoraCamaenidae

(Benson, 1859)

0C3EFB2B-26DD-5DF0-AA32-8C08ED1EBD44

Figures 31D , 53B

Helix acris Benson, 1859: 387–388.

####### Type locality.

“Teria Ghát montium Khasiæ” [= Khasi Hills, Teria Ghat, India].

####### Material examined.

Gunung Doya: ME 8916. Gunung Kapor: ME 1561, ME 1562, ME 1564, ME 1567, ME 1570, ME 8512, ME 8773, ME 8963, ME 9041. Kampung Padang Pan: ME 6682. Lobang Angin: ME 8939, ME 8977. Gunung Batu: ME 1565.

####### Distribution in Borneo.

Sarawak: Kuching and Miri divisions. Sabah: Tawau, Sandakanm, and West Coast divisions. ***Distribution elsewhere.*** South to Southeast Asia mainland, Sumatra to Java (Benson 1859; Crosse 1879; Smith 1887; Van Benthem-Jutting 1959).

##### *Amphidromus* Albers, 1850

###### Amphidromus angulatus

Taxon classificationAnimaliaStylommatophoraCamaenidae

Fulton, 1896

3FB3DF60-C0B9-5119-8E16-B13591239693

Figure 31E

Amphidromus angulatus Fulton, 1896: 84–85, pl. 6, fig. 3.

####### Type locality.

“Sarawak”.

####### Material examined.

Gunung Doya: ME 8919, ME 9176, ME 9191. Gunung Kapor: ME 4611, ME 4632, ME 8075, ME 8789, ME 9045, ME 9223. Lobang Angin: ME 4631. Gunung Batu: ME 4612, ME 4630.

####### Distribution in Borneo.

Sarawak: Kuching and Miri divisions. Endemic to Borneo.

####### Remarks.

Only dry shells were found during the surveys. This species is similar to *Amphidromus thalassochromus* Vermeulen & Junau, 2007 and *Amphidromus coeruleus* Clench & Archer, 1932 in terms of shell shape and colour pattern on the shell surface. However, it differs from *A. thalassochromus* by having a rounded last whorl at the periphery and it differs from *A. coeruleus* in having a somewhat obese shell with a short spire.

###### Amphidromus cf. similis

Taxon classificationAnimaliaStylommatophoraCamaenidae

Pilsbry, 1900

F65A076F-BFE2-5318-8737-AA9CE112D11B

Figures 31F , 53A

Amphidromus perversus form similis Pilsbry, 1900: 150, pl. 51, fig. 52.

####### Type locality.

“Sadong, West Sarawak”.

####### Material examined.

Gunung Doya: ME 8918, ME 8923. Gunung Kapor: ME 3724, ME 4160, ME 4595, ME 4596, ME 4597, ME 5970, ME 8076, ME 8752, ME 8756, ME 9242. Gunung Batu: ME 4599.

####### Distribution in Borneo.

Sarawak: Kuching Division. Endemic to Borneo.

####### Remarks.

The shells from Bau are different from Pilsbry’s *Amphidromus similis* in having a shell with a translucent white callus and parietal wall.

#### Family Chronidae Thiele, 1931

##### *Kaliella* W. T. Blanford, 1863

###### Kaliella barrakporensis

Taxon classificationAnimaliaStylommatophoraChronidae

(L. Pfeiffer, 1852)

AB69B9A8-81DB-56EE-996D-4CD26716C398

Figures 32A , 52C

Helix barrakporensis L. Pfeiffer, 1852: 156.

####### Type locality.

“Barrakpore, Indiæ” [= Barrackpore, West Bengal, India].

####### Material examined.

Gunung Doya: ME 8931, ME 8951, ME 9034. Gunung Kapor: ME 1910, ME 1926, ME 8156, ME 8787, ME 9009, ME 9026. Gunung Stulang: ME 5896. Kampung Padang Pan: ME 6678. Lobang Angin: ME 8986, ME 9021, ME 9268. Gunung Batu: ME 1912, ME 8813.

####### Distribution in Borneo.

Sarawak: Kuching, Serian, and Miri divisions. Sabah: Interior, Sandakan, Tawau, and West Coast divisions. Kalimantan (Vermeulen et al. 2015). ***Distribution elsewhere.*** Africa and South Asia mainland to Indo-Australian archipelago, Europe (Godwin-Austen 1882; Vermeulen and Whitten 1998; Preece and Naggs 2014).

**Figure 32.**
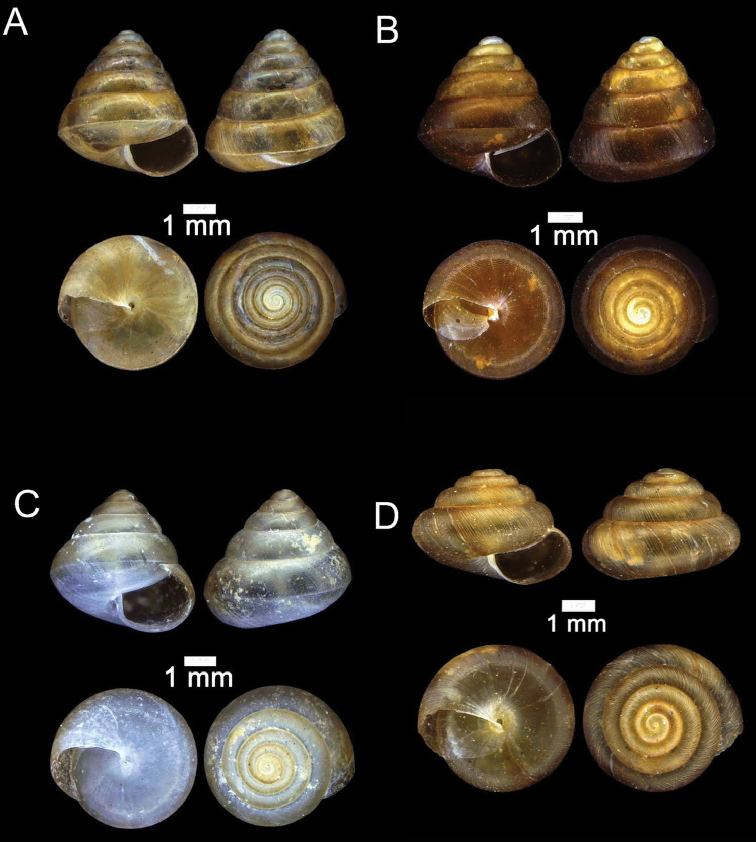
**A***Kaliella barrakporensis* (L. Pfeiffer, 1852) ME 9009 Gunung Kapor **B***Kaliella busauensis* (E. A. Smith, 1895) ME 1892 Gunung **C***Kaliella calculosa* (Gould, 1852) ME 1863 Gunung Kapor **D***Kaliella doliolum* (L. Pfeiffer, 1846) ME 9074 Gunung Kapor.

###### Kaliella busauensis

Taxon classificationAnimaliaStylommatophoraChronidae

(E. A. Smith, 1895)

502B29C2-FAEB-5CC0-A0F3-37B979CE659F

Figure 32B

Sitala busauensis E. A. Smith, 1895: 111, pl. 3, fig. 9.

####### Type locality.

“Busau, Sarawak” [= Jambusan Hills, Bau, Sarawak].

####### Material examined.

Gunung Sebayat: ME 8304. Gunung Doya: ME 9702, ME 8990. Gunung Kapor: ME 1880. Lobang Angin: ME 9259, ME 9264. Gunung Batu: ME 1868, ME 1873, ME 1892.

####### Distribution in Borneo.

Sarawak: Kuching Division. Endemic to Borneo.

####### Remarks.

Only dry shells were found during the surveys. It differs from other Bornean *Kaliella* species by having a higher dark brown spired shell with a cancellated shell surface due to the prominent spiral grooves and oblique radial riblets.

###### Kaliella calculosa

Taxon classificationAnimaliaStylommatophoraChronidae

(Gould, 1852)

39E5B6E7-3351-5ED5-90A8-700EA801ECAE

Figure 32C

Helix calculosa Gould, 1852: 48.

####### Type locality.

“Tahiti” [= Tahiti Island, French Polynesia].

####### Material examined.

Bukit Sekunyit: ME 1885. Gunung Doya: ME 1865, ME 8932, ME 8952, ME 8995. Gunung Kapor: ME 1863, ME 1898, ME 1911, ME 8973, ME 9049, ME 9238. Kampung Padang Pan: ME 6722. Lobang Angin: ME 8747, ME 9177, ME 9259. Gunung Batu: ME 1866, ME 8816.

####### Distribution in Borneo.

Sarawak: Kuching, Serian and Miri divisions. Sabah: Interior, Sandakan, Tawau, and West Coast divisions. ***Distribution elsewhere.*** South Asia mainland to Indo-Australian archipelago and Pacific Islands (Vermeulen et al. 2015).

####### Remarks.

The juvenile shell of this species is similar to *Kaliella barrakporensis* (Pfeiffer, 1852) and *K. busauensis* (Smith, 1895), but it differs from the two species by having a lower conical, brittle, whitish shell with moderately spaced spiral striae above the periphery.

###### Kaliella doliolum

Taxon classificationAnimaliaStylommatophoraChronidae

(L. Pfeiffer, 1846)

ABE58CBD-6205-5312-B3C7-A2720031E51A

Figure 32D

Helix doliolum L. Pfeiffer, 1846b: 41–42.

####### Type locality.

“Sibonga, island of Zebu” [= Sibonga, Cebu Island, Philippines].

####### Material examined.

Gunung Kapor: ME 1851, ME 1874, ME 1895, ME 9010, ME 9074, ME 9252.

####### Distribution in Borneo.

Sarawak: Kuching, Serian, and Miri divisions. Sabah: Interior, Kudat, Sandakan, Tawau, and West Coast divisions. ***Distribution elsewhere.*** Southeast Asia mainland to Indo-Australian archipelago and Pacific Islands (Vermeulen et al. 2015).

####### Remarks.

Living snails were observed foraging among leaf litter and plant debris near the cliffs in a lowland limestone forest.

###### Kaliella microconus

Taxon classificationAnimaliaStylommatophoraChronidae

(Mousson, 1865)

E8E9F4B4-8FBB-5502-B101-D9A4F0B97BFC

Figure 33A

Nanina microconus Mousson, 1865: 192.

####### Type locality.

“Lomma-Lomma (Viti)” [= Loma Loma, Fiji].

####### Material examined.

Bukit Sekunyit: ME 1884. Gunung Doya: ME 1877, ME 9703, ME 8934, ME 8959, ME 8996. Gunung Kapor: ME 1860, ME 1896, ME 1909, ME 8154, ME 8499, ME 8975, ME 9007, ME 9073. Kampung Padang Pan: ME 6677. Lobang Angin: ME 8738, ME 8987, ME 9146. Gunung Batu: ME 1855, ME 1893, ME 1913, ME 8817.

####### Distribution in Borneo.

Sarawak: Kuching, Serian, and Miri divisions. Sabah: Interior, Kudat, Sandakan, Tawau, and West Coast divisions. Kalimantan: South Kalimantan Province. ***Distribution elsewhere.*** South-east Asia to Australia and the Pacific Islands (Vermeulen and Whitten 1998).

####### Remarks.

Living snails were observed foraging among leaf litter and plant debris near the cliff in a lowland limestone forest.

**Figure 33.**
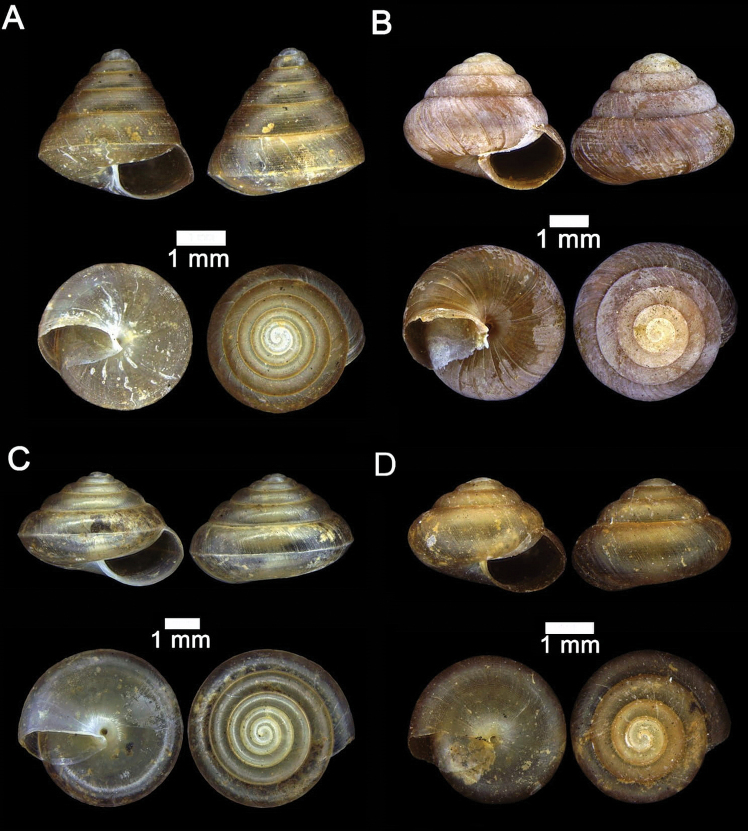
**A***Kaliella microconus* (Mousson, 1865) ME 1855 Gunung Batu **B***Kaliella micula* (Mousson, 1857) ME 9650 Gunung Kapor **C***Kaliella platyconus* (Möllendorff, 1897) ME 8814 Gunung Batu **D***Kaliella scandens* (Cox, 1871) ME 1928 Gunung Kapor.

###### Kaliella micula

Taxon classificationAnimaliaStylommatophoraChronidae

(Mousson, 1857)

549A03A1-1168-5D79-A700-02108FE18BBD

Figure 33B

Zonites micula Mousson, 1857: 158.

####### Type locality.

“Insula Balie” [= Bali Island, Indonesia].

####### Material examined.

Gunung Kapor: ME 9650.

####### Distribution in Borneo.

Sarawak: Kuching Division. ***Distribution elsewhere.*** Peninsular Malaysia to Lesser Sunda, Indonesia (Vermeulen and Whitten 1998).

####### Remarks.

Only dry shells were found during the surveys. It differs from *K. scandens* by having a larger shell with wider whorls that rapidly increase in size. For further details on the differences between this species and *K. dendrobates* (Tillier & Bouchet, 1989), see Vermeulen et al. (2015: 105).

###### Kaliella platyconus

Taxon classificationAnimaliaStylommatophoraChronidae

Möllendorff, 1897

28607C72-A168-5E3A-A202-A64DDCB5AD6E

Figure 33C

Kaliella platyconus Möllendorff, 1897b: 59.

####### Type locality.

“Java”, Indonesia.

####### Material examined.

Gunung Batu: ME 8814.

####### Distribution in Borneo.

Sarawak: Kuching and Samarahan divisions. ***Distribution elsewhere.*** Sumatra to Sumbawa, Indonesia (Vermeulen and Whitten 1998).

####### Remarks.

Living snails were observed foraging among leaf litter and plant debris near the cliff in a lowland limestone forest. The shells from Bau are the first record of this species in Borneo. This species is different from *Kaliella barrakporensis* (Pfeiffer, 1852) and *Kaliella accepta* (Smith, 1895) in having a low conical shell with wider whorls.

###### Kaliella scandens

Taxon classificationAnimaliaStylommatophoraChronidae

(Cox, 1871)

8AA2A5DE-AB80-5B55-BE41-EE8486D46090

Figure 33D

Helix scandens Cox, 1871: 645, pl. 52, fig. 5.

####### Type locality.

“Port Macquarie, east coast of Australia”.

####### Material examined.

Bukit Sekunyit: ME 1883. Gunung Doya: ME 1882, ME 8935, ME 9033, ME 9110. Gunung Kapor: ME 1897, ME 1928, ME 8155, ME 8500, ME 9008, ME 9025, ME 9075, ME 9486. Lobang Angin: ME 8985, ME 9202, ME 9277. Gunung Batu: ME 1875, ME 8835.

####### Distribution in Borneo.

Sarawak: Kuching, Serian, and Miri divisions. Sabah: Interior, Sandakan, Tawau, and West Coast divisions. Kalimantan: exact location was not mentioned in Vermeulen et al. (2015). ***Distribution elsewhere.*** South-east Asia to Australia and the Pacific Islands (Vermeulen et al. 2015).

#### Family Endodontidae Pilsbry, 1895

##### *Beilania* Preston, 1913

###### Beilania philippinensis

Taxon classificationAnimaliaStylommatophoraEndodontidae

(C. Semper, 1874)

7B1BBFF5-32B8-532E-8936-C68D4B2A21CF

Figure 34A

Endodonta philippinensis C. Semper, 1874: 140.

####### Type locality.

“Antipolo bei Manila, Luzon” [= Antipolo, Luzon Island, Philippines].

####### Material examined.

Gunung Batu: ME10290.

####### Distribution in Borneo.

Sarawak: Kuching and Miri divisions. Sabah: Tawau and West Coast divisions. ***Distribution elsewhere.*** Philippines, Java, Sulawesi to Timor, Indonesia (Solem 1957).

####### Remarks.

Only dry shells were found during the surveys.

**Figure 34.**
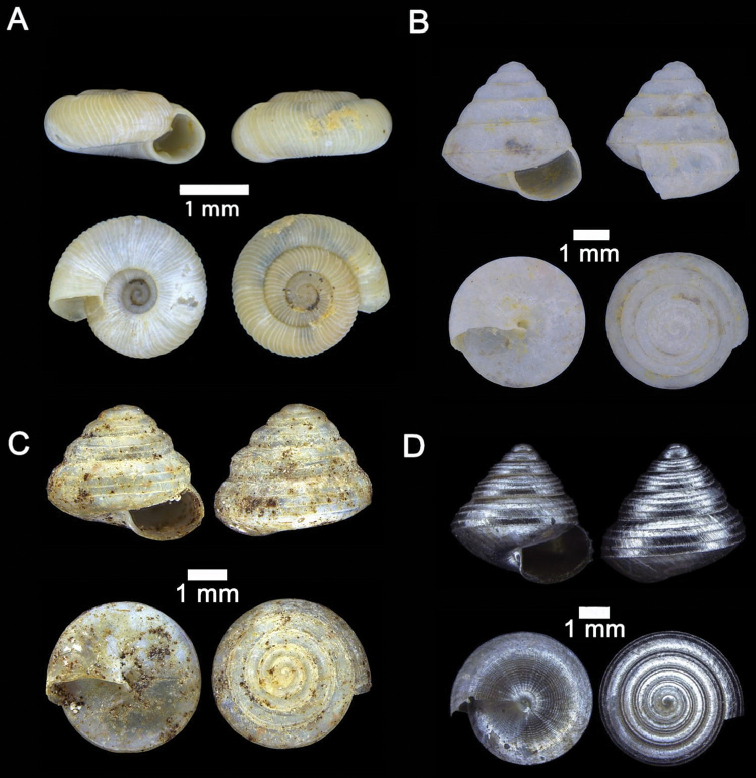
**A***Beilania philippinensis* (C. Semper, 1874) ME 10290 Gunung Batu **B***Philalanka jambusanensis*, sp. nov., ME 1879 Paratype Gunung Batu **C***Philalanka kusana* (Aldrich, 1889) ME 5897 Gunung Batu **D***Philalanka moluensis* (E. A. Smith, 1893) ME 0443 Gunung Doya [not in natural colour, shell surface coated with platinum for examination under scanning electron microscope].

##### *Philalanka* Godwin-Austen, 1898

###### Philalanka jambusanensis sp. nov.

Taxon classificationAnimaliaStylommatophoraCharopidae

E54780AF-9A13-5865-8425-60604226BAE3

http://zoobank.org/B64A15E6-362B-41D5-99A8-FFF1165E93D5

Figures 34B , 35A–E

####### Material examined.

***Holotype*** (SH 2.78 mm, SW 3.11 mm) (MZU.MOL.20.20), Malaysia, Sarawak, Kuching Division, Gunung Batu, limestone outcrop along Skio road, Jambusan, 2.4 miles E Bau, 1°23'50.65"N, 110°11'19.99"E, coll. M. E. Marzuki, 10.VII.2011. ***Paratypes***: 1 ex. (ME0001879), same data as holotype.

####### Differential diagnosis.

It differs from *Philalanka thienemanni* Rensch, 1932, by having a shell with spiral striations only on the first 1½ whorls above the peripheral thread and a narrowly open umbilicus. This species is different from *Philalanka micromphala* Van Benthem-Jutting, 1952 in having a high conical white shell with no spiral sculpture above the periphery.

####### Description.

Shell very small, dextral, thin, translucent, white; spire conical-ovoid. Surface with a shiny lustre. Whorls flat or with slightly convex sides, rounded. Number of whorls 4¾. Protoconch whorls convex with moderately spaced spiral striations consisting of 4–6 rows of inconspicuous spiral threads towards the teleoconch. Teleoconch with no spiral sculpture above periphery, well-spaced below periphery, fine spiral threads present except in the umbilical region. Radial sculpture on the teleoconch consisting of densely spaced, fine, slightly oblique growth lines. Last whorl with a distinct peripheral thread coinciding with the suture of the penultimate whorl. Aperture: peristome simple; somewhat reflected and thickened on columellar side, not thickened nor reflected on basal or palatal side. Umbilicus open, narrow; umbilical region moderately concave. Dimensions: shell height 2.78 mm; shell width 3.11 mm; diameters of the first three whorls 0.61 mm, 1.22 mm, and 1.78 mm, respectively; shell aperture height 1.11 mm; shell aperture width 1.67 mm.

**Figure 35.**
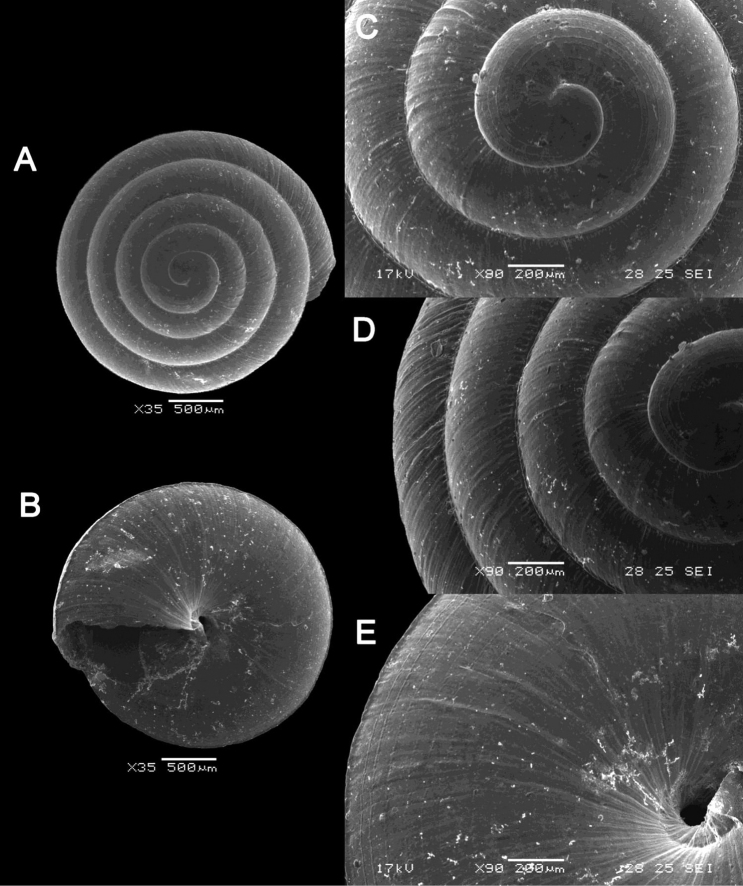
*Philalanka jambusanensis*, sp. nov. **A–F**MZU.MOL.20.20 Holotype. **A** Apical view **B** Basal view, **C** Enlargement of the apical side showing the apex **D** Enlargement of the teleoconch showing the shell sculpture **E** Enlargement of the basal side of the shell.

####### Geographic distribution and habitat.

It is only known from the type locality. Only dry shells were found during the surveys.

####### Etymology.

The specific epithet *jambusanensis* is from the name of Jambusan, where the specimens were found.

###### Philalanka kusana

Taxon classificationAnimaliaStylommatophoraCharopidae

(Aldrich, 1889)

242A49B2-5550-5FD4-840C-5D36448A49E7

Figure 34C

Trochomorpha kusana Aldrich, 1889: 24, pl. 3, figs 3, 3A, 3B.

####### Type locality.

“Kusan and Penggiron districts in South-eastern Borneo” [= Kusan and Pangeran in South Kalimantan, Indonesian Borneo].

####### Material examined.

Gunung Doya: ME 8956, ME 9115. Gunung Kapor: ME 2238, ME 2244, ME 2249, ME 8157, ME 8501, ME 8788, ME 9029. Gunung Stulang: ME 5897. Lobang Angin: ME 8737, ME 9181, ME 9270. Gunung Batu: ME 2252, ME 2254.

####### Distribution in Borneo.

Sarawak: Kuching, Serian, Kapit, and Miri divisions. Sabah: Interior, Kudat, Sandakan, Tawau, and West Coast divisions. Kalimantan: Exact location was not mentioned in Vermeulen et al. (2015). ***Distribution elsewhere.*** West Malaysia to Papua (Vermeulen et al. 2015).

####### Remarks.

Living snails were observed foraging among leaf litter and plant debris near the cliff in a lowland limestone forest.

###### Philalanka moluensis

Taxon classificationAnimaliaStylommatophoraCharopidae

(E. A. Smith, 1893)

6E4FEDFC-FC14-5E84-8FD1-0C5EE711C07D

Figure 34D

Sitala moluensis E. A. Smith, 1893: 343, pl. 25, fig. 4.

####### Type locality.

“Molu or Mulu Mountains, N. Borneo”.

####### Material examined.

Gunung Doya: ME 0443.

####### Distribution in Borneo.

Sarawak: Kuching and Miri divisions. Sabah: Interior, Sandakan, Tawau, and West Coast divisions. Endemic to Borneo.

####### Remarks.

Only dry shells were found during the surveys.

#### Family Punctidae Morse, 1864

##### *Paralaoma* Iredale, 1913

###### Paralaoma angusta

Taxon classificationAnimaliaStylommatophoraPunctidae

Vermeulen, Liew & Schilthuizen, 2015

B6DB4355-8B7C-5F5B-8BAC-B9DF6DAFF01F

Figures 36A , 37

Paralaoma angusta Vermeulen et al., 2015: 109, fig. 76A, B.

####### Type locality.

“Malaysia, Sabah, West Coast Province, Crocker Range, Kiansom Waterfall”.

####### Material examined.

Gunung Doya: ME 8927, ME 8945, ME 8994. Gunung Kapor: ME 1734, ME 1760, ME 1927, ME 8976, ME 9042, ME 9067, ME 9262, ME 9641. Kampung Bunga Rampai: ME 0741. Lobang Angin: ME 9265. Gunung Batu: ME 1756, ME 1763, ME 8836.

**Figure 36.**
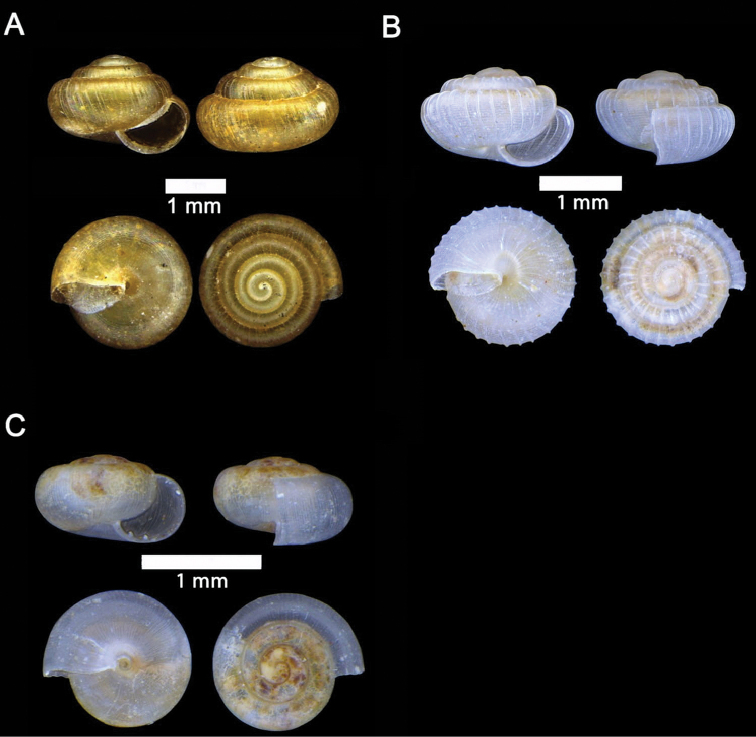
**A***Paralaoma angusta* Vermeulen, Liew & Schilthuizen, 2015 ME 9641 Gunung Kapor **B***Paralaoma sarawakensis*, sp. nov., ME 2269 Paratype Gunung Doya **C***Charopa* sp. “argos” ME 8593 Gunung Doya.

####### Distribution in Borneo.

Sarawak: Kuching and Serian divisions. Sabah: West Coast Division. Kalimantan: South Kalimantan Province. Endemic to Borneo.

####### Remarks.

This is the first record of this species in Sarawak. Living snails were observed foraging among leaf litter and plant debris near the cliffs in a lowland limestone forest. The shells from Bau are slightly larger and more obese than the shells from Sabah. Dimensions: Height < 1.65 mm; width < 2.37 mm; diameters of the first three whorls 0.41 mm, 0.93 mm, and 1.65 mm, respectively; number of whorls < 4½; aperture height < 0.93 mm; aperture width < 1.24 mm.

**Figure 37.**
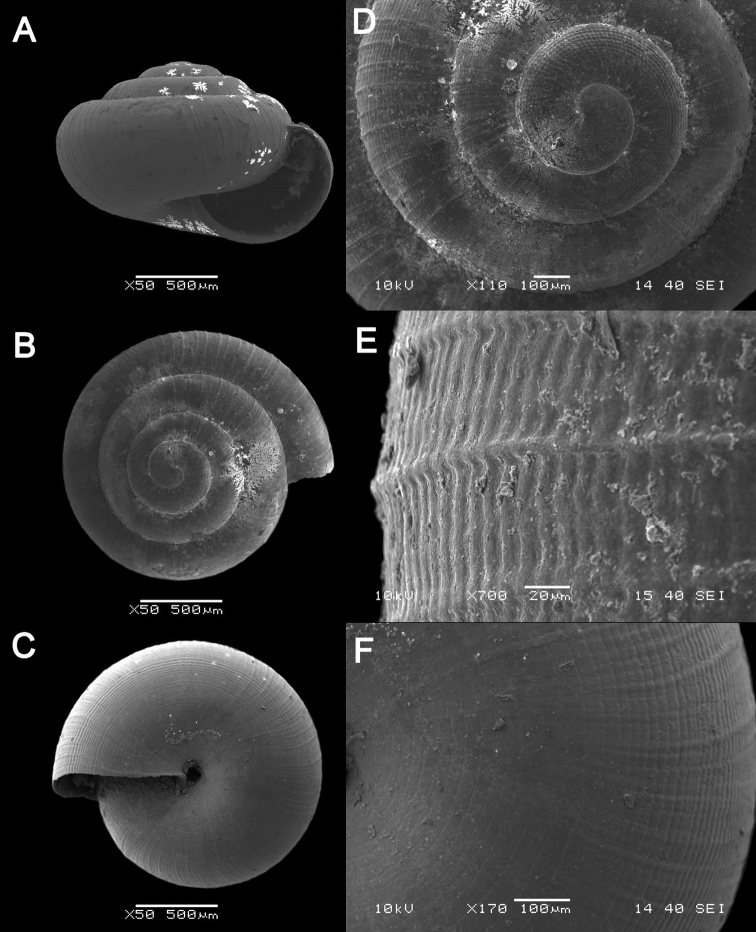
*Paralaoma angusta* Vermeulen, Liew & Schilthuizen, 2015 **A–F** ME 1763 **A** apertural view **B** apical view **C** basal view **D** enlargement of the apical side showing the apex **E** enlargement of the body whorl showing the shell sculpture **F** enlargement of the basal side of the shell.

###### Paralaoma sarawakensis sp. nov.

Taxon classificationAnimaliaStylommatophoraPunctidae

8D0AF0E9-9B06-526C-BB9C-83C884615AA0

http://zoobank.org/062C1050-2CE7-4360-8DC7-B17BB0580433

Figures 36B , 38

####### Material examined.

***Holotype*** (SH 1.27 mm, SW 2.00 mm) (MZU.MOL.20.21), Malaysia, Sarawak, Kuching Division, Bukit Sokwang (Site 3), northern site of Gunung Doya, limestone hill along Skio road, 2.05 miles E Bau, 1°23'49.87"N, 110°10'32.14"E, coll. M. E. Marzuki, 22.IV.2017. ***Paratypes***: 1 ex. (ME0008944), same data as holotype; 7 ex. (ME0008010), Gunung Sebayat, limestone hill near Bengoh resettlement scheme, along Jambusan-Semadang road, 10 miles SE Bau, 1°18'24.54"N, 110°15'21.80"E, coll. M. E. Marzuki, 13.IX.2016; >10 ex. (ME0002234), Bukit Sekunyit, limestone quarry near Batu Kitang, Kuching-Bau road, 7.2 miles E Bau, 1°25'46.81"N, 110°15'47.20"E, coll. M. E. Marzuki, 10.III.2011; 4 ex. (ME0002269), Gunung Doya, limestone hill near Sungai Sebuyoh, 3.4 miles SE Bau, 1°22'57.24"N, 110°11'39.42"E, coll. M. E. Marzuki, 10.VII.2011; 8 ex. (ME0002230), Gunung Batu, limestone outcrop along Skio road, Jambusan, 2.4 miles E Bau, 1°23'50.65"N, 110°11'19.99"E, coll. M. E. Marzuki, 23.VI.2010; 5 ex. (ME0002235), the same locality, coll. M. E. Marzuki, 11.III.2011; 9 ex. (ME0002257), the same locality, coll. M. E. Marzuki, 10.VII.2011; >10 ex. (ME0002229), Fairy Caves, south part of Gunung Kapor, 4 miles SW Bau, Kuching Division, 1°22'53.97"N, 110°7'2.29"E, coll. M. E. Marzuki, 11.III.2011; 2 ex. (ME0009211), Fairy Caves (Site 2), south part of Gunung Kapor, 4 miles SW Bau, 1°22'56.09"N, 110°6'58.82"E, coll. M. E. Marzuki, 8.IV.2017; 1 ex. (ME0009261), Buddha Caves (Site 3), north part of Gunung Kapor, 3 miles SW Bau, 1°23'26.51"N, 110°7'10.02"E, coll. M. E. Marzuki, 9.IV.2017; 1 ex. (ME0002233), South Flank of Bukit Akud, near Kampung Beratok, Serian-Kuching road, 14 miles NW Serian, 1°18'23.26"N, 110°24'15.07"E, coll. M. E. Marzuki, 21.VI.2010; 2 ex. (ME0006975), Gua Raya, along Kampung Skuduk-Chupak, 8.8 miles SE Siburan, 1°14'23.29"N, 110°25'49.05"E, coll. M. E. Marzuki, 1.I.2016; 2 ex. (MZU.MOL.20.22), >10 ex. (ME0007998), North side of Gua Raya, along Kampung Skuduk-Chupak, 8.3 miles SE Siburan, 1°14'35.10"N, 110°25'51.08"E, coll. M. E. Marzuki, 17.IX.2016; >10 ex. (ME0000430), Limestone escarpment near Kampung Benuk, 8.2 miles SW Kota Padawan, 1°18'41.43"N, 110°17'32.03"E, coll. M. E. Marzuki, 27.X.2008; >10 ex. (ME0002258), the same locality, coll. M. E. Marzuki, 22.VI.2010; >10 ex. (ME0002256), the same locality, coll. M. E. Marzuki, 9.III.2011; 7 ex. (ME0007966), the same locality, coll. M. E. Marzuki, 13.IX.2016; 3 ex. (ME0009461), the same locality, coll. M. E. Marzuki, 22.IX.2017; 1 ex. (ME0002231), Serian Division, Gua Sireh, Bukit Nambi, limestone outcrops near Kampung Taee, 7 miles W Serian, 1°10'36.18"N, 110°27'53.81"E, coll. M. E. Marzuki, 21.VI.2010; 6 ex. (ME0002232), Gunung Suka, Limestone outcrop 7.5 km from Kampung Picsing, Tebakang-Tebedu road, 8.45 miles SW Serian, 1°8'5.08"N, 110°26'53.30"E, coll. M. E. Marzuki, 20.VI.2010; >10 ex. (ME0009386), Gunung Silabur, limestone hill near Kampung Lobang Batu, 15 miles S Serian, 0°57'22.63"N, 110°30'9.36"E, coll. M. E. Marzuki, 22.IX.2017; 2 ex. (ME0000644), Miri Division, limestone outcrop near logging road, Baram Valley, 3.5 miles SW Long Bemang, 11 miles NE Long Lama, 3°49'50.36"N, 114°33'15.09"E, coll. M. E. Marzuki, 4.XI.2012; 1 ex. (ME0002751), Small limestone outcrop near Bemang-Bedian Junction, 5.4 miles E Long Lama, 3°46'15.701"N, 114°28'52.693"E, coll. M. E. Marzuki, 16.VIII.2013.

**Figure 38.**
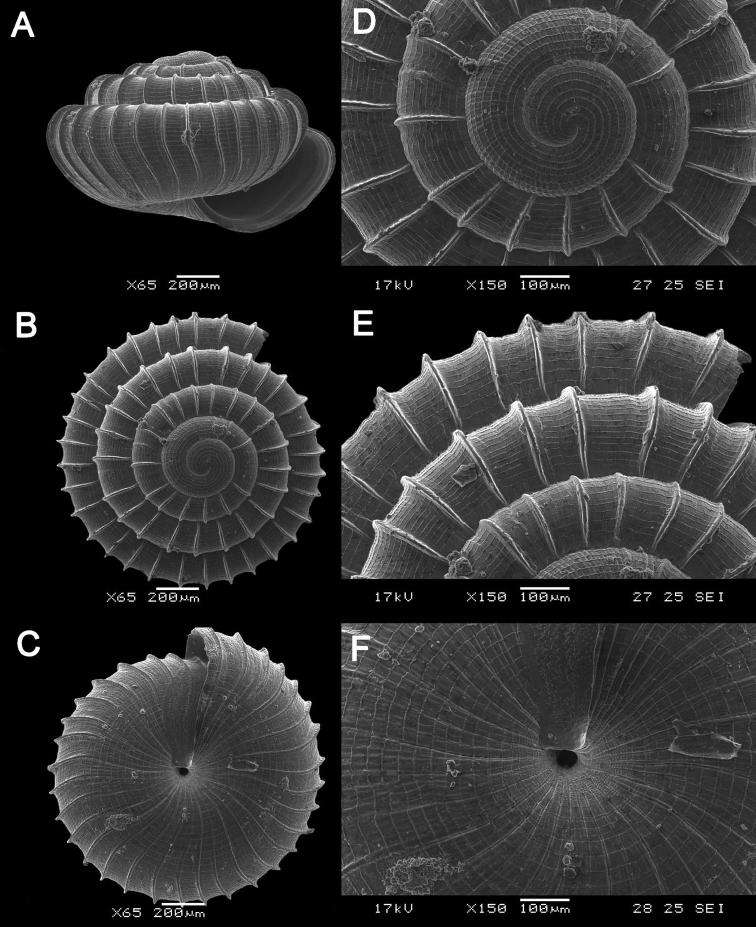
*Paralaoma sarawakensis*, sp. nov. **A–F**MZU.MOL.20.21 Holotype **A** apertural view **B** apical view **C** basal view **D** enlargement of the apical side showing the apex **E** enlargement of the teleoconch showing the shell sculpture **F** enlargement of the basal side of the shell.

####### Differential diagnosis.

It differs from ‘*Charopa’ lafargei* Vermeulen & Marzuki, 2014 of West Malaysia, by having a depressed-conical shell and a protoconch with fine, moderately spaced, spiral striations consisting of rows of minute striae which are arranged in a dashed-line pattern towards the teleoconch.

####### Description.

Shell very small, dextral, rather solid, translucent, white; spire depressed. Surface with a silky lustre. Whorls slightly convex. Number of whorls < 4¼. Protoconch with a fine, moderately spaced, spiral striation consisting of rows of minute, striae are crossed by well-spaced radial grooves arranged in a dashed-line pattern towards the teleoconch. Teleoconch: spiral sculpture present with very distinct, moderately spaced, continuous striae arranged as dashed lines. Radial sculpture of teleoconch consisting of well-spaced, coarse, orthocline, slightly sinuous, high narrow ribs which reach down to the spiral ridge and are fused to it; interstices with inconspicuous radial grooves. Periphery rounded, slightly angular; suture deep. Aperture lunulate. Peristome simple; somewhat reflected on columellar side, not thickened nor reflected on basal or palatal sides. Umbilicus open, narrow; umbilical region moderately concave. Dimensions: Shell height < 1.27 mm; shell width < 2.00 mm; diameters of the first three whorls 0.17 mm, 0.20 mm, and 0.23 mm, respectively; aperture height < 0.67 mm; aperture width < 1.00 mm.

####### Geographic distribution and habitat.

It has a wide distribution in Sarawak. Living snails were observed foraging among leaf litter and plant debris near the cliffs in a lowland limestone forest and in a lowland non-limestone forest.

####### Etymology.

The specific epithet *sarawakensis* is derived from the name of Malaysian State of Sarawak.

#### Family Charopidae Hutton, 1884

##### *Charopa* Albers, 1860

###### Charopa sp. ‘argos’

Taxon classificationAnimaliaStylommatophoraCharopidae

5DF5D904-0B1B-5EB8-9303-9F765FF0E3D5

Figure 36C , 39

####### Type locality.

Not applicable.

####### Material examined.

Gunung Doya: ME 8593, ME 8998. Kampung Padang Pan: ME 9491. Gunung Kapor: ME 9896, ME 9136. Gunung Batu: ME 7177, ME 1890. Lobang Angin: ME 9039, ME 9138.

####### Distribution in Borneo.

Sarawak: Kuching and Miri divisions. Sabah: Exact location was not mentioned in Clements et al. (2008). Endemic to Borneo.

####### Remarks.

This species was recorded in Clements et al. (2008) as *Charopa argos*. This species will be described in a separate publication on Sabah land snails. Living snails were observed foraging among leaf litter and plant debris near the cliff in lowland limestone forest. It differs from other Bornean *Charopa* species by its prominent, rather deep, well-spaced radial grooves crossing the spiral striae at more or less regular intervals on the shell surface.

**Figure 39.**
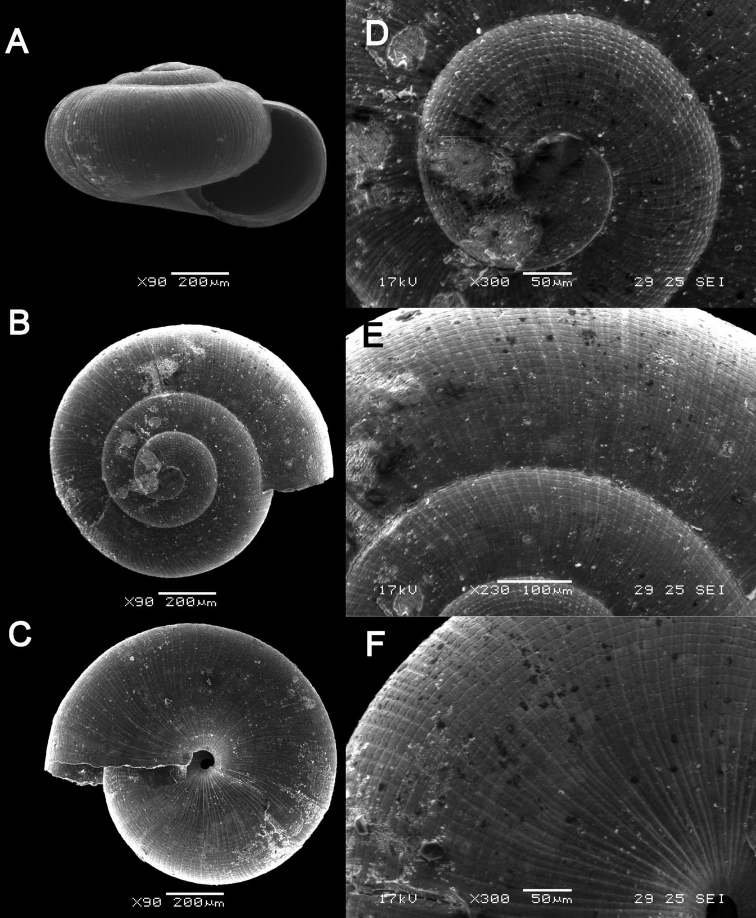
*Charopa* sp. “argos”. **A–F**MZU.MOL.20.08 **A** apertural view **B** apical view **C** basal view **D** enlargement of the apical side showing the apex **E** enlargement of the teleoconch showing the shell sculpture **F** enlargement of the basal side of the shell.

#### Family Dyakiidae Gude & B. B. Woodward, 1921

##### *Dyakia* Godwin-Austen, 1891

###### Dyakia busanensis

Taxon classificationAnimaliaStylommatophoraDyakiidae

Godwin-Austen, 1891

E3161EFF-CA7F-5736-BF54-B9D99DDC9CB6

Figure 40A

Dyakia busanensis Godwin-Austen, 1891: 31.

####### Type locality.

“Busan Hills, Borneo” [= Jambusan Hills, Bau, Sarawak].

####### Material examined.

Gunung Doya: ME 1600, ME 8913, ME 9015, ME 9159. Gunung Kapor: ME 1595, ME 1596, ME 1598, ME 1599, ME 8082, ME 8457, ME 9056, ME 9217. Gunung Batu: ME 1597, ME 4861, ME 8811.

####### Distribution in Borneo.

Sarawak: Kuching and Sri Aman divisions. Endemic to Borneo.

####### Remarks.

Another form of this species was described by Smith (1895) as *concolor* from Sri Aman, Sarawak.

**Figure 40.**
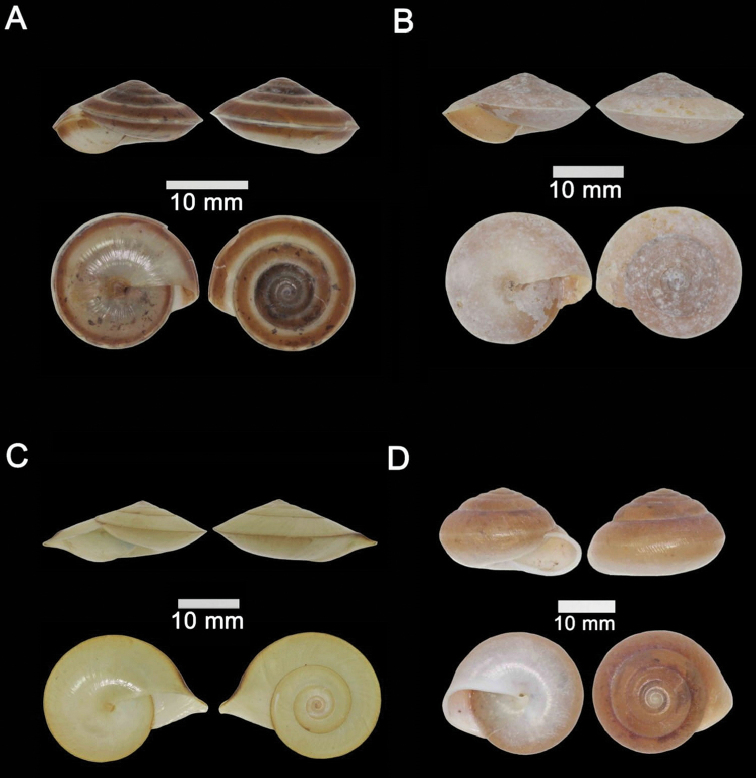
**A***Dyakia busanensis* Godwin-Austen, 1891 ME 9015 Gunung Doya **B***Dyakia subdebilis* E. A. Smith, 1895 ME 1602 Gunung Kapor **C***Rhinocochlis nasuta* (Metcalfe, 1851) ME 8740 Lobang Angin **D***Quantula striata* (Gray, 1834) ME 8792 Gunung Kapor.

###### Dyakia subdebilis

Taxon classificationAnimaliaStylommatophoraDyakiidae

E. A. Smith, 1895

1C349ECD-4ED1-585C-BEA1-AD8EC231AF0C

Figure 40B

Dyakia subdebilis E. A. Smith, 1895: 104, pl. 2, fig. 11.

####### Type locality.

“Sarawak”.

####### Material examined.

Gunung Sebayat: ME 8303. Gunung Kapor: ME 1602.

####### Distribution in Borneo.

Sarawak: Kuching Division. Endemic to Borneo.

####### Remarks.

According to MolluscaBase, this is a “Taxon inquirendum”. This species was placed as junior synonym of *Dyakia regalis* (Benson, 1850) by Laidlaw (1963). However, the shells from Bau are different from *Dyakia regalis* (Benson, 1850) in having a straw-yellow to light brown shell with a shiny surface below the periphery.

##### *Everettia* Godwin-Austen, 1891

###### Everettia cutteri

Taxon classificationAnimaliaStylommatophoraDyakiidae

(H. Adams, 1870)

E716F652-5A9D-5D0D-BFAE-33F0ACB30F07

Figure 41A

Macrochlamys cutteri H. Adams, 1870: 794, pl. 48, fig. 21.

####### Type locality.

“Busan, near Sarawak, Borneo” [= Jambusan Hills, Bau, Sarawak].

####### Material examined.

Gunung Doya: ME 8961. Gunung Kapor: ME 1630, ME 8968. Gunung Batu: ME 1628, ME 1629.

####### Distribution in Borneo.

Sarawak: Kuching and Miri divisions. Endemic to Borneo.

####### Remarks.

Only dry shells were found during the surveys. This species is different from both *Xesta baramensis* Kobelt, 1897 and *Vitrinula moluensis* (E. A. Smith, 1893) in having a shell with a wide, pale brown band encircling the periphery. Anatomical studies by Godwin-Austen (1891) confirmed the placement of this species in the genus *Everettia*.

**Figure 41.**
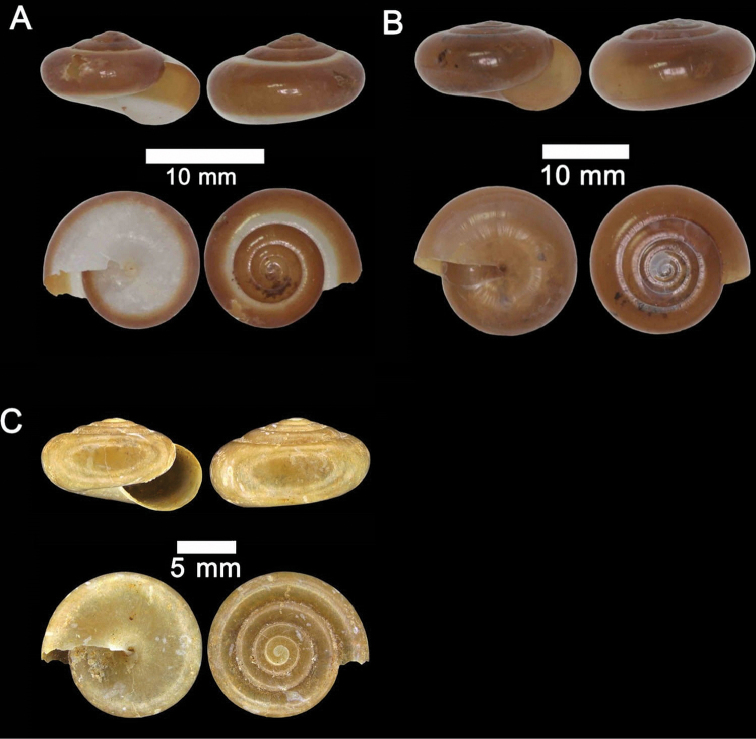
**A***Everettia cutteri* (H. Adams, 1870) ME 8968 Gunung Doya Kapor **B***Everettia microrhytida*, sp. nov., MZU.MOL.20.25 Holotype Gunung Batu **C***Everettia minuta*, sp. nov., MZU.MOL.20.23 Holotype Gunung.

###### Everettia microrhytida sp. nov.

Taxon classificationAnimaliaStylommatophoraDyakiidae

1F165D15-C026-5803-ADF5-C630CE00282E

http://zoobank.org/EDF2CDBE-F06C-423B-8442-F7AC1DC20BE7

Figures 41B , 42D–F

####### Material examined.

***Holotype*** (SH 12.14 mm, SW 22.00 mm) (MZU.MOL.20.25), Malaysia, Sarawak, Kuching Division, Gunung Batu, limestone outcrop along Skio road, Jambusan, 2.4 miles E Bau, 1°23'50.65"N, 110°11'19.99"E, coll. M. E. Marzuki, 10.II.2017. ***Paratypes***: 1 ex. (MZU.MOL.20.26), the same locality as holotype, coll. M. E. Marzuki, 10.VII.2011; 1 ex. (ME0006829), small limestone escarpment near Kampung Padang Pan, 15 miles SW Bau, 1°19'24.07"N, 110°3'46.34"E, coll. M. E. Marzuki, 27.IX.2015; 2 ex. (ME0003498), small limestone outcrop at Kampung Beratok, Serian-Kuching road, 14.3 miles NW Serian, 1°18'41.05"N, 110°24'37.13"E, coll. M. E. Marzuki, 21.VI.2010; 6 ex. (ME0009145), Lobang Angin (Site 3), limestone outcrop near Sungai Sarawak Kanan, 1.75 miles W of Bau, 1°24'54.96"N, 110°8'13.62"E, coll. M. E. Marzuki, 23.IV.2017; 1 ex. (ME0009845), the same locality, coll. M. E. Marzuki, 12.V.2018.

####### Differential diagnosis.

This species is similar to *Everettia consul* (Pfeiffer, 1854) in terms of general shape and size. However, it differs from *E. consul* by lacking spiral sculpture and having only very fine (sometimes inconspicuous), somewhat wrinkled, puncture-like sculpture on both the apical and apertural sides. *Everettia consul*, on the other hand, has a shell with a more elevated spire and its shell surface has densely placed radial threads and somewhat cut by irregularly spaced spiral grooves.

**Figure 42.**
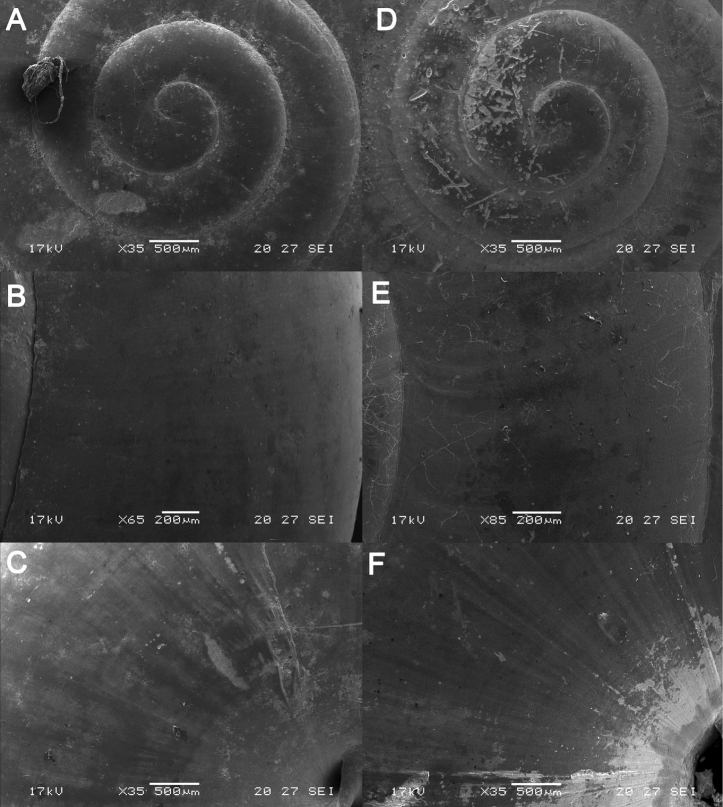
*Everettia* spp. **A–C***Everettia minuta*, sp. nov., MZU.MOL.20.24 Paratype **A** Enlargement of the apical side showing the apex **B** Enlargement of the teleoconch showing the shell sculpture **C** Enlargement of the basal side of the shell **D–F***Everettia microrhytida*, sp. nov., MZU.MOL.20.26 Paratype **D** Enlargement of the apical side showing the apex **E** Enlargement of the teleoconch showing the shell sculpture **F** Enlargement of the basal side of the shell.

####### Description.

Shell moderately large, rather thin, translucent, pale to dark brown, spire slightly elevated. Surface with a glossy lustre. Whorls convex. Number of whorls < 6¼. Protoconch: almost smooth, spiral striation absent with inconspicuous radial threads near the suture. Teleoconch with no: spiral sculpture but with very fine, inconspicuous, somewhat wrinkled, puncture-like shell sculptures on both apical and apertural sides. Radial sculpture of teleoconch very fine as well as inconspicuous growth lines, most conspicuous radial threads near the suture and below periphery. Periphery round; suture shallow. Aperture lunulate. Peristome simple; somewhat thickened and reflected on columellar side, not thickened nor reflected on basal and palatal sides. Umbilicus open, narrow; sometimes partly covered by reflected peristome; umbilical region moderately concave. Dimensions: shell height < 12.58 mm; shell width < 22.13 mm; diameters of the first three whorls 1.60 mm, 2.55 mm, and 4.90 mm, respectively; aperture height < 8.94 mm; aperture width < 11.66 mm.

####### Geographic distribution and habitat.

It is known from the Bau and Serian-Padawan limestone hill clusters. Living snails were observed foraging among leaf litter and plant debris near the cliff in a lowland limestone forest.

####### Etymology.

From the Greek *mikro rytídes*, meaning extremely small wrinkles, in reference to the shell sculpture.

###### Everettia minuta sp. nov.

Taxon classificationAnimaliaStylommatophoraDyakiidae

1044D56A-3243-59E7-B65F-95D51553C043

http://zoobank.org/D8EF63CB-DA58-4A13-B619-10D768EF6117

Figures 41C , 42A–C

####### Material examined.

Holotype (SH 5.72 mm, SW 10.00 mm) (MZU.MOL.20.23), Malaysia, Sarawak, Kuching Division, Fairy Caves (Site 1), south part of Gunung Kapor, 4 miles SW Bau, 1°22'53.76"N, 110°7'4.34"E, coll. M. E. Marzuki, 8.IV.2017. Paratypes: 7 ex. (ME0008965), same data as the holotype; 1 ex. (MZU.MOL.20.24), the same locality as the holotype, coll. M. E. Marzuki, 23.VI.2010; 5 ex. (ME0001512), the same locality, coll. M. E. Marzuki, 11.III.2011; 1 ex. (ME0001513), Gunung Batu, limestone outcrop along Skio road, Jambusan, 2.4 miles E Bau, 1°23'50.65"N, 110°11'19.99"E, coll. M. E. Marzuki, 11.III.2011; 4 ex. (ME0009139), Lobang Angin (Site 1), limestone outcrop near Sungai Sarawak Kanan, 1.75 miles W of Bau, 1°24'48.14"N, 110°8'12.21"E, coll. M. E. Marzuki, 15.IV.2017; 1 ex. (ME0009222), Lobang Angin (Site 2), limestone outcrop near Sungai Sarawak Kanan, 1.75 miles W of Bau, 1°24'51.01"N, 110°8'13.48"E, coll. M. E. Marzuki, 16.IV.2017; 1 ex. (ME0009466), Serian Division; Gunung Storib, small northern peak of Gunung Silabor, 15 miles S Serian, 0°57'30.75"N, 110°30'3.00"E, 22.IX.2017.

####### Differential diagnosis.

The new species is similar to *Everettia jucunda* (Pfeiffer, 1863), *E. bangueyensis* (Smith, 1895) and *E. jucundior* Liew, Vermeulen & Schilthuizen, 2009 from Sabah. *Everettia jucunda* differs by having a larger shell (< 17.3 mm wide), with one and half more whorls. *Everettia jucundior* differs by having a larger shell (< 19.5 mm wide) with slightly shouldered whorls. *Everettia bangueyensis* differs by having a smaller shell (< 9.0 mm wide), and a flat spire with slightly shouldered whorls.

####### Description.

Shell small, rather thin, translucent, pale brown, spire moderately elevated. Surface with a glossy lustre. Whorls convex. Number of whorls < 5¼. Protoconch almost smooth, spiral striation absent with inconspicuous radial threads near the suture. Teleoconch: spiral sculpture with inconspicuous, densely placed spiral grooves on both apical side and apertural sides. Radial sculpture almost smooth, with inconspicuous radial threads near suture and below periphery. Periphery round; suture slightly depressed. Aperture lunulate. Peristome simple; continuous, somewhat sinuous, thickened, and reflected on columellar side, not thickened nor reflected on basal and palatal sides. Umbilicus narrow, partly covered by the reflected peristome; umbilical region moderately concave. Dimensions: shell height < 7.62 mm; shell width < 13.17 mm; diameters of the first three whorls 1.45 mm, 2.40 mm, and 4.34 mm, respectively; aperture height < 4.65 mm; aperture width < 6.58 mm.

####### Geographic distribution and habitat.

Known from the Bau and Serian-Padawan limestone hills. Only dry shells were not found during the surveys.

####### Etymology.

From the Latin *minuta*, meaning small, in reference to the smaller shell compare to other species of *Everettia* from Sarawak.

#### *Rhinocochlis* Thiele, 1931

##### Rhinocochlis nasuta

Taxon classificationAnimaliaStylommatophoraDyakiidae

(Metcalfe, 1851)

D9A691F1-828D-5D26-80AA-16B4AEE8BCAF

Figures 40C , 53C

Helix nasuta Metcalfe, 1851: 70.

###### Type locality.

“Borneo”.

###### Material examined.

Bukit Sekunyit: ME 4878. Gunung Doya: ME 1610, ME 8914, ME 8924, ME 9040. Gunung Kapor: ME 4868, ME 4872, ME 4874, ME 8081, ME 8456, ME 8772, ME 8971, ME 9406. Gunung Stulang: ME 5906. Lobang Angin: ME 4882, ME 4883, ME 8727, ME 8740, ME 8885. Gunung Batu: ME 4869, ME 4870, ME 4871.

###### Distribution in Borneo.

Sarawak: Kuching, Serian, Kapit, and Miri divisions. Kalimantan: West and East Kalimantan provinces. Endemic to Borneo.

###### Remarks.

It differs from *Rhinocochlis moluensis* (Godwin-Austen, 1891), and *Dyakia chlorosoma* Vermeulen, Liew & Schilthuizen, 2015, by having a shell with the curved beak-like extension of the palatal side of the aperture.

####### *Quantula* H. B. Baker, 1941

##### Quantula striata

Taxon classificationAnimaliaStylommatophoraDyakiidae

(Gray, 1834)

4FCECF36-17B8-5085-84B6-EC8A79B4383D

Figure 40D

Nanina striata Gray, 1834: 59.

###### Type locality.

Not stated.

###### Material examined.

Gunung Kapor: ME 8792. Kampung Padang Pan: ME 6676.

###### Distribution in Borneo.

Sarawak: Kuching, Samarahan, Mukah and Miri divisions. Labuan: Labuan and Papan Islands. ***Distribution elsewhere.*** West Malaysia, Singapore, and China (Benson 1842; Foon et al. 2017).

###### Remarks.

Probably an introduced species. The species is only known from disturbed habitats in Borneo.

#### Family Trochomorphidae Möllendorff, 1890

##### *Geotrochus* van Hasselt, 1823

###### Geotrochus conicoides

Taxon classificationAnimaliaStylommatophoraTrochomorphidae

(Metcalfe, 1851)

5FEF7426-87CD-5AB7-BA22-223DC67AE2F5

Figure 43A

Helix conicoides Metcalfe, 1851: 71.

####### Type locality.

“Borneo”.

####### Material examined.

Gunung Doya: ME 1637, ME 8911.

####### Distribution in Borneo.

Sarawak: Kuching, Serian and Miri divisions. Sabah: West Coast and Tawau divisions. Kalimantan: West Kalimantan Province. ***Distribution elsewhere.*** Sumatra (?) (Van Benthem-Jutting, 1959).

####### Remarks.

Only dry shells were found during the surveys. The shells from Bau have a more depressed shell than the shells from Niah.

**Figure 43.**
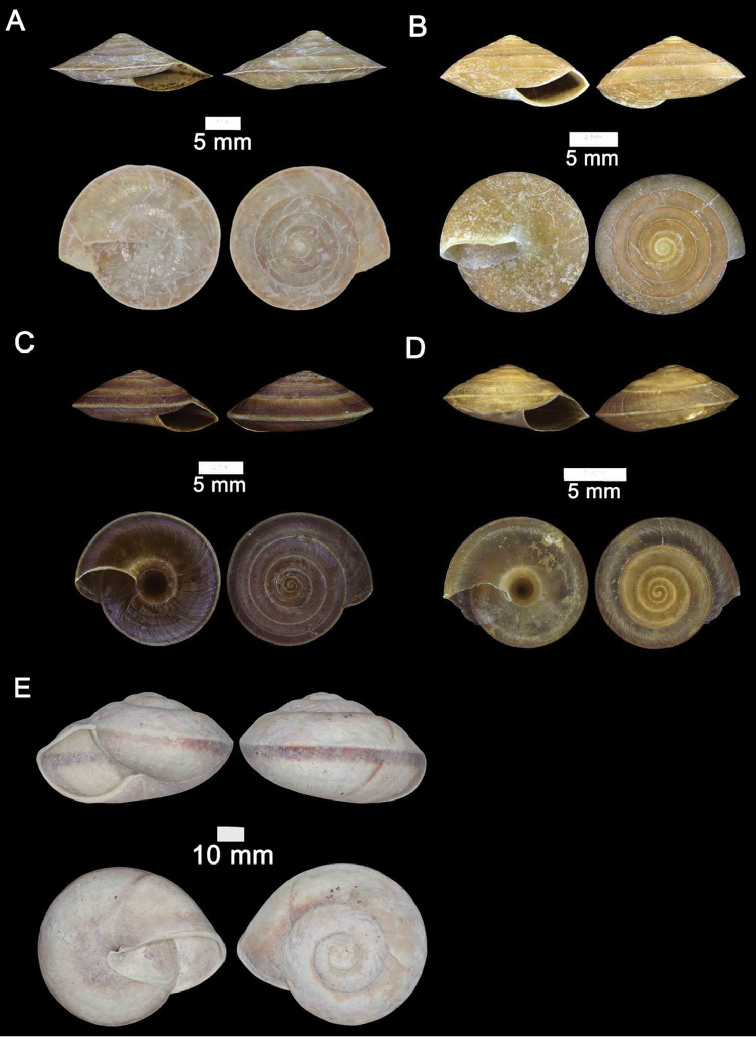
**A***Geotrochus conicoides* (Metcalfe, 1851) ME 8911 Gunung Doya **B***Geotrochus subscalaris* Vermeulen, Liew & Schilthuizen, 2015 ME 1636 Gunung Kapor **C***Videna bicolor* (Martens, 1864) ME 8732 Lobang Angin **D***Videna timorensis* (Martens, 1867) ME 8972 Gunung Kapor **E***Exrhysota brookei* (A. Adams & Reeve, 1848) ME 8790 Gunung Kapor.

###### Geotrochus subscalaris

Taxon classificationAnimaliaStylommatophoraTrochomorphidae

Vermeulen, Liew & Schilthuizen, 2015

B2472D24-1B34-5554-9A97-BE9012448B94

Figure 43B

Geotrochus subscalaris Vermeulen et al., 2012: 129–131, fig. 91.

####### Type locality.

“Malaysia, Sabah, Sandakan Province, Kinabatangan valley, Batu Pangi”.

####### Material examined.

*Gunung Kapor*: ME 1636.

####### Distribution in Borneo.

Sarawak: Kuching Division. Sabah: West Coast Division. Endemic to Borneo.

####### Remarks.

This is the first record of this species in Sarawak. No living snail was found during the surveys. The shells from Bau are more depressed than the shells from Sabah.

##### *Videna* H. Adams & A. Adams, 1855

###### Videna bicolor

Taxon classificationAnimaliaStylommatophoraTrochomorphidae

(Martens, 1864)

837CCFE9-258A-5A88-AB7B-9245A4F35958

Figures 43C , 52B

Trochomorpha bicolor Martens, 1864: 267.

####### Type locality.

“Im mittleren Sumatra” [= Central Sumatra].

####### Material examined.

Bukit Sekunyit: ME 1202. Gunung Doya: ME 3356, ME 8910, ME 9032, ME 9116. Gunung Kapor: ME 1199, ME 1205, ME 3361, ME 8775, ME 9225, ME 9844. Kampung Bunga Rampai: ME 1198. Lobang Angin: ME 8732, ME 9271. Gunung Batu: ME 1106, ME 1203, ME 1209, ME 8825.

####### Distribution in Borneo.

Sarawak: Kuching, Serian, Sibu, Mukah, Kapit, and Miri divisions. Sabah: Interior and West Coast divisions. Kalimantan: West and South Kalimantan provinces. ***Distribution elsewhere.*** Sumatra to Lesser Sunda (Vermeulen and Whitten 1998).

####### Remarks.

Living snails were observed foraging on wet rotten wood surfaces and crown of a plant of the limestone cliff. It differs from *V. timorensis* (Martens, 1867) by having a medium-sized dark brown shell with wide umbilicus and a smooth shell surface. This is the most common *Videna* species in Sarawak.

###### Videna timorensis

Taxon classificationAnimaliaStylommatophoraTrochomorphidae

(Martens, 1867)

F5FE8D42-7881-5DBE-866E-E963249BE32E

Figures 43D , 52A

Trochomorpha timorensis Martens, 1867: 248–249, pl. 13, fig. 6.

####### Type locality.

“Timor, im Innern bei Okabiti, in Waldern” [= near Okabiti, Timor Island].

####### Material examined.

Gunung Doya: ME 1228, ME 9163, ME 9193, ME 9405. Gunung Kapor: ME 1179, ME 1222, ME 1226, ME 3360, ME 5976, ME 8090, ME 8458, ME 8762, ME 8972. Kampung Padang Pan: ME 6724. Lobang Angin: ME 8984, ME 9404. Gunung Batu: ME 1224, ME 8824.

####### Distribution in Borneo.

Sarawak: Kuching Division. Sabah: Tawau Division. ***Distribution elsewhere.*** Peninsular Malaysia to Indo-Australian archipelago (Maassen 2001).

####### Remarks.

Living snails were observed foraging on wet rotten wood surfaces at the base of the limestone cliff. It differs from *V. bicolor* (Martens, 1864) by having a pale brown smaller shell with narrower umbilicus and shell surface striated with conspicuous spiral grooves.

#### Family Ryssotidae Schileyko, 2003

##### *Exrhysota* H. B. Baker, 1941

###### Exrhysota brookei

Taxon classificationAnimaliaStylommatophoraRyssotidae

(A. Adams & Reeve, 1848)

C6F7C745-2AF1-5CEB-84FB-3D4A77D48092

Figure 43E

Helix brookei A. Adams & Reeve, 1850: 60, pl. 15, fig. 4A, B.

####### Type locality.

“Mountains of Borneo”.

####### Material examined.

Gunung Kapor: ME 8083, ME 8790.

####### Distribution in Borneo.

Sarawak: Kuching, Sibu, Kapit and Miri divisions. Sabah: Sandakan, Tawau and East Coast divisions. Kalimantan: West, South, and East Kalimantan provinces. Endemic to Borneo.

####### Remarks.

This is the largest native land snail species in Borneo. For further details on the generic and familial placement of this species, see Sutcharit et al. (2019: 2).

#### Family Helicarionidae Bourguignat, 1877

##### *Microcystis* Beck, 1838

###### Microcystis dyakana

Taxon classificationAnimaliaStylommatophoraHelicarionidae

Godwin-Austen, 1891

2A3BB79A-C4A2-57DD-8089-A87A1937C00F

Figures 44A , 52D

Microcystis dyakana Godwin-Austen, 1891: 36–37, pl. 4, figs 4, 4C.

####### Type locality.

“Busan Hills, Borneo” [= Jambusan Hills, Bau, Sarawak].

####### Material examined.

Bukit Sekunyit: ME 2850. Gunung Doya: ME 8955, ME 8999, ME 9156. Gunung Kapor: ME 2879, ME 2881, ME 2947, ME 5978, ME 8087, ME 8459, ME 8770, ME 8969. Kampung Padang Pan: ME 6679. Lobang Angin: ME 8751, ME 9398. Gunung Batu: ME 2840, ME 2867, ME 8815.

####### Distribution in Borneo.

Sarawak: Kuching, Serian and Miri divisions. ***Distribution elsewhere.*** Lombok (Smith 1899).

####### Remarks.

Living snails were observed foraging on the leaf surfaces of small trees and palms at the base of limestone cliffs.

**Figure 44.**
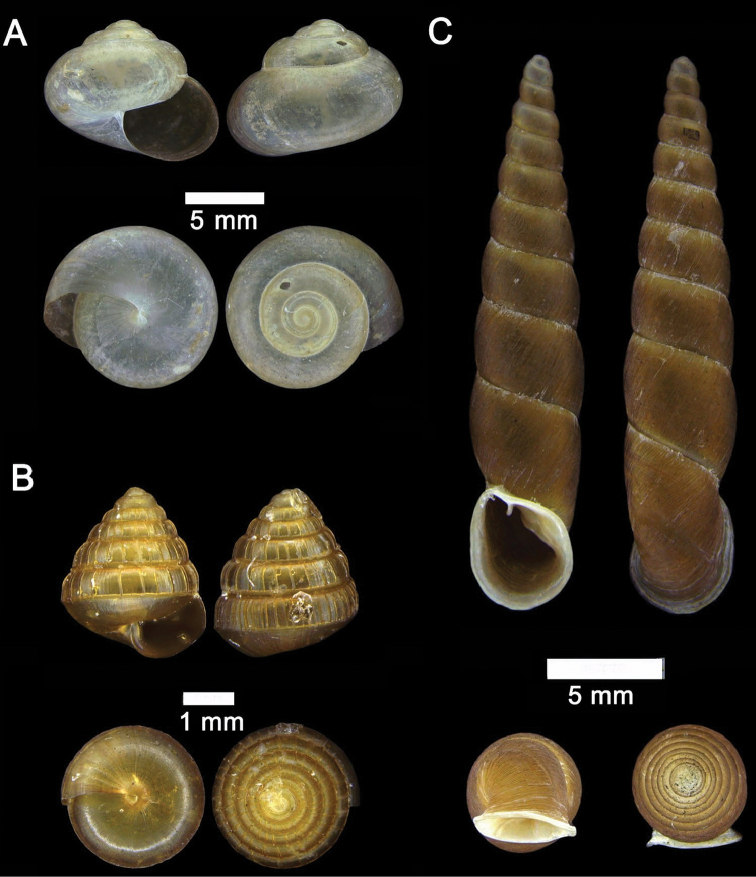
**A***Microcystis dyakana* Godwin-Austen, 1891 ME 8770 Gunung Kapor **B***Rahula raricostulata* (E. A. Smith, 1893) ME 8938 Lobang Angin **C***Phaedusa borneensis* (L. Pfeiffer, 1854) ME 8784 Gunung Kapor.

#### Family Euconulidae H. B. Baker, 1928

##### *Rahula* Godwin-Austen, 1907

###### Rahula raricostulata

Taxon classificationAnimaliaStylommatophoraEuconulidae

(E. A. Smith, 1893)

A009C727-D37D-5636-9F8F-BF832A348C52

Figure 44B

Sitala raricostulata E. A. Smith, 1893: 342–343, pl. 25, fig. 2.

####### Type locality.

“Busau or Busan, Sarawak” [= Jambusan Hills, Bau, Sarawak].

####### Material examined.

Gunung Doya: ME 8937, ME 8962. Gunung Kapor: ME 0860, ME 1508, ME 9097, ME 9207. Lobang Angin: ME 8938, ME 9221. Gunung Batu: ME 1891.

####### Distribution in Borneo.

Sarawak: Kuching and Serian divisions. Endemic to Borneo.

####### Remarks.

Only dry shells were found during the surveys. It differs from *Rahula delopleura* Vermeulen, Liew & Schilthuizen, 2015, by having a shell with distinct, prominent spiral lirae on the protoconch.

#### Family Clausiliidae J. E. Gray, 1855

##### *Phaedusa* H. Adams & A. Adams, 1855

###### Phaedusa borneensis

Taxon classificationAnimaliaStylommatophoraClausiliidae

(L. Pfeiffer, 1854)

5B90E8EB-78A8-5C75-8A30-2E9B4B503D28

Figures 44C , 50C

Clausilia borneensis L. Pfeiffer, 1854a: 296.

####### Type locality.

“Sarawak, Borneo”.

####### Material examined.

Gunung Doya: ME 2904, ME 8912, ME 9153. Gunung Kapor: ME 2890, ME 2891, ME 2892, ME 2893, ME 2944, ME 5975, ME 8784, ME 9257. Gunung Batu: ME 2897, ME 2898.

####### Distribution in Borneo.

Sarawak: Kuching, Serian, and Miri divisions. Endemic to Borneo.

####### Remarks.

Living snails were observed foraging on the moderately wet vertical limestone rock surfaces covered with lichens.

#### Family Valloniidae Morse, 1864

##### *Ptychopatula* Pilsbry, 1889

###### Ptychopatula dioscoricola

Taxon classificationAnimaliaStylommatophoraValloniidae

(C. B. Adams, 1845)

3F246253-2EA2-5BE3-B413-8BC2CF8DA17D

Figure 45A

Helix dioscoricola C. B. Adams, 1845: 16.

####### Type locality.

“Jamaica”.

####### Material examined.

Gunung Doya: ME 8942, ME 8997. Gunung Kapor: ME 8151, ME 8502, ME 9006, ME 9027, ME 9070. Kampung Padang Pan: ME 6830. Lobang Angin: ME 9140, ME 9173.

####### Distribution in Borneo.

Sarawak: Kuching, Bintulu, Miri, and Limbang divisions. Sabah: Sandakan Division. ***Distribution elsewhere.*** Circumtropical (Pilsbry 1920–1921).

####### Remarks.

Only dry shells were found during the surveys. It differs from *P. circumlitum* (Hedley, 1897) and *P. orcella* (Stoliczka, 1873) in having a higher spired shell with an umbilicus that is partly or entirely covered by the reflected peristome. This species also differs from *P. pulvisculum* (Issel, 1874) in having a larger shell with spiral lirae on shell surfaces.

**Figure 45.**
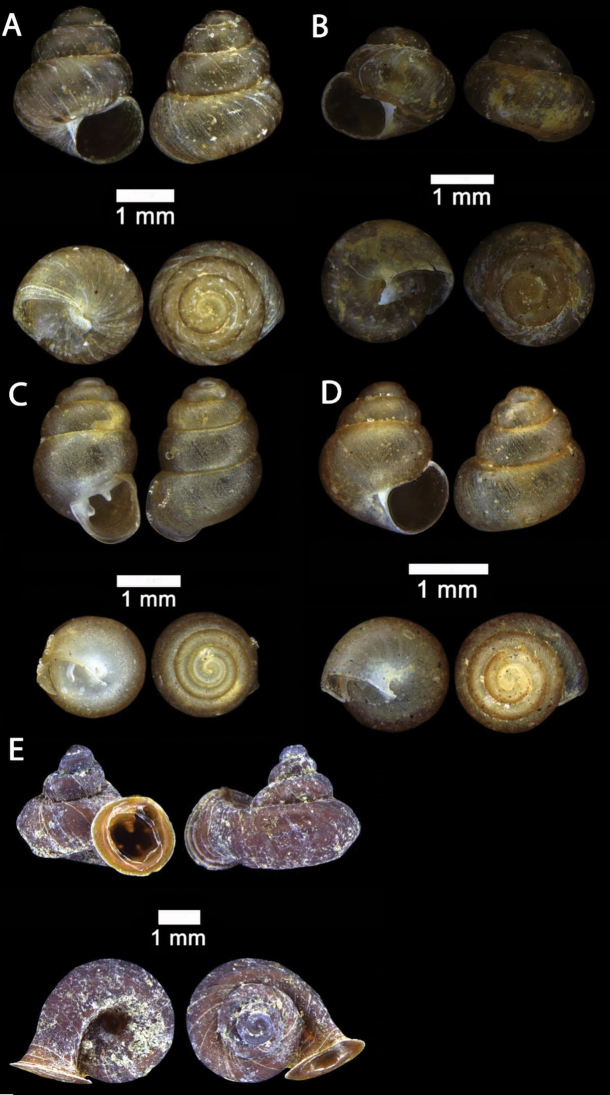
**A***Ptychopatula dioscoricola* (C. B. Adams, 1845) ME 9070 Gunung Kapor **B***Ptychopatula orcella* (Stoliczka, 1873) ME 8925 Gunung Doya **C***Pupisoma moleculina* (Van Benthem-Jutting, 1940) ME 9051 Gunung Kapor **D***Pupisoma pulvisculum* (Issel, 1874) ME 9055 Gunung Kapor **E***Boysidia salpinx* F. G. Thompson & Dance, 1983 ME 8781 Gunung Kapor.

**Figure 46.**
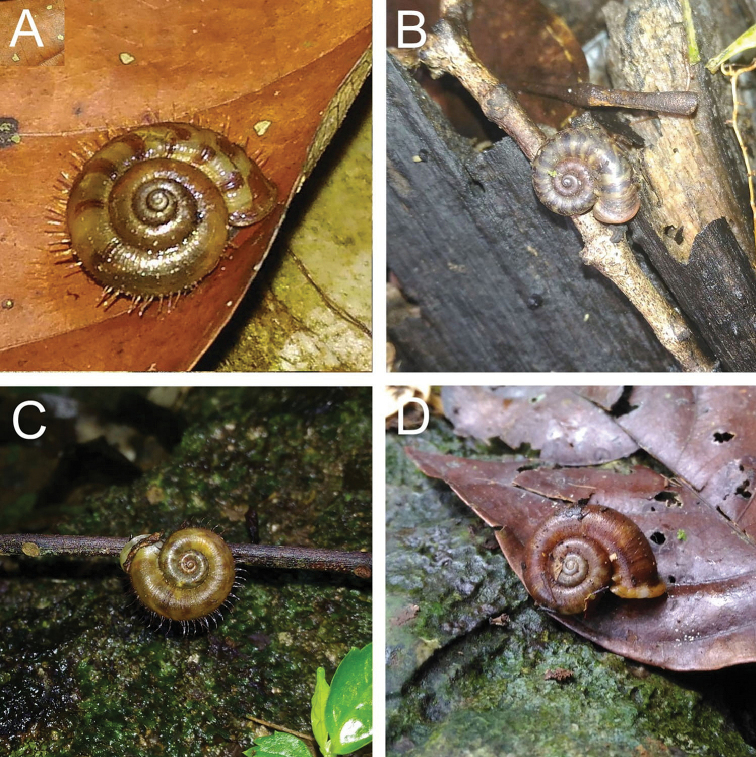
Living snails from Bau **A***Japonia barbata* (L. Pfeiffer, 1855) ME 8768 Gunung Kapor **B***Opisthophorus euryomphalus* (L. Pfeiffer, 1856) ME 8779 Gunung Kapor **C***Opisthophorus biciliatus* Mousson, 1849 ME 8754 Gunung Kapor **D***Opisthophorus birostris* (L. Pfeiffer, 1854) ME 8755 Gunung Kapor. All not to scale.

###### Ptychopatula orcella

Taxon classificationAnimaliaStylommatophoraValloniidae

(Stoliczka, 1873)

26CE32C9-0C25-5F2A-BD9B-32279E082CDA

Figure 45B

Pupa (Pupisoma) orcella Stoliczka, 1873: 33, pl. 2, fig. 2.

####### Type locality.

“Penang island”.

####### Material examined.

Gunung Doya: ME 8925.

####### Distribution in Borneo.

Sarawak: Kuching Division. Sabah: Tawau Division. ***Distribution elsewhere.*** Malay Peninsula, Indo-Australian archipelago (Vermeulen and Whitten 1998).

####### Remarks.

Only dry shells were found during the surveys. It differs from *Ptychopatula circumlitum* (Hedley, 1897) and *P. dioscoricola* (C. B. Adams, 1845) by having irregularly spaced ribs on shell surfaces (visible at 40 × magnification). This is probably the first record of sinistral form for species in the Genus *Ptychopatula*. For further details, see Pilsbry (1920–1921: 29).

**Figure 47.**
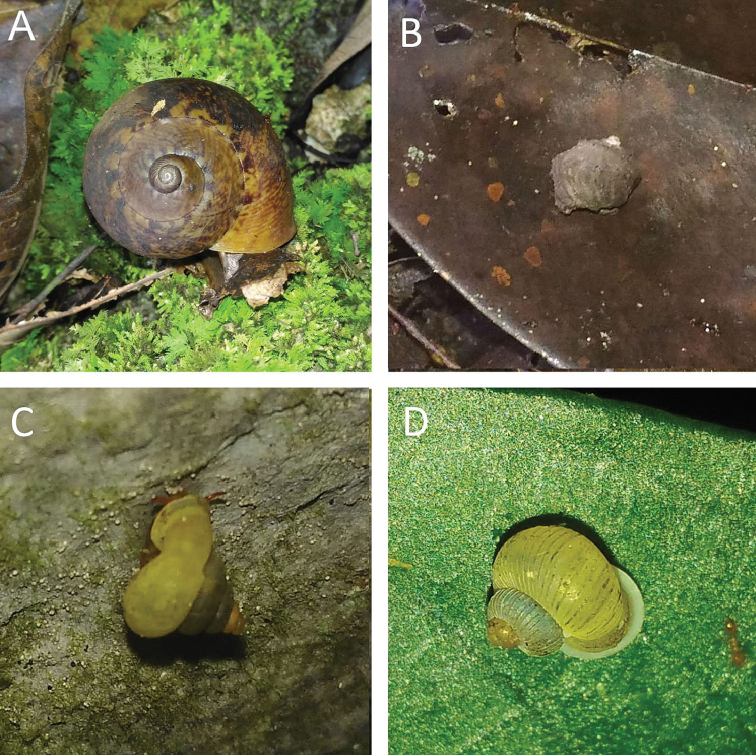
Living snails from Bau **A***Cyclophorus perdix borneensis* (Metcalfe, 1851) ME 8753 Gunung Kapor **B***Platyrhaphe linita* (Godwin-Austen, 1889) ME 9700 Gunung Doya **C***Stomacosmethis sadongensis* (E. A. Smith, 1895) ME 8761 Gunung Kapor **D***Pincerna globosa* (H. Adams, 1870) ME 8749 Lobang Angin. All not to scale.

##### *Pupisoma* Stoliczka, 1873

###### Pupisoma moleculina

Taxon classificationAnimaliaStylommatophoraValloniidae

(Van Benthem-Jutting, 1940)

86E74882-F66F-502F-83F6-AD175ED30032

Figure 45C

Costigo moleculina Van Benthem-Jutting, 1940: 331–332.

####### Type locality.

“Forest between the village of Tjisolok and the hot springs (Tjipanas) some miles inland, south coast of West Java”.

####### Material examined.

Gunung Kapor: ME 9051, ME 9212.

####### Distribution in Borneo.

Sarawak: Kuching Division. Sabah: Sandakan Division. ***Distribution elsewhere.*** Peninsular Malaysia, Sumatra, and Java (Van Benthem-Jutting 1940; Vermeulen and Raven 1998; Maassen 2000).

####### Remarks.

This is the first record of this species in Sarawak. Only dry shells were found during the surveys.

**Figure 48.**
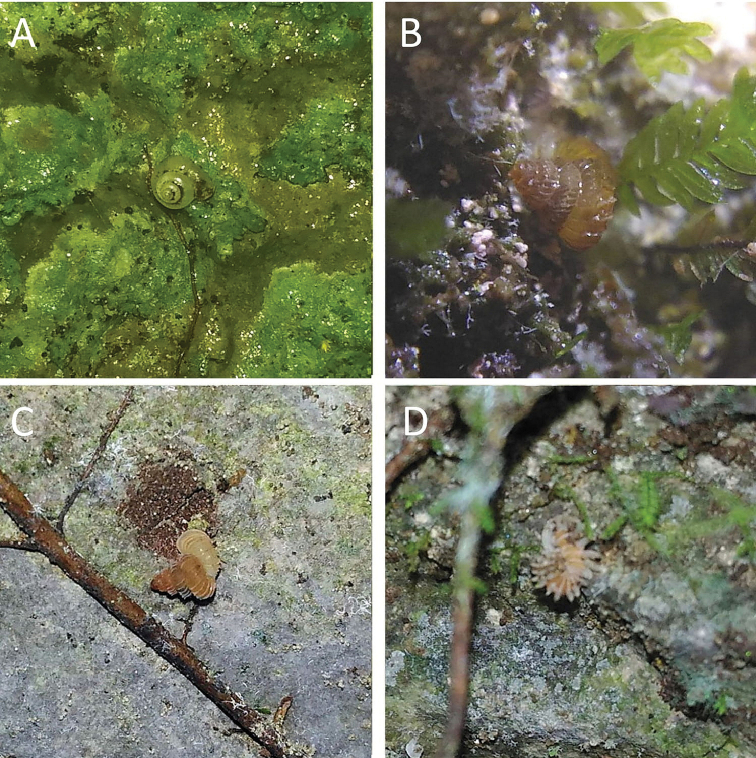
Living snails from Bau **A***Chamalycaeus specus* (Godwin-Austen, 1889) ME 11867 Lobang Angin **B***Plectostoma wallacei wallacei* Ancey, 1887 ME 8767 Gunung Kapor **C***Plectostoma austeni* (E. A. Smith, 1894) ME 8794 Gunung Batu **D***Plectostoma everetti* (E. A. Smith, 1893) ME 8793 Gunung Batu. All not to scale.

###### Pupisoma pulvisculum

Taxon classificationAnimaliaStylommatophoraValloniidae

(Issel, 1874)

23F75610-CF19-58ED-B896-F5DD3CA9D977

Figure 45D

Helix (Fruticicola) pulvisculum Issel, 1874: 406–407, pl. 5, figs 24–27.

####### Type locality.

“Borneo”.

####### Material examined.

Gunung Doya: ME 9108, ME 9151. Gunung Kapor: ME 8152, ME 9055, ME 9210, ME 9470. Lobang Angin: ME 9258.

####### Distribution in Borneo.

Sarawak: Kuching, Miri, and Limbang divisions. Sabah: Sandakan and West Coast divisions. ***Distribution elsewhere.*** Lombok, Indonesia (Smith 1899).

####### Remarks.

Only dry shells were found during the surveys. It differs from *Ptychopatula vermeuleni* Maassen, 2000 and *P. solemi* Maassen, 2000 by having minutely rugulose shell surfaces (visible at 40 × magnification). For further details, see Pilsbry (1920–1921: 30–31).

**Figure 49.**
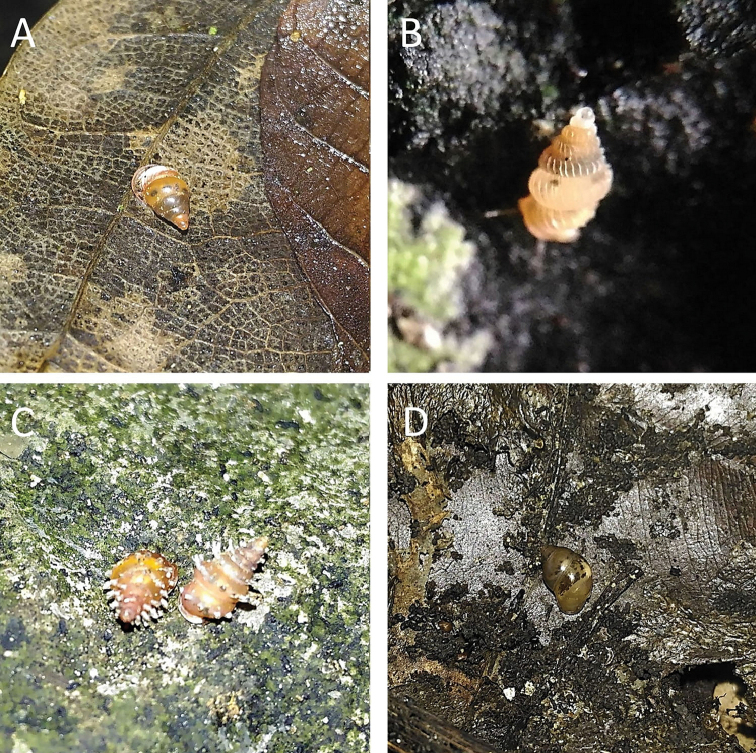
Living snails from Bau **A***Diplommatina adversa* (H. Adams & A. Adams, 1851) ME 8766 Gunung Kapor **B***Diplommatina concinna* H. Adams, 1872 ME 9137 Gunung Kapor **C***Diplommatina spinosa* Godwin-Austen, 1889 ME 8801 Gunung Batu **D***Pupina evansi* Godwin-Austen, 1889 ME 9053 Lobang Angin. All not to scale.

#### Family Vertiginidae Fitzinger, 1833

##### *Boysidia* Ancey, 1881

###### Boysidia salpinx

Taxon classificationAnimaliaStylommatophoraVertiginidae

F. G. Thompson & Dance, 1983

29B71445-42E9-5B92-88ED-646EB732C3F0

Figures 45E , 50B

Boysidia (Dasypupa) salpinx F. G. Tompson & Dance, 1983: 106–107, figs 2–6, 7, 8.

####### Type locality.

“Gunong Subis, a limestone massif about 40 mi SW of Miri, Niah area, Fourth Div., Sarawak, Borneo, 03°51’N, 113°45’E”.

####### Material examined.

Gunung Kapor: ME 2883, ME 8781, ME 9071, ME 9843.

**Figure 50.**
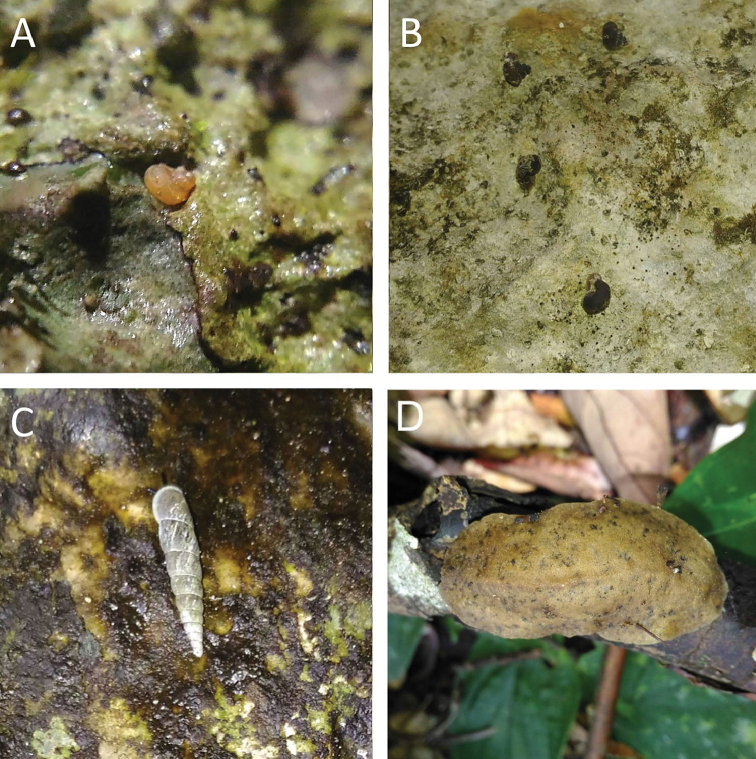
Living snails from Bau **A***Georissa bauensis* Khalik, Hendricks, Vermeulen & Schilthuizen, 2018 ME 8731 Lobang Angin **B***Boysidia salpinx* F. G. Thompson & Dance, 1983 ME 8781 Gunung Kapor **C***Phaedusa borneensis* (L. Pfeiffer, 1854) ME 8784 Gunung Kapor **D***Valiguna flava* (Heynemann, 1885) Uncat. Gunung Kapor. All not to scale.

####### Distribution in Borneo.

Sarawak: Kuching, Serian, and Miri divisions. Endemic to Borneo.

####### Remarks.

Living snails were observed foraging inside the shaded rock crevices and cave walls.

#### Clade Systellommatophora

**Family Veronicellidae Gray, 1840**

##### *Valiguna* Grimpe & Hoffmann, 1925

###### Valiguna flava

Taxon classificationAnimaliaSystellommatophoraVeronicellidae

(Heynemann, 1885)

8F496101-9C32-57C3-8E0A-26FDBF906F9F

Figure 50D

Vaginula flava Heynemann, 1885: 10–11, pl. 2, fig. 3.

####### Type locality.

“Borneo”.

####### Material examined.

Gunung Kapor.

####### Distribution in Borneo.

Sarawak: Kuching Division. Sabah: Interior, West Coast, and Tawau divisions. ***Distribution elsewhere.*** Sumatra, Peninsular Malaysia, and Singapore (Heynemann 1885; Forcart 1973; Low 2014).

**Figure 51.**
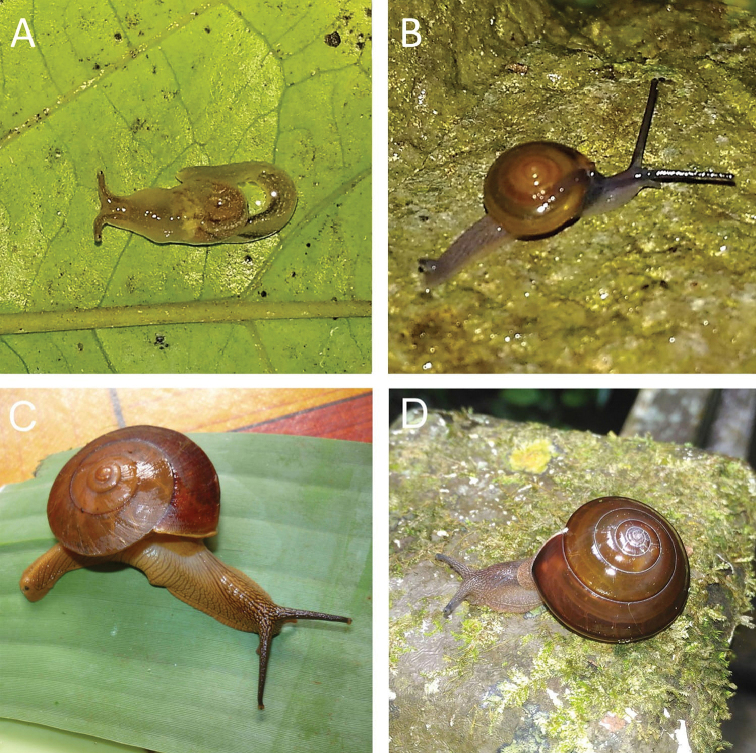
Living snails from Bau **A***Damayantia carinata* Collinge, 1901 Uncat. Gunung Kapor **B***Macrochlamys tersa* (Issel, 1874) ME 8966 Gunung Kapor **C***Hemiplecta densa* (A. Adams & Reeve, 1850) ME 4724 Gunung Kapor **D***Vitrinula glutinosa* (Metcalfe, 1851) ME 8750 Lobang Angin. All not to scale.

####### Remarks.

Living snails were observed foraging among leaf litter and plant debris near the cliff in a lowland limestone forest. However, no specimens were collected during the surveys. For further details, see Schilthuizen and Liew (2008: 292–293).

**Figure 52.**
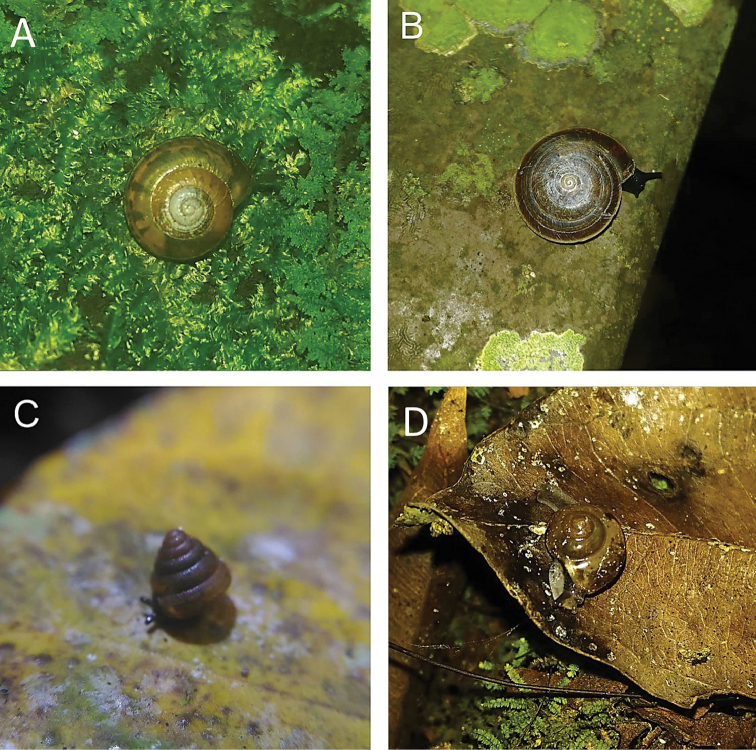
Living snails from Bau **A***Videna timorensis* (Martens, 1867) ME 8984 Lobang Angin **B***Videna bicolor* (Martens, 1864) ME 8732 Lobang Angin **C***Kaliella barrakporensis* (L. Pfeiffer, 1852) ME 9026 Gunung Kapor **D***Microcystis dyakana* Godwin-Austen, 1891 ME 8969 Gunung Kapor. All not to scale.

**Figure 53.**
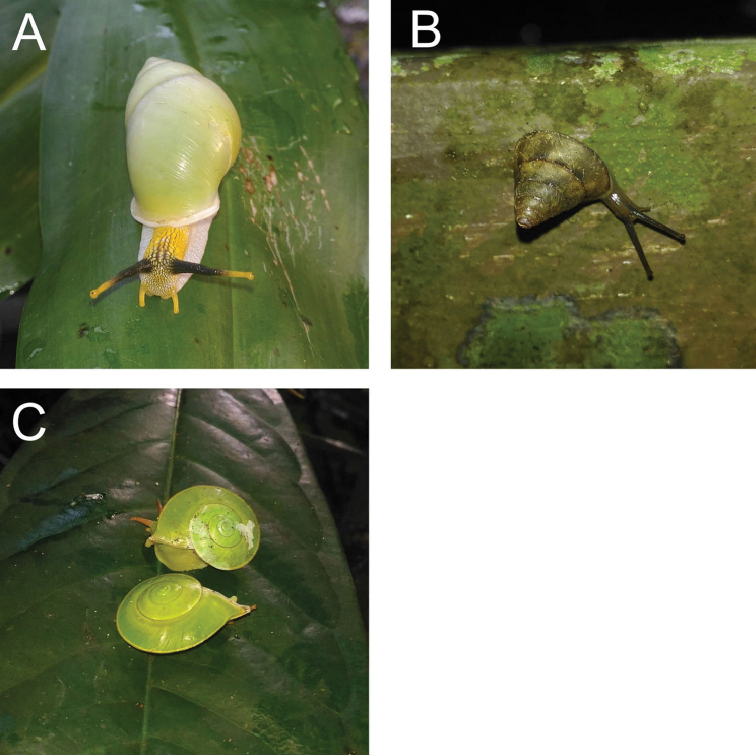
Living snails from Bau **A**Amphidromus cf. similis Pilsbry, 1900 ME 8756 Gunung Kapor **B***Ganesella acris* (Benson, 1859) ME 8977 Lobang Angin **C***Rhinocochlis nasuta* (Metcalfe, 1851) ME 8885 Lobang Angin. All not to scale.

**Figure 54.**
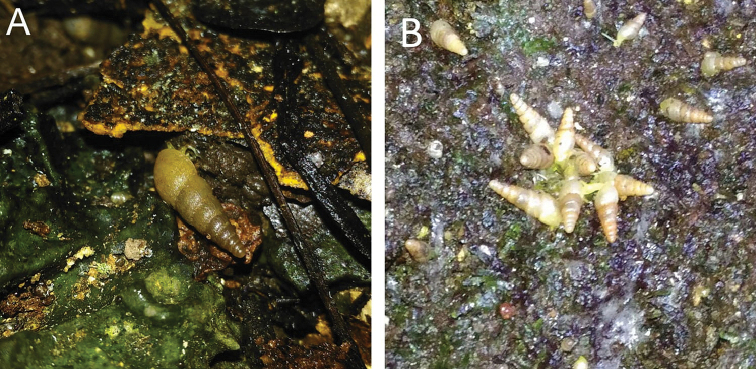
Living snails from Bau **A***Paropeas achatinaceum* (L. Pfeiffer, 1846) ME 8785 Gunung Kapor **B***Allopeas gracile* (T. Hutton, 1834) ME 2942 Gunung Kapor. All not to scale.

## Supplementary materials

Supplementary material 1 Taxa names and authorships in the manuscript that do not follow MolluscaBase Data type text (.docx file) https://binary.pensoft.net/file/536843This dataset is made available under the Open Database License (http://opendatacommons.org/licenses/odbl/1.0/). The Open Database License (ODbL) is a license agreement intended to allow users to freely share, modify, and use this Dataset while maintaining this same freedom for others, provided that the original source and author(s) are credited.Mohammad Effendi bin Marzuki, Thor-Seng Liew, Jayasilan Mohd-Azlan
