# Supplementary material for: Land snails and slugs of Bau limestone hills, Sarawak (Malaysia, Borneo), with the descriptions of 13 new species
Source: Zookeys. 2021 Apr 27;1035:1–113. doi: 10.3897/zookeys.1035.60843 (PMC8096804; doi:10.3897/zookeys.1035.60843)
Supplement: Supplementary material 1 — Taxa names and authorships in the manuscript that do not follow MolluscaBase [file zookeys-1035-001-s001.docx]

**Appendix 1. Taxa names and authorships in the manuscript that do not follow MolluscaBase.**

We checked MolluscaBase and follow the classification of most of the taxa and confirmed the authorships. We corrected all the mistakes based on the cross-check with MolluscaBase, except the following species:

(1) The publication year and authorship of the some of the species at MolluscaBase was not accurate. Hence, we did not change the authorship and year in our manuscript:

- *Pincerna globosa* (H. Adams, **1870**)
- *Leptopoma sericatum* (L. Pfeiffer, **1851**)
- *Japonia barbata* (Pfeiffer, **1855**)
- *Opisthoporus birostris* (L. Pfeiffer, **1854**)
- *Opisthoporus euryomphalus* (L. Pfeiffer, **1856**)
- *Pterocyclos tenuilabiatus* (Metcalfe, **1851**)
- Kaliella scandens (Cox, **1871**) vs. *Liardetia scandens* (Cox, **1872**)
- *Rhinocochlis nasuta* (Metcalfe, **1852**)
- *Everettia cutteri* (H. Adams, **1870**) vs. *Macrochlamys cutteri* H. Adams, **1871**
- *Exrhysota brookei* (**A. Adams & Reeve, 1848**) vs. *Exrhysota brookei* (**Wiegmann, 1898**)

(2) The generic names for some species in MolluscaBase are based on very old works without new updates from recent publications. The taxonomy of some of the genera was not revised and MolluscaBase information reflects the unclear taxonomy for some genera:

- *Japonia* vs. *Lagochilus*
  - *Japonia* was used in this manuscript.
  - For Bornean species, all the recent publications use *Japonia* instead of *Lagochilus*. Given that (1) there are no comprehensive revisions of both genera in this region to date and (2) to maintain the consistency of our past and current works on the land snail in this region until the materials sufficient for the revision of these taxa.
    - *Japonia metcalfei* (Issel, 1874)
    - *Japonia rabongensis* (E. A. Smith, 1895)
- *Pupina* vs. *Tylotoechus*
  - *Pupina* was used in this manuscript.
  - For Bornean species, all the recent publications use *Pupina* instead of *Tylotoechus*. Given that (1) there are no comprehensive revisions of both genera in this region to date and (2) to maintain the consistency of our past and current works on the land snail in this region until the materials sufficient for the revision of these taxa.
    - *Pupina doriae* Godwin-Austen, 1889
- *Kaliella* vs. *Liardetia* and *Sitala*
  - *Kaliella* was used in this manuscript.
  - For Bornean species, all the recent publications use *Kaliella* instead of *Liardetia* or *Sitala*. The most recent revision of these taxa in Borneo was Vermeulen, J. J., Liew, T. S., & Schilthuizen, M. (2015). Additions to the knowledge of the land snails of Sabah (Malaysia, Borneo), including 48 new species. ZooKeys 531: 1-139. <https://doi.org/10.3897/zookeys.531.6097>
    - *Kaliella busauensis* (Smith, 1895) vs. Sitala busauensis E. A. Smith, 1895
    - Kaliella scandens (Cox, 1871) vs. Liardetia scandens (Cox, 1872)
    - *Kaliella micula* (Mousson, 1857) vs. Liardetia micula (Mousson, 1857)
- *Macrochlamys* vs. *Lamprocystis*
  - *Macrochlamys* was used in this manuscript.
  - For Bornean species, all the recent publications use *Macrochlamys* instead of *Lamprocystis*. Given that (1) there are no comprehensive revisions of both genera in this region to date and (2) to maintain the consistency of our past and current works on the land snail in this region until the materials sufficient for the revision of these taxa.
    - *Macrochlamys infans* Pfeiffer, 1854 vs. Lamprocystis myops (Dohrn & C. Semper, 1862)
- *Everettia cutteri* (H. Adams, 1870) vs *Macrochlamys cutteri* H. Adams, 1871
  - *Everettia cutteri* (H. Adams, 1870) was used in this manuscript.
  - Based on the most recent revision of *Everettia* in this region. Liew, T. S., Schilthuizen, M., & Vermeulen, J. J. (2009). Systematic revision of the genus *Everettia* Godwin-Austen, 1891 (Mollusca: Gastropoda: Dyakiidae) in Sabah, northern Borneo. Zoological Journal of the Linnean Society 157(3): 515-550.
- *Videna bicolor* (Martens, 1864) vs. *Geotrochus bicolor* (Martens, 1864)
  - *Videna bicolor* (Martens, 1864) was used in this manuscript.
  - Based on the most recent revision of *Geotrochus* in this region. Vermeulen, J. J., Liew, T. S., & Schilthuizen, M. (2015). Additions to the knowledge of the land snails of Sabah (Malaysia, Borneo), including 48 new species. ZooKeys 531: 1-139. <https://doi.org/10.3897/zookeys.531.6097>
- *Ptychopatula orcella* (Stoliczka, 1873) vs. *Pupisoma orcella* (Stoliczka, 1873)
  - Although Hausdorf (2007) suggested the use of *Pupisoma*, *Ptychopatula orcella* (Stoliczka, 1873) was used in this manuscript. Hausdorf, B. (2007). Revision of the American Pupisoma species (Gastropoda: Pupilloidea). Journal of Natural History, 41(21-24), 1481-1511.
  - For Bornean species, all recent publications use *Pupina* instead of *Tylotoechus*. Given that (1) there are no comprehensive revisions of both genera in this region to date and (2) to maintain the consistency of our past and current works on the land snail in this region until the materials sufficient for the revision of these taxa.
